# Genotyping-by-sequencing identifies date palm clone preference in agronomics of the State of Qatar

**DOI:** 10.1371/journal.pone.0207299

**Published:** 2018-12-05

**Authors:** Gaurav Thareja, Sweety Mathew, Lisa S. Mathew, Yasmin Ali Mohamoud, Karsten Suhre, Joel A. Malek

**Affiliations:** 1 Bioinformatics Core, Weill Cornell Medicine–Qatar, Doha, Qatar; 2 Genomics Core, Weill Cornell Medicine–Qatar, Doha, Qatar; National Cheng Kung University, TAIWAN

## Abstract

Understanding the genetic diversity in a crop population is key to its targeted breeding for desired traits, such as higher yields, better fruit quality and resistance to disease and changing climates. Date fruits represent a major crop in the Middle East and are key to achieving future food independence in arid countries like Qatar. We previously determined the genome of the date palm *Phoenix dactylifera* and showed that date palm trees world-wide divide into two distinct subpopulations of Eastern and Western origins. Here we applied a resource of SNPs from 179 commercially available date fruits to assess the genetic diversity of date palm trees grown in the State of Qatar. We found that palm trees in Qatar are mainly of Eastern origin, and that their genetic diversity doesn’t associate with regions of the State. Together with targeted genetic assays, our resource can be used in the future for date palm cultivar identification, to aid selecting suitable cultivars for targeted breeding, to improve a country’s date palm genetic diversity, and to certify the origin of date fruits and trees.

## Introduction

Genetic diversity of plants is crucial to ensure sufficient food production to an ever-growing world population. Genetic diversity provides researchers with a resource that enables development of improved crop cultivars with desired traits, such as higher yields and disease resistance [1, 2]. The loss of cultivable land due to soil erosion, climate change and alternative usage of land for supporting rapid urbanization is motivating the development of high yield and drug resistant cultivars [3]. In the past, morphological features like leaf length and plant height were extensively used for cultivar selection. However, such morphological features have limitations, as they require plants to be fully grown before these features can be assessed and used for classification [4]. The advent of molecular biology-based technologies, especially genome-sequencing, has enabled rapid identification of plant cultivars based on genetic variation. These technologies include the use of random amplified polymorphic DNA (RAPD) markers [5], simple sequence repeats (SSR) [6], amplified fragment length polymorphisms (AFLP) [7], and single nucleotide polymorphisms (SNP) [8–10].

With reducing cost of next generation sequencing (NGS), it has become easier to deeply genotype plant samples at a whole-genome level, and thus to obtain a catalogue of cultivar-specific genetic markers (SNPs, STRs etc.). It is not yet economical to sequence entire genomes of each accession for cultivar and trait identification. Reduced representation sequencing of a limited number of genomic regions, including approaches such as exome sequencing, genotyping-by-sequencing (GBS) and transcriptome sequencing [11], provide inexpensive alternatives to discover and genotype large numbers of SNPs and are already extensively used in plant breeding programs [12–14].

In Middle Eastern cultures, the date palm tree *Phoenix dactylifera* is considered to constitute the ‘elixir of life’, since it is a source of medicine, food and even building materials to natives of the Arabian Peninsula. The earliest signs of the cultivation of date palm trees dates back to 4000 B.C. [15]. Date palm trees can survive in extremely harsh conditions and require only moderate care, making it a unique and well-suited crop for the region. Major constraints to date palm cultivation in the region are pests and diseases that infect date palm trees, causing considerable economic and ecological losses [16–18]. In addition, increasing desertification and decreasing water resources pose serious threats to agricultural biodiversity. Presently, one of the most effective means to develop new cultivars is to identify inherent genetic resistant traits in cultivars and interbreed these with cultivars of desired traits [19, 20]. As date palm trees have long generation times of six years and more [21], it is of paramount importance to identify cultivars with desirable features early on, using molecular and genetic signatures to aid the selection process.

In this study, we sequenced and created a hierarchical clustering tree comprising DNA obtained from 179 geographically diverse date fruits, leaves from 55 *P*. *Dactylifera* trees grown in Qatar, and leaves obtained from 19 other *Phoenix* species. The date fruits have been collected world-wide and represent arguably the most genetically diverse collection of date samples available to date. We used the resulting genetic data set to study the genetic diversity of date palm trees in the State of Qatar, showcasing how this resource can be used as a general tool for the identification and classification of date fruits and date palm trees.

## Material and methods

### Sample collection, sequencing, alignment and variant calling

DNA collection was performed by ad-hoc collection, aimed at capturing a representative sampling from each of the municipality regions of Qatar. Young leaves were collected from 55 date palm trees with their locations tagged by GPS. Genotyping-by-Sequencing (GBS) libraries were constructed and sequencing was performed as described before [22]. Briefly, size selected libraries of 350 to 550 bps were amplified using PCR. Twenty-four samples were then pooled in a single lane of a HiSeq 2500 (Illumina, USA) and paired-end sequencing was performed in accordance with the manufacturer’s protocols S1 Table.

The GBS reads (DNA obtained from leaves) were aligned to the date palm reference genome (V3) [23] using the BWA aligner with default settings [24]. SNP calling was performed using the SAMTOOLS mpileup command [25] to output genotype likelihoods, and the bcftools command was used to convert these likelihoods to base calls in VCF format [26]. Insertions/deletions were not used in the analysis.

### Quality control of variant calls

We extracted SNPs in regions covered by our GBS protocol for 192 common date palm cultivars (date fruits) and other 19 *Phoenix* species from our in-house date palm genetic resource (Unpublished data) S2 and S3 Tables. For each variant set (date fruit, leaves, other *Phoenix* species), we marked genotypes with less than 10X coverage as missing in order to have sufficient coverage for calling heterozygous variants using VCFtools. Further, each variant set was processed using VCFtools to remove SNPs with missingness > 0.4. Then, samples with an overall missingness > 0.3 were removed. Finally, only bi-allelic SNPs with missingness < 0.1 and Hardy-Weinberg exact test p-value >10^−6^ were retained for analysis.

### Clustering and visualization

Pairwise identity-by-state (IBS) distances were computed using PLINK v1.9 [27]. Pairwise distances were defined as (1-IBS) and hierarchical Ward’s clustering was performed using the *hclust* function (method = "ward.D"), as implemented in the *stats* package in R version 3.4.3 [28]. The clustering output was stored using the *write*.*tree* function as implemented in *ape* package [29]. The hierarchical cluster trees were then visualized using the iTOL web browser [30]. The hierarchical cluster tree was rooted using the *Phoenix* outgroup samples.

### Informative SNPs selection

Polymorphism information content (PIC) was computed to rank SNPs based on their information content [31] using an in-house Perl script. PIC for any given SNP *i* is defined as

$${PIC}_{i}=1-(a_{i}^{2}+{\left(1-a_{i}\right)}^{2})-2*a_{i}^{2}*{\left(1-a_{i}\right)}^{2}$$

where *a*_*i*_ represents minor allele frequency (MAF) of SNP *i*.

To reduce the number of SNPs, a sub-panel with a high PIC value of ≥ 0.37 was created (the maximum PIC for bi-allelic markers is 0.375). These selected highly informative SNP markers were used to calculate a (1 –IBS) pairwise distance matrix. A Mantel test, as implemented by mantel.rtest function in ade4 package, was used to assess similarity between distance matrices [32].

## Results

### Hierarchical cluster tree of date palm cultivars

We used a high coverage (20X – 40X) re-sequenced data resource of 191 date palm cultivars at the whole-genome level. In addition, this data resource also contained 19 *Phoenix* species out-group samples (**Unpublished data**). From this resource, we selected only SNPs overlapping genomic regions covered by genotyping-by-sequencing (a reduced representation sequencing approach) for both date palm cultivars and *Phoenix* species out-group samples. After extensive quality filtering (see methods), we were left with 179 date palm cultivars and 19 *Phoenix* species out-group samples, yielding a final dataset containing 198 samples and 13,803 high quality SNPs. These high-quality variants were used to construct a hierarchical cluster tree to understand the overall genetic membership of the date palm cultivars.

**Fig 1:**
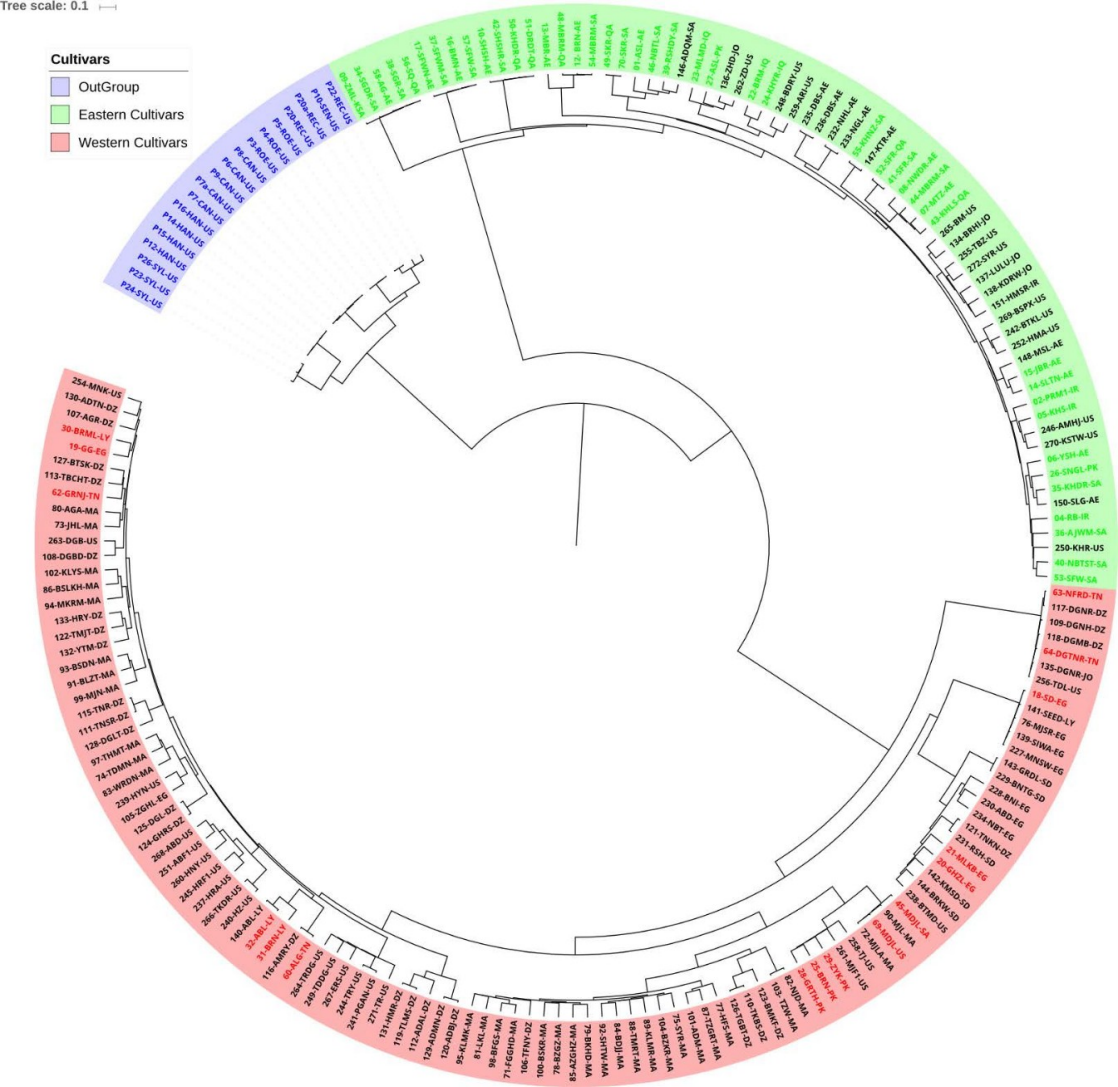
A rooted hierarchical cluster tree showing the relationship between 179 *Phoenix dactylifera* cultivars and samples from 19 other Phoenix species using 13,803 markers. The edge length represents the (1-Identity-by-state) distances. The highlighted clusters in the tree are samples from other *Phoenix* Species (yellow background), Eastern cultivars originating from the Arabian Gulf (white background) and Western cultivars originating from North Africa (blue background). Colored labels indicate that these variants have already been classified in our previous work [22]. Green labels indicate previously identified Eastern cultivars, red labels indicate Western cultivars, and black labels are new in this study. All previously reported variants cluster in the present much larger study, and in exactly the same way, showing the robustness of this approach.

The cluster tree showed three predominant clusters at the highest level (Fig 1). As expected, all 19 outgroup (non-Dactylifera) *Phoenix* species clustered together. This outgroup cluster was used to root the tree. Date palm cultivar sub population categorizations (Western = North African cultivars, Eastern = Arabian cultivars) were obtained for a subset of 60 cultivars from our previous study [22]. Based on shared membership with annotated cultivars in the same clusters, we then categorized the remaining 119 cultivars into two sub populations, thus extending our current knowledge of genetic diversity in Date Palm cultivars. Out of 119 cultivars that were new in this study, 94 (78.99%) cultivars were from the Western group.

### Informative marker selection

In the hierarchical tree presented in Fig 1, all date palm cultivars that were used in our previous study correctly separated again into two sub populations, confirming our previous findings in this larger data set. To further reduce the number of markers required to classify the cultivars, we extracted 6,332 independent SNPs (LD < 0.1) and then ranked these markers based on the polymorphism information content (PIC). We selected 1,133 SNPs with a PIC value greater than 0.37 (see methods). The pairwise IBS distance matrix computed using these highly informative 1,133 SNPs showed a high correlation of R = 0.976 with the matrix constructed using all 13,803 SNPs.

### Geographic sampling and sequencing of date palm trees

**Fig 2:**
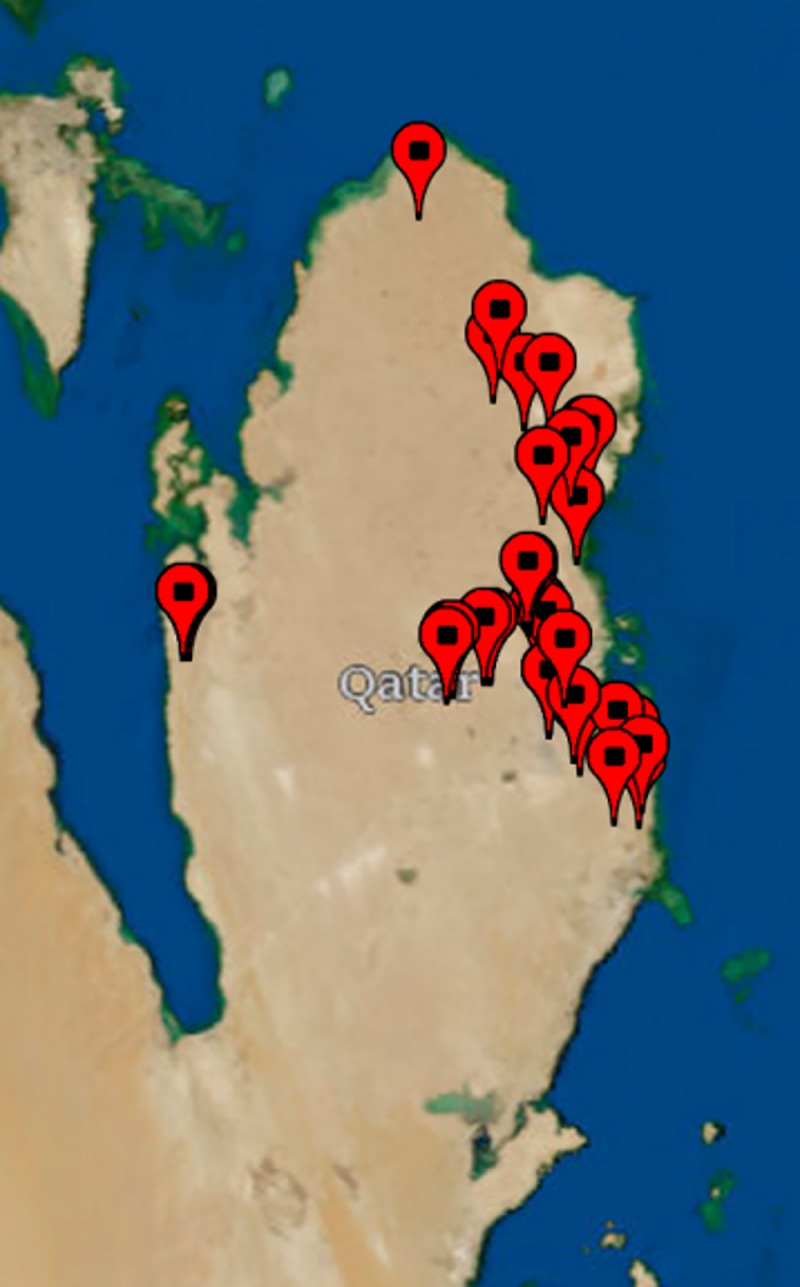
GPS locations of 55 date palm trees from which leaves have been collected in the State of Qatar. Collection sites around the Eastern coast line are representative for municipalities in which date palm trees are grown. Few trees are found in the Western part of Qatar and in the desert region to the South. Map was created using U.S. Geological Survey (USGS) Topological Imagery as a base map in MATLAB (The Mathworks, Natick, USA).

The State of Qatar comprises seven administrative divisions (municipalities), namely Doha, Al Khor, Al Daayen, Al Shamal, Umm Salal, Al Wakrah and Al Rayyan. We collected leave samples from mature date palm trees growing in public parks, farms, and on road-sides, referenced using global positioning system (GPS) coordinates (Fig 2 and S1 Table). We performed genotyping-by-sequencing on all these tree samples. After quality filtering, 55 samples satisfied all quality criteria. We extracted 1,133 highly informative markers from the above analysis and merged the data of the 55 municipality leave samples with 179 date fruit and 19 outgroup leave samples and performed hierarchical clustering this dataset (S1 File: VCF file containing variant calls for 1,133 highly informative variants and 253 samples; S2 File: Images of the 55 municipality trees showing morphological characteristics of the trees collected in this study; Images of the date fruits used in the study are available as supplement in article by Stephan et al. [33]).

### Characterization of subgroups of date palm trees

**Fig 3:**
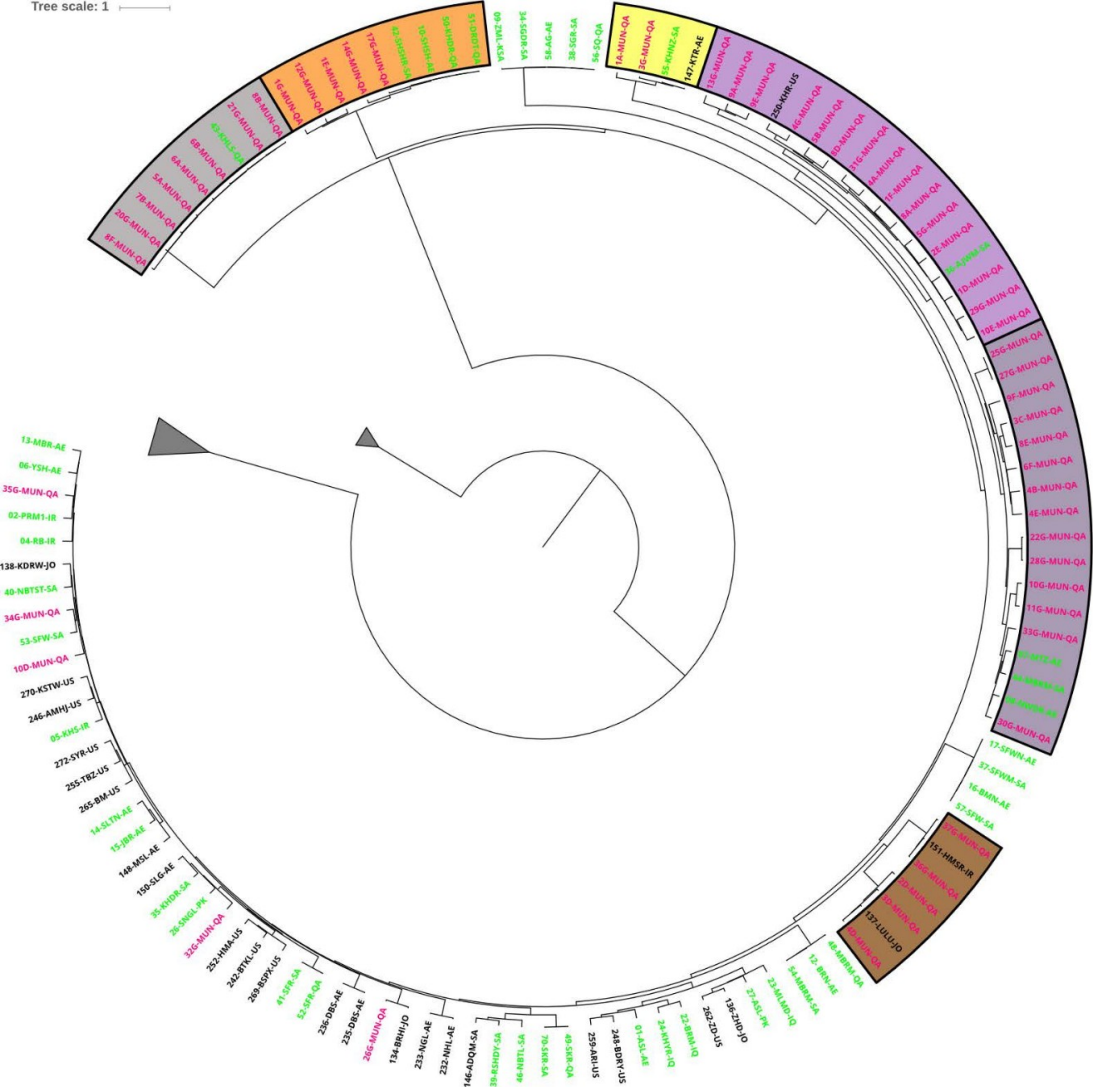
A rooted hierarchical cluster tree showing the relationship between date palm cultivars and date palm trees from the state of Qatar. The tree highlights the clustering of date palm trees from the State of Qatar with common date palm cultivars. The edge length represents the (1-Identity-by-state) distances. For clarity, Western cultivars (larger triangles) and outgroup (small triangles) are collapsed as no municipality samples cluster in one of them. Date palm trees collected in the State of Qatar are labeled in pink. The labels are colored based on their annotations reported in our previous work [22]. Green labels indicate cultivars that were annotated as Eastern cultivars, and black labels are new cultivars used in this study. The complete tree is presented in S1 Fig.

DNA obtained from one date palm tree collected in Qatar clustered with the out-groups. This tree was visually confirmed as *P*. *canariensis*. All other samples from date palm trees in Qatar clustered with Eastern cultivars originating in the Arabian Gulf, indicating that there is only a limited genetic diversity of date palm trees in the State of Qatar. These date palm trees were distributed over six clusters, where each cluster contained a known date palm cultivar (Fig 3 and S1 Fig). Fifteen (27%) trees fell into a cluster that contains the *Ajwa medina* cultivar from Saudi Arabia and Khir from the US, and 14 (25%) date palm trees fell in a cluster that contains the *Mumtaza* and *Nawader* cultivars from UAE and *Mabroom* from Saudi Arabia. The remaining date palm trees cluster with common date palm cultivars, including *Khalas*, *Shieshi Rotab*, *Sheshe (an alternate spelling of Shieshi)*, *Khudry*, *Kheneizi*, *Harmati Sadrati* and *Lulu*. None of the groups were enriched with trees from any single municipality. Date palm trees are sometimes reproduced through vegetative propagation. For the purpose of estimating the genetic variance in the population, such trees have to be considered as being genetically identical to their seed propagated ancestors.

## Discussion and conclusion

The State of Qatar has identified agriculture as an important pillar in its National Food Security Program (QNFSP - http://www.qnfsp.gov.qa/). Date palm trees are the most abundant permanent crops grown in the country, due to their ability to withstand the harsh climatic conditions that are prevalent on the Arabian Peninsula [34]. In a previous work from our group, we subdivided 70 common date palm cultivars from around the world into Eastern (Arabian Gulf) and Western Cultivars (North Africa), using genotyping-by-sequencing and phylogenetic tree analysis. We showed that approximately 36% of alleles are private to either one of the two sub-populations, thus highlighting the high genetic diversity within the sub-populations [22]. In this present work, we extended our previous genetic resource by including an additional 119 cultivars in the study and 19 *Phoenix* outgroup species as a reference. We used identity-by-descent pairwise distances to hierarchically cluster these 179 cultivars with 19 samples from the Phoenix outgroup species. The hierarchical cluster tree showed three clusters. Overall, 9% of the new cultivars included in this study clustered with Western cultivars, thus reducing the overrepresentation of eastern cultivars in our previous work. Currently, our resource has 69 Eastern cultivars and 110 Western cultivars. As this resource contains SNP calls from the broadest diversity of date palms to-date, it can be used for date palm cultivar identification using the same subset of SNPs as identified here.

Genotyping-by-sequencing (GBS), a reduced representation sequencing approach, is often used as the method of choice for plant breeding [13]. In genomic regions covered by GBS, a subset of 1,133 independent and informative markers were selected using polymorphism information content (PIC). This subset of markers shows a genetic distance matrix correlation coefficient of 0.976 with a similar matrix constructed using 13,803 markers. A report from soybean cultivars reported that using only 20 SNPs, they were able to classify 9,445 cultivar pairs [35]. Another report identified 48 SNP set for grapevine cultivar identification [13].

After selecting these 1,133 independent markers, we moved our focus on understanding genetic diversity of local date palm trees in the State of Qatar. Previous studies from the State of Qatar, addressing the genetic diversity using simple-sequence repeat (SSR) markers in date palm trees addressed the question about diversity among date palm cultivars either growing in localized farms or in different farms across Qatar. However, these studies did not address the genetic diversity of the local flora which includes date palm trees growing on roadside or in public parks. Discussions with authorities in Qatar and our own observations suggest that most of the purposefully planted trees along the roads are not seed grown, but rather vegetatively grown from single or few cultivars. The hierarchical clustering-based tree revealed that Qatar date palm trees only grouped within Eastern cultivars and there were none with similarity to Western Cultivars. We did not find any evidence of genetic diversity in date palm trees structured according by municipalities. In the context of our results presented here, vegetative propagated trees will likely cluster very close to one another. On the other hand, seed grown cultivars would cluster with less similarity, as their genetics would include 50% from the novel father. Therefore, distances in the hierarchical clustering-based tree don’t necessary reflect evolutionary distances. The trees formed six clusters with more than > 50% of trees lying in groups 4 and 5 which include soft dates. Biochemically, soft dates predominantly contain reducing sugars (glucose and fructose) [36] and can tolerate humidity and require less heat [37]. We also observed some samples with high divergence from known cultivars included in the comparison. These may represent less popular cultivars or seed grown trees. This limited diversity and predominance of only eastern cultivars of date palm trees is likely the preference for certain date types in the local market. We believe that one of the implications of this study is to provide a broader overview of the genetic diversity within the country. Based on this information, authorities can take informed decisions on future plantings of trees, increase the overall genetic diversity, possibly including rare variants that are threatened by pests in other countries. For instance, planting of trees from the other major genetic subgroups of date palms [22] would dramatically change the genetic landscape within the country. While high quality fruits are important for commercial aspects of agriculture, generally increasing the genetic diversity will provide a pool of novel genes from which to select new characteristics, such as traits related to disease or abiotic stress resistance.

In conclusion, understanding the genetic diversity and cultivar classification for date palm will help authorities in planting high quality fruit producing and genetically diverse cultivars, while mitigating possible future outbreaks of diseases. We provide a genetic resource for date palm cultivar identification. This resource used with reduced representation genotyping-by-sequencing data was able to identify limited genetic variability and predominance of Eastern cultivars across municipalities in the State of Qatar. The diversity estimates were independent of municipalities. In the future, diversification of the local flora using molecular genetic markers for date palm cultivars identification will aid in developing the agriculture resources in the State of Qatar.

## Supporting information

S1 File VCF file containing variant calls for 1,133 highly informative variants and 253 samples. The 253 samples contain 179 date palm cultivars, 19 other *Phoenix* species and 55 date palm trees sampled in the State of Qatar.
(GZ) Click here for additional data file.

S2 File Images of 55 Date Palm trees sampled from municipalities in the State of Qatar. The images depict morphological characteristics of date palm trees growing in the State of Qatar.
(ZIP)

S1 Table Global Positioning System (GPS) coordinates and site details for the collection of 55 date palm trees sampled in the State of Qatar (leaves). (XLSX)

S2 Table Annotation and country of origin for 179 date palm cultivars (fruits). (XLSX)

S3 Table Annotation of the 19 *Phoenix* species (outgroup). (XLSX)

S1 Fig A rooted hierarchical cluster tree showing the relationship between date palm cultivars and date palm trees from the state of Qatar. The tree highlights the clustering of date palm trees from the State of Qatar with common date palm cultivars. Date palm trees collected in the State of Qatar are labeled in pink. The edge length represents the Identity-By-State distances. The highlighted clusters in the tree: Outgroup (samples from other *Phoenix* Species); Eastern cultivars (green) originating in Arabian Gulf and Western cultivars originating in North Africa (blue). The labels are colored based on their annotations from our previous work [22]. Green labels indicate cultivars were annotated as Eastern cultivars; Red labels indicate cultivars were annotated as Western cultivars; and black labels are new cultivars used in this study.
(TIFF)
