# Supplementary figures and images for: Genotyping-by-sequencing identifies date palm clone preference in agronomics of the State of Qatar
Source: PLoS One. 2018 Dec 5;13(12):e0207299. doi: 10.1371/journal.pone.0207299 (PMC6281209; doi:10.1371/journal.pone.0207299)

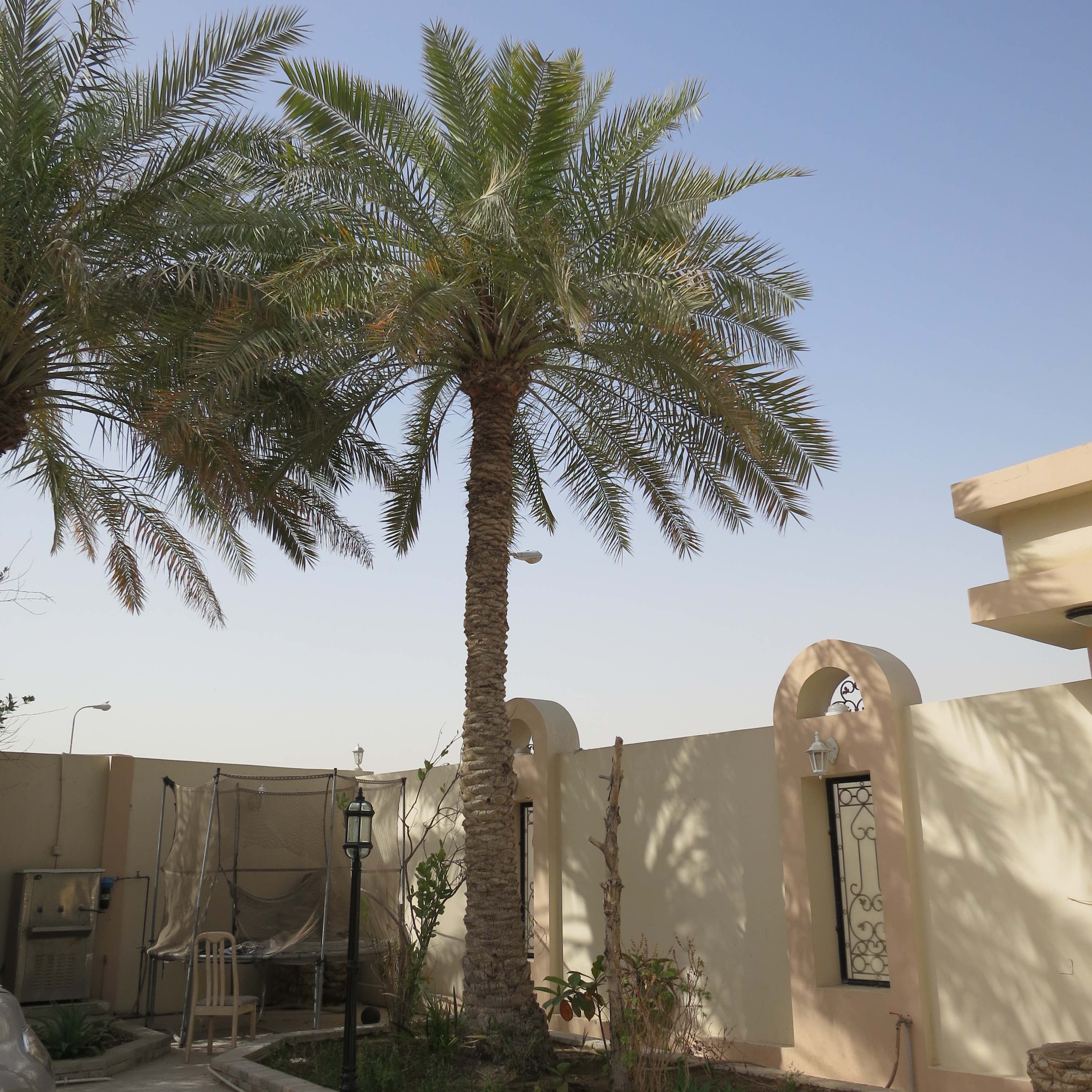

Supplement: S2 File — The images depict morphological characteristics of date palm trees growing in the State of Qatar. (ZIP) [file pone.0207299.s002.zip › Additional_Dataset_2_reduced/004 A.jpg]

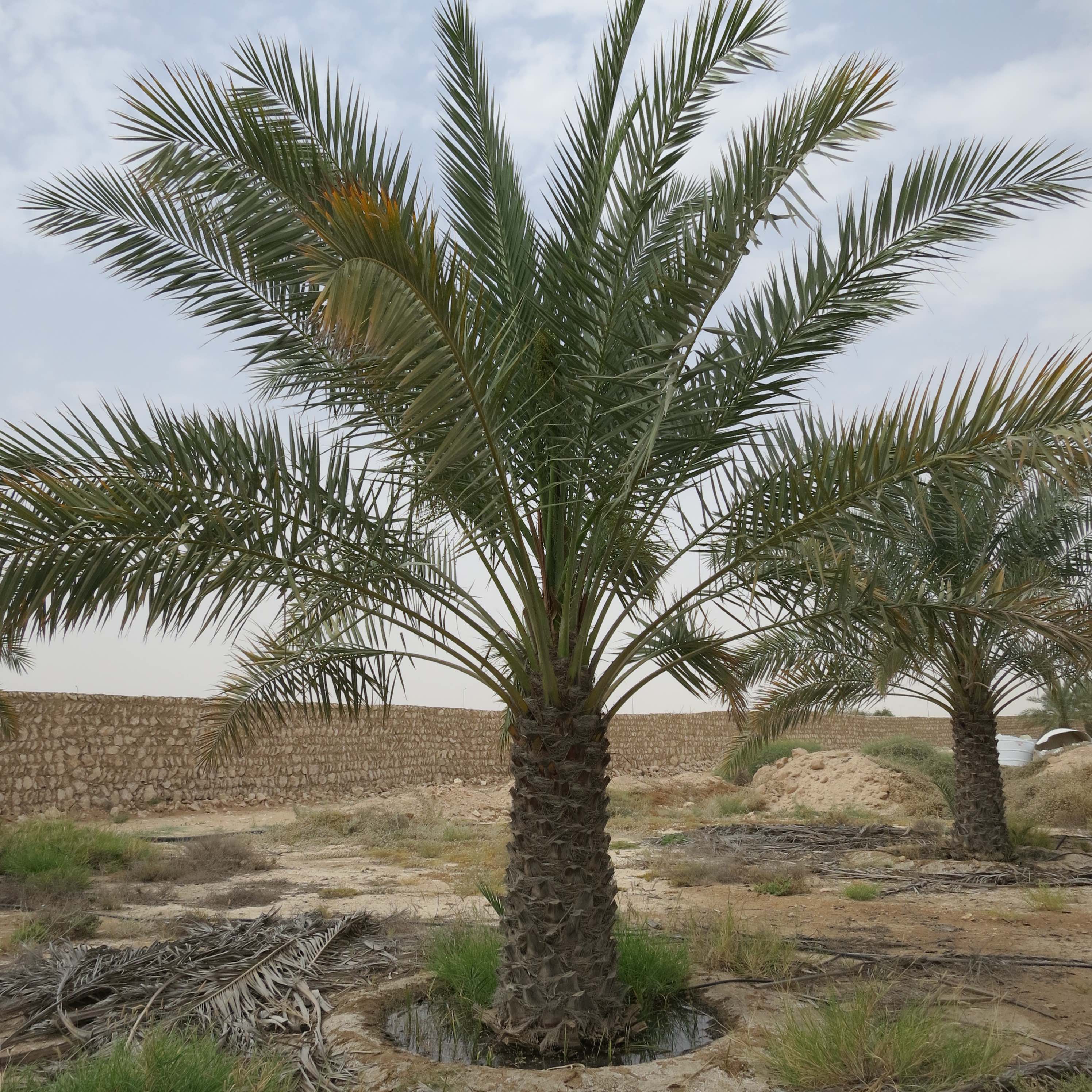

Supplement: S2 File — The images depict morphological characteristics of date palm trees growing in the State of Qatar. (ZIP) [file pone.0207299.s002.zip › Additional_Dataset_2_reduced/026 G.jpg]

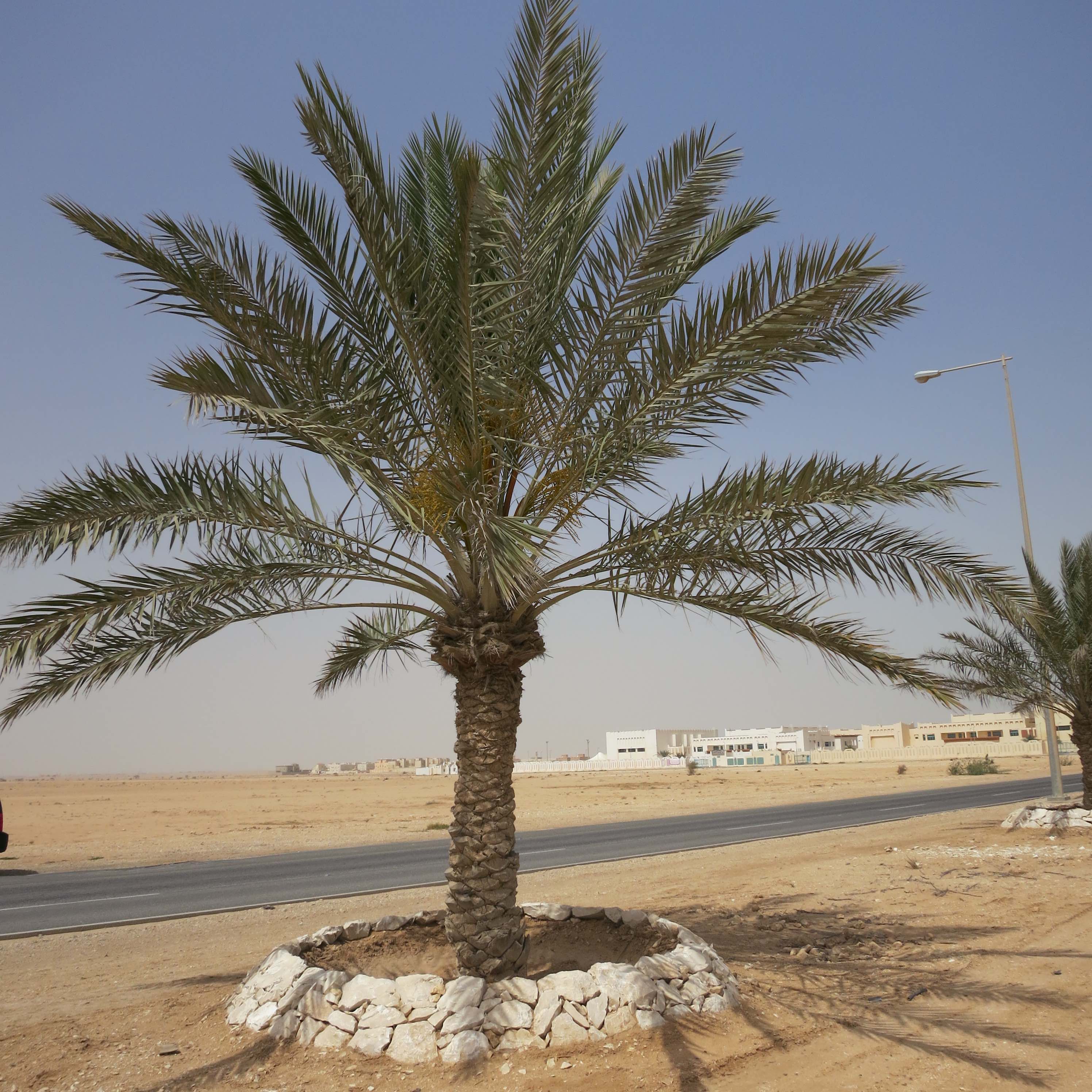

Supplement: S2 File — The images depict morphological characteristics of date palm trees growing in the State of Qatar. (ZIP) [file pone.0207299.s002.zip › Additional_Dataset_2_reduced/006 B.jpg]

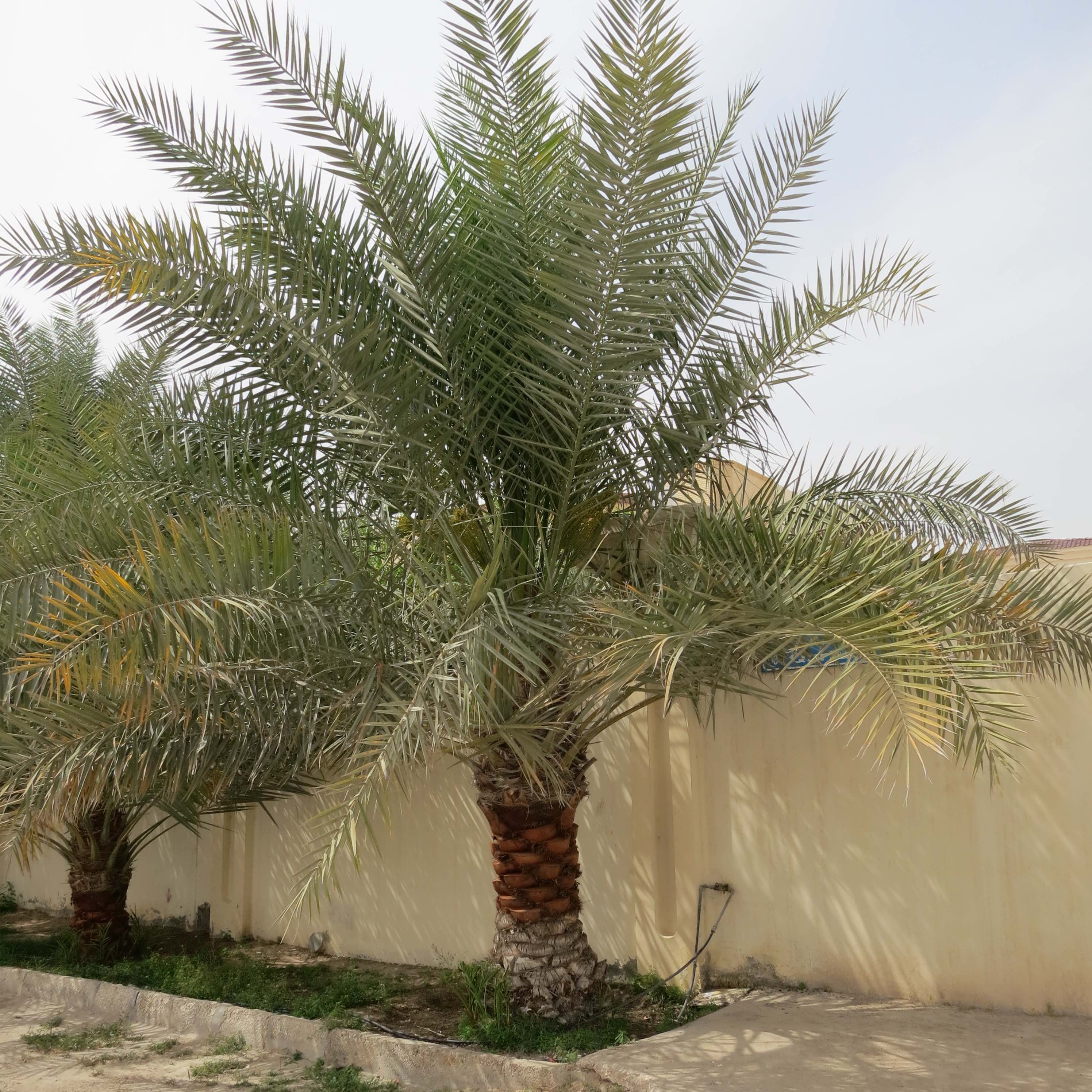

Supplement: S2 File — The images depict morphological characteristics of date palm trees growing in the State of Qatar. (ZIP) [file pone.0207299.s002.zip › Additional_Dataset_2_reduced/008 F .jpg]

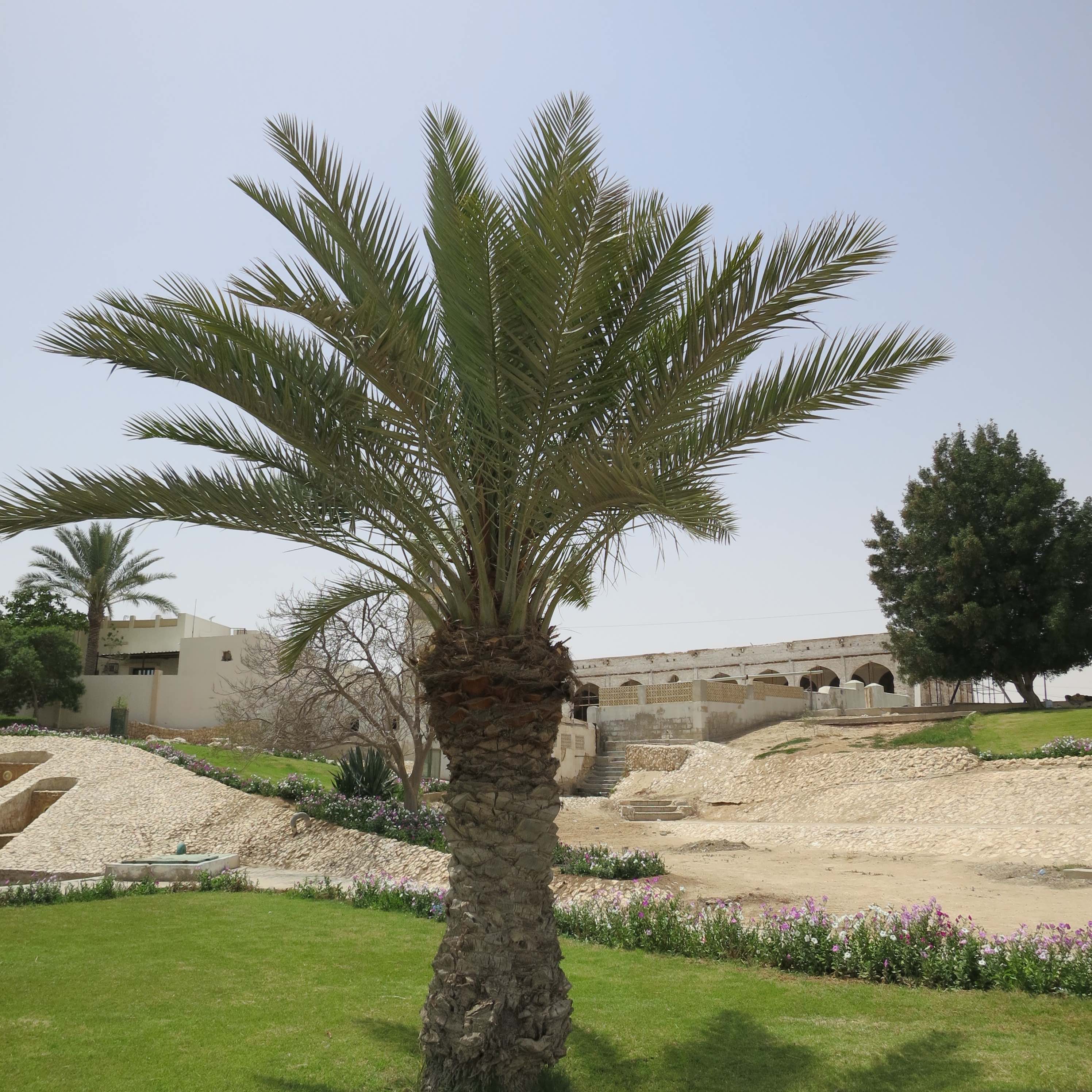

Supplement: S2 File — The images depict morphological characteristics of date palm trees growing in the State of Qatar. (ZIP) [file pone.0207299.s002.zip › Additional_Dataset_2_reduced/004 B.jpg]

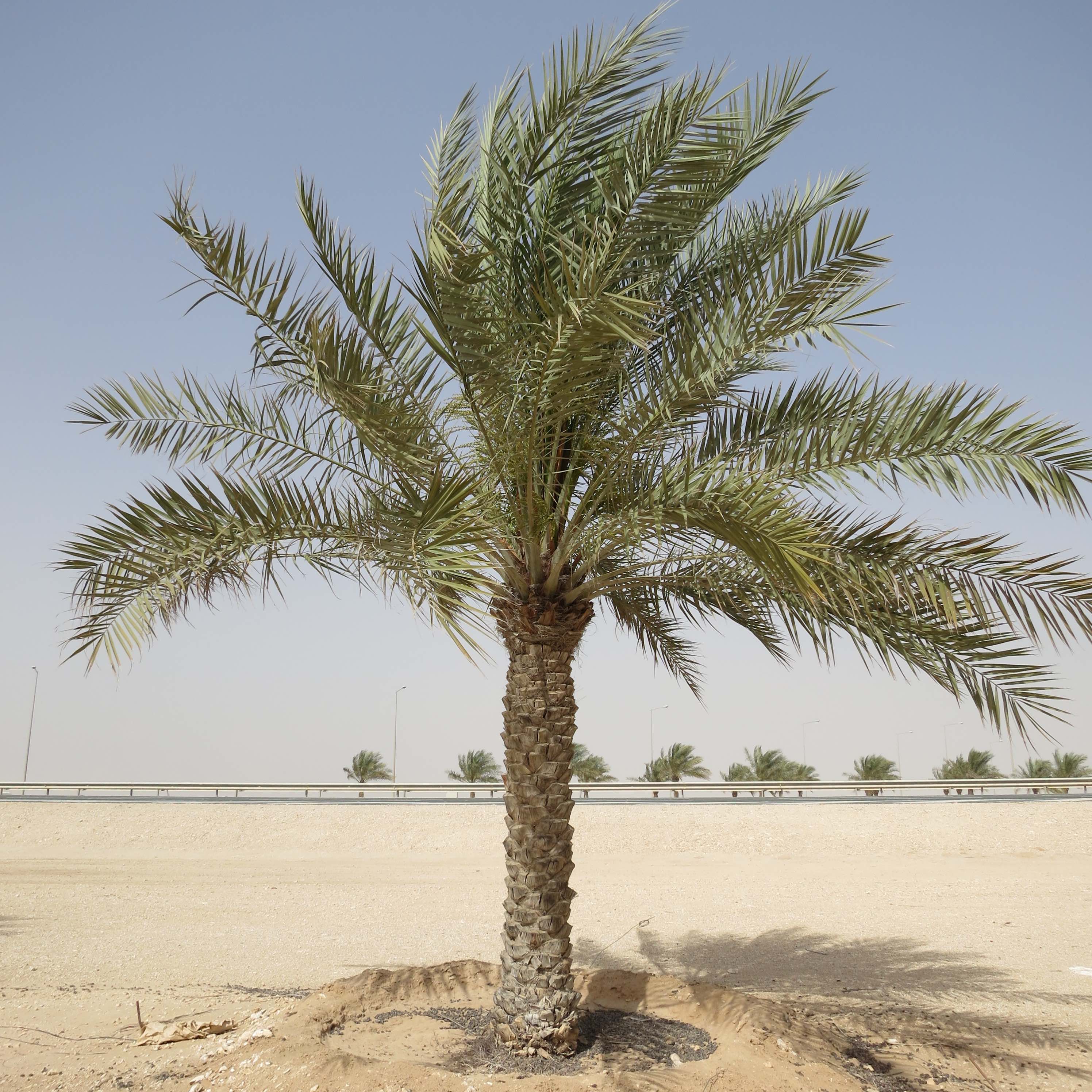

Supplement: S2 File — The images depict morphological characteristics of date palm trees growing in the State of Qatar. (ZIP) [file pone.0207299.s002.zip › Additional_Dataset_2_reduced/002 D.jpg]

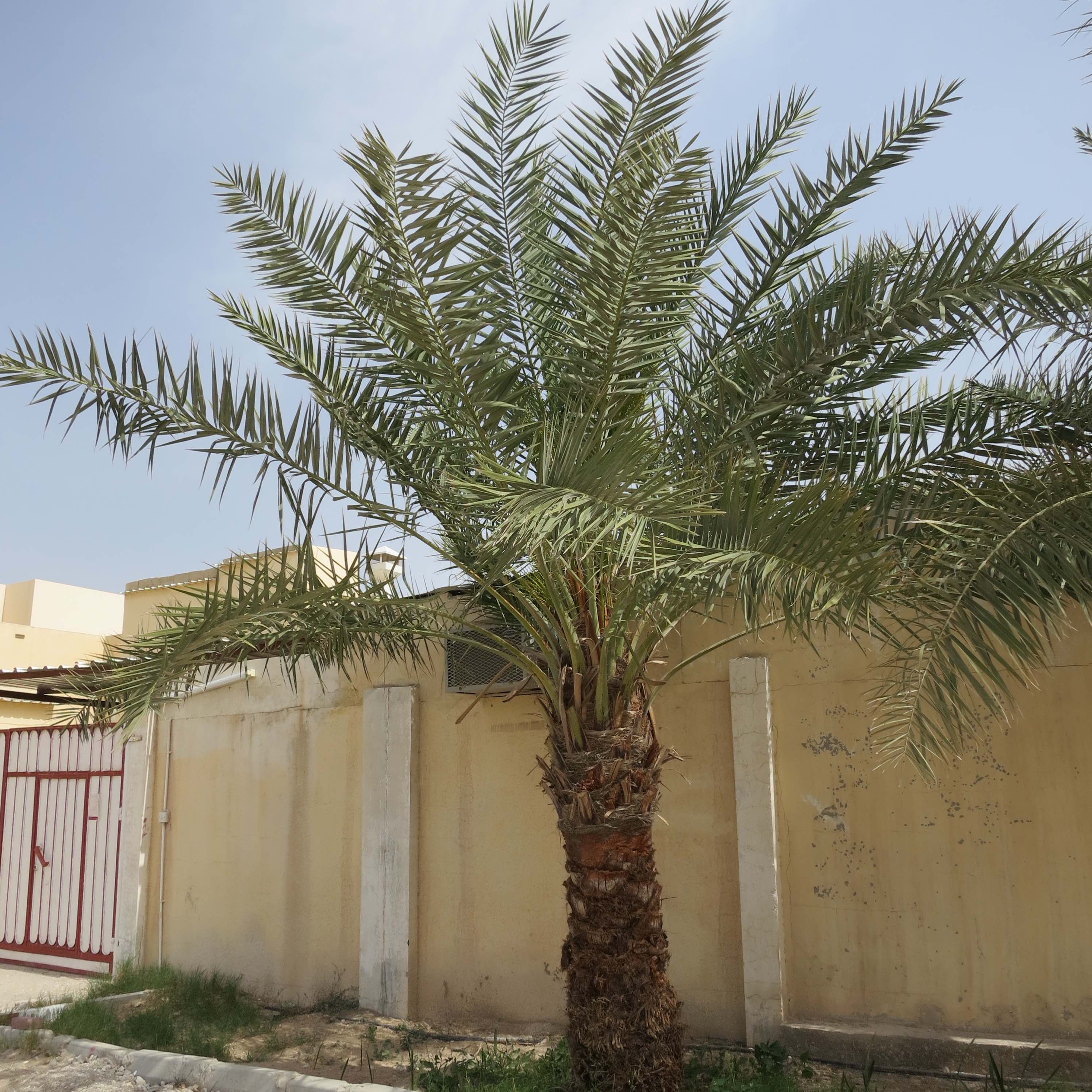

Supplement: S2 File — The images depict morphological characteristics of date palm trees growing in the State of Qatar. (ZIP) [file pone.0207299.s002.zip › Additional_Dataset_2_reduced/002 E.jpg]

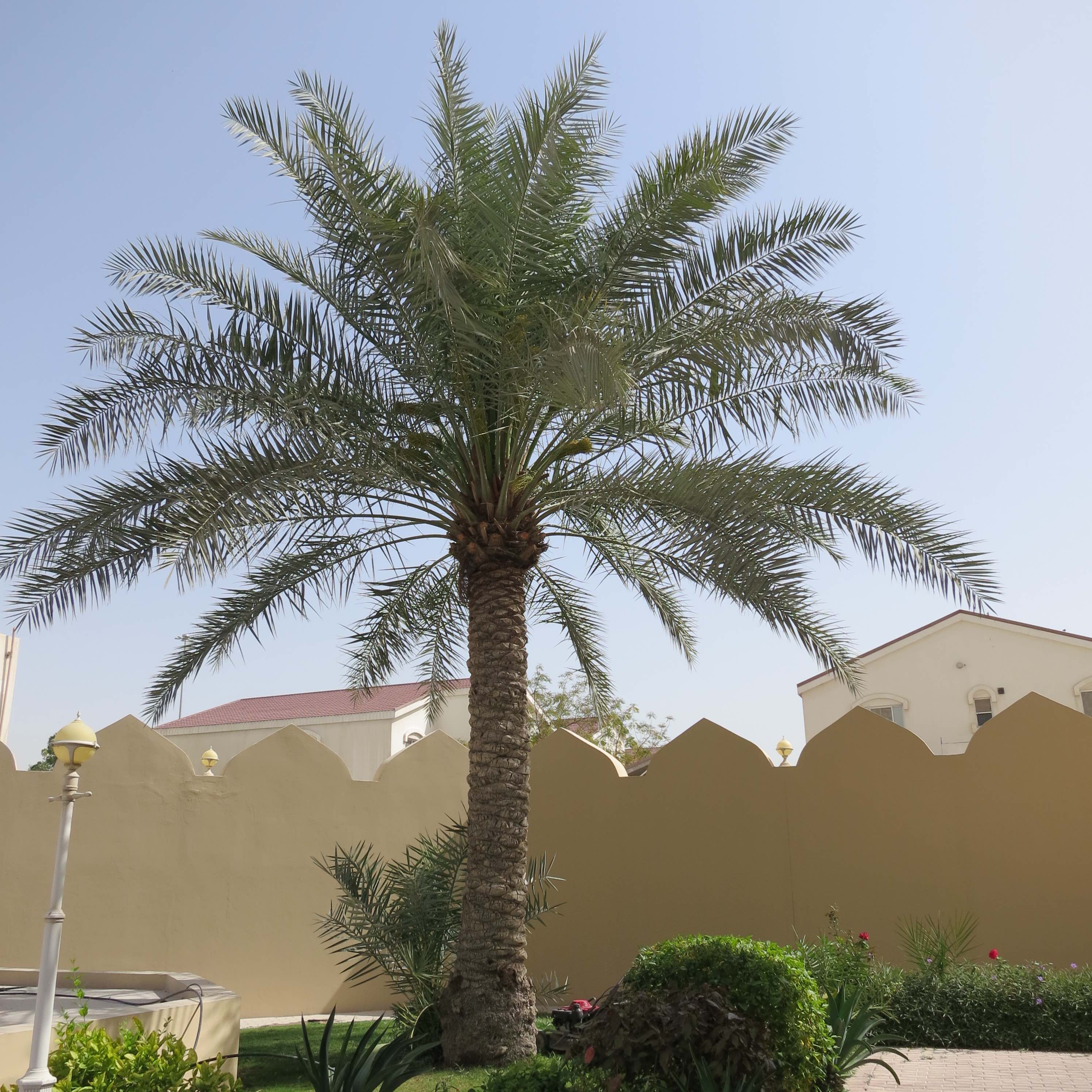

Supplement: S2 File — The images depict morphological characteristics of date palm trees growing in the State of Qatar. (ZIP) [file pone.0207299.s002.zip › Additional_Dataset_2_reduced/006 A.jpg]

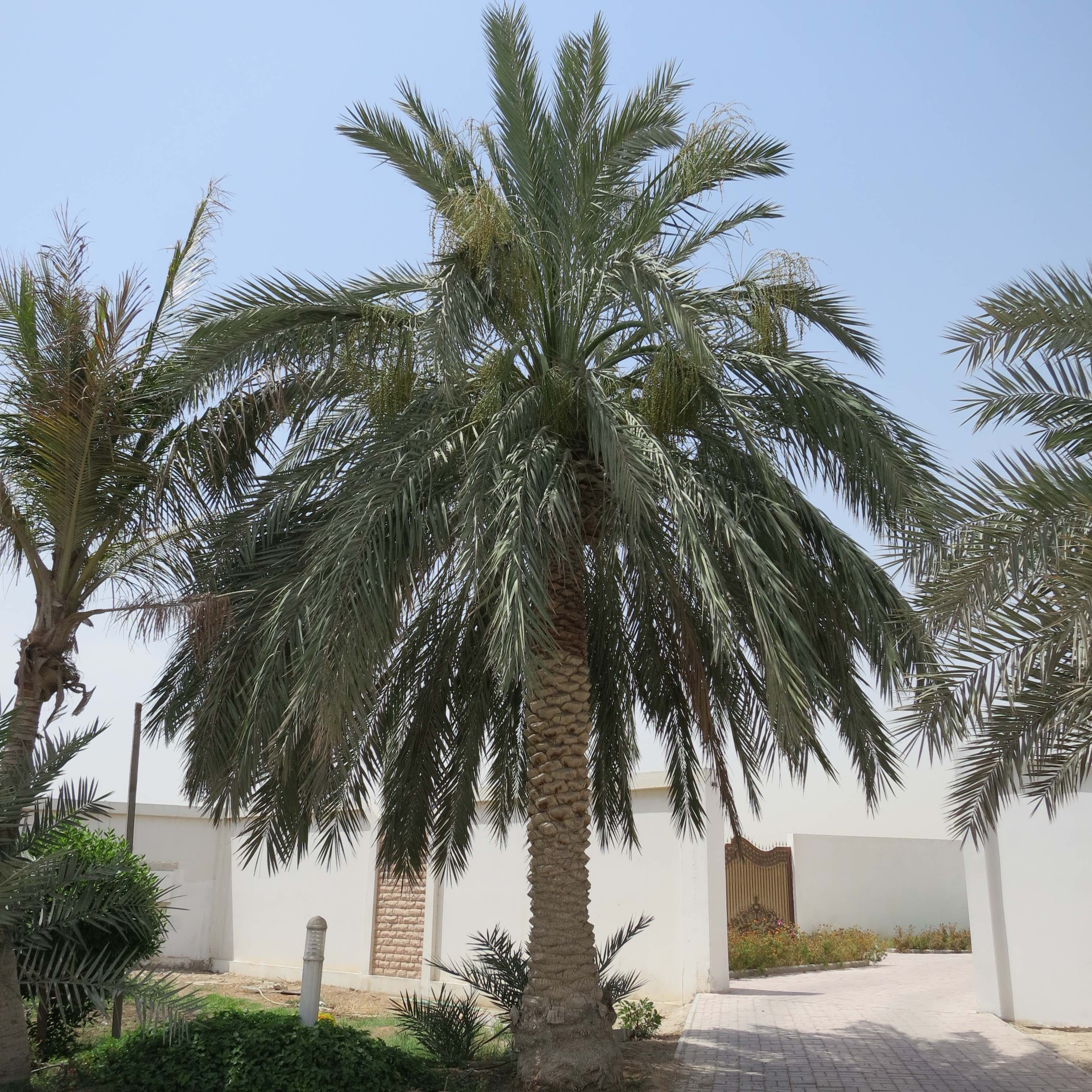

Supplement: S2 File — The images depict morphological characteristics of date palm trees growing in the State of Qatar. (ZIP) [file pone.0207299.s002.zip › Additional_Dataset_2_reduced/004 G.jpg]

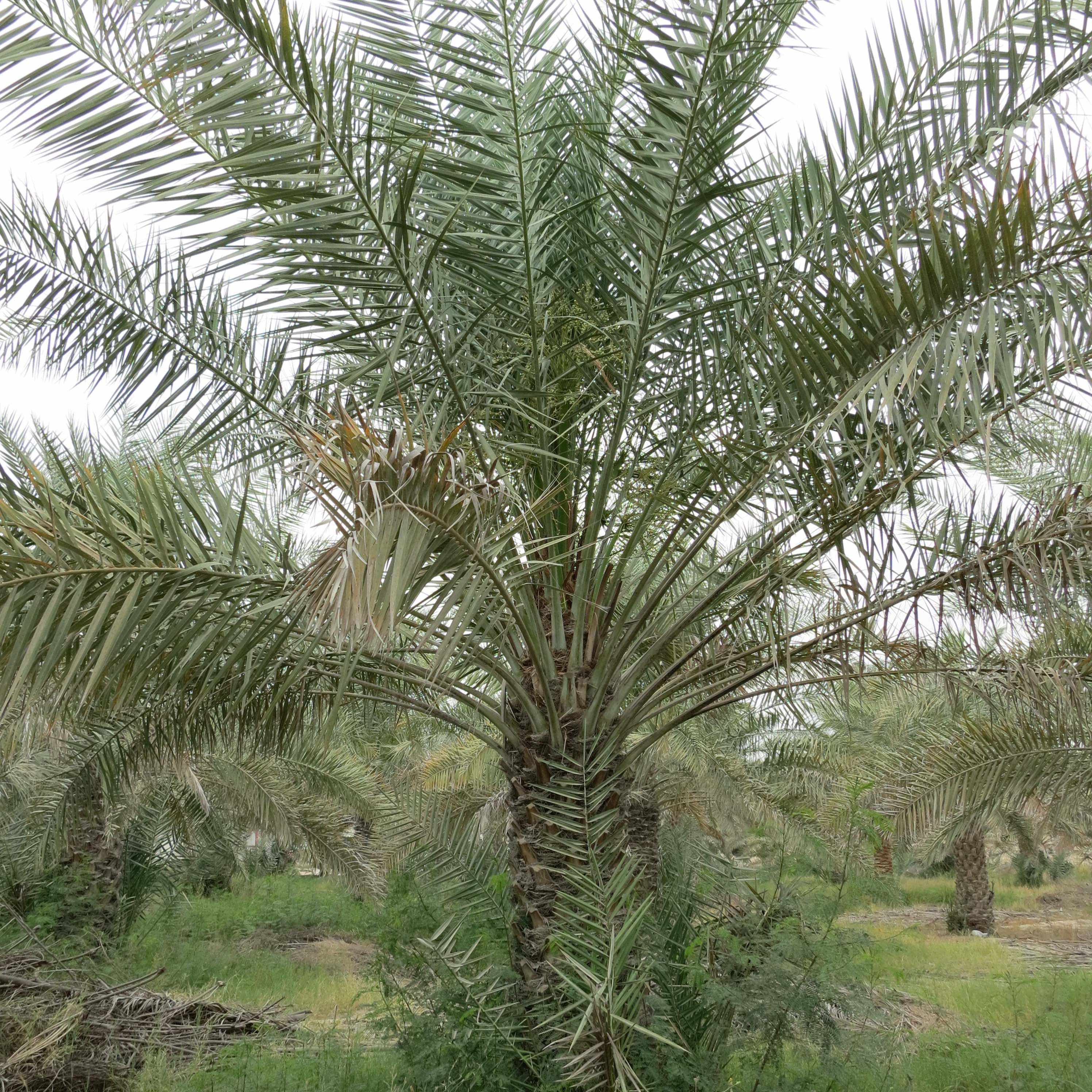

Supplement: S2 File — The images depict morphological characteristics of date palm trees growing in the State of Qatar. (ZIP) [file pone.0207299.s002.zip › Additional_Dataset_2_reduced/020 G.jpg]

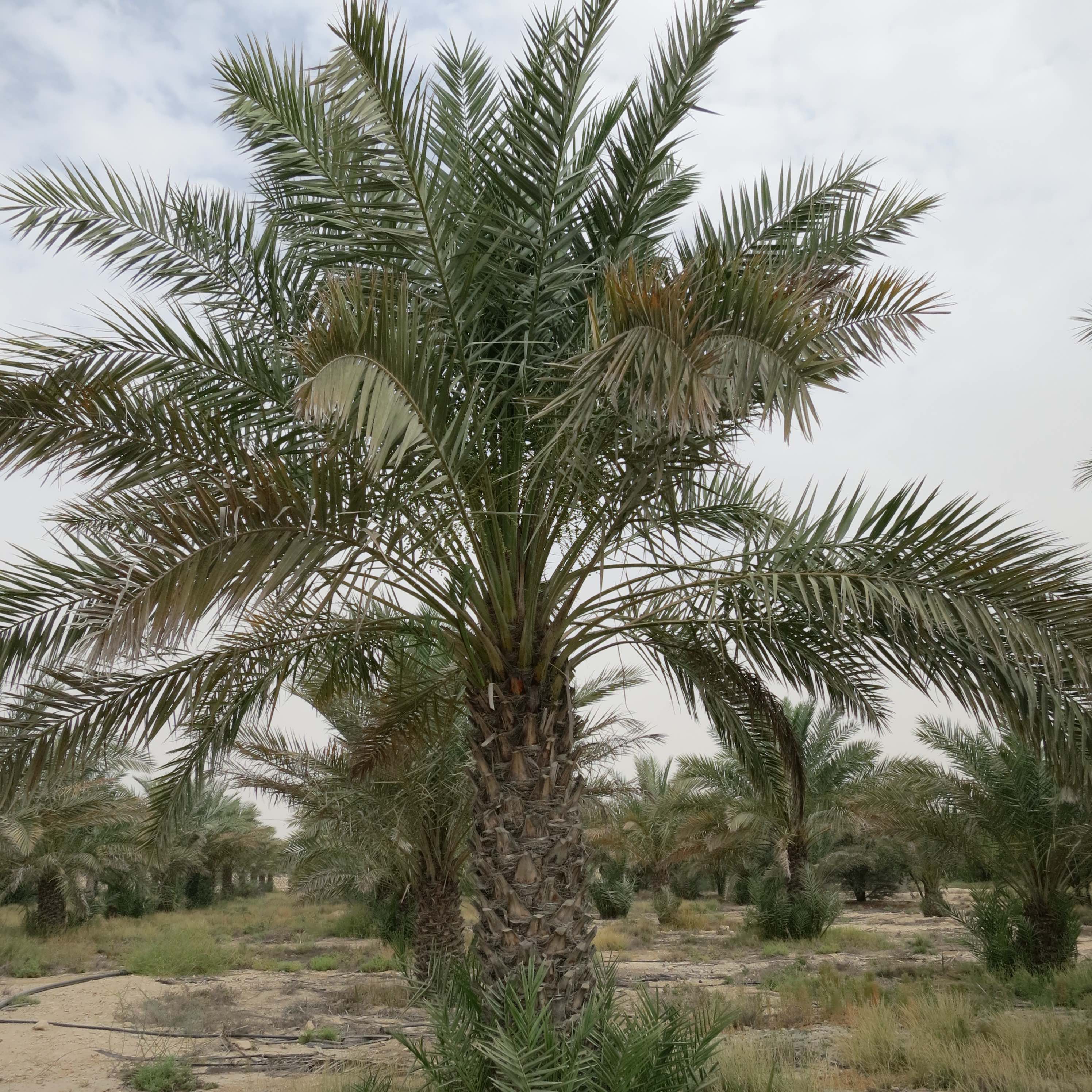

Supplement: S2 File — The images depict morphological characteristics of date palm trees growing in the State of Qatar. (ZIP) [file pone.0207299.s002.zip › Additional_Dataset_2_reduced/022 G.jpg]

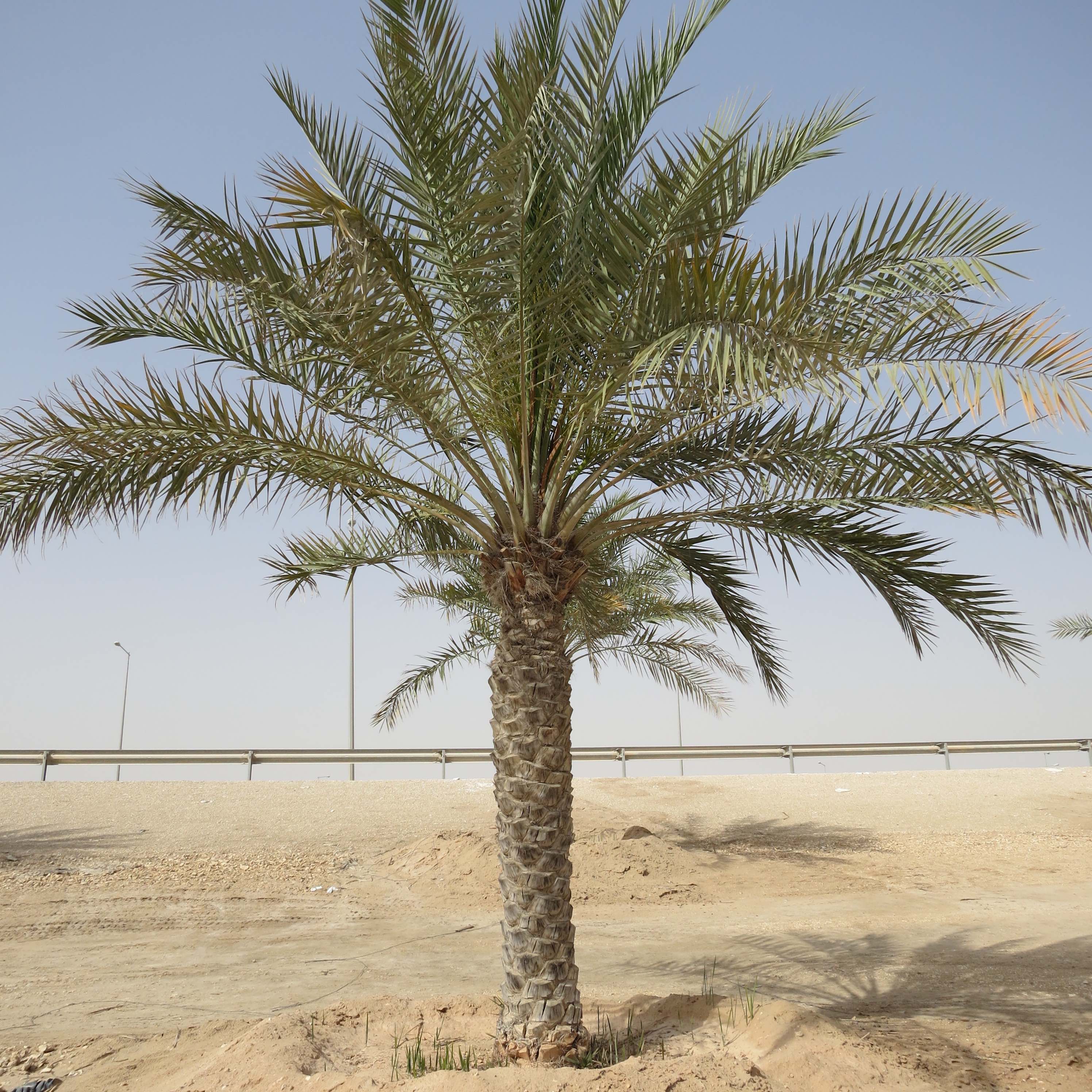

Supplement: S2 File — The images depict morphological characteristics of date palm trees growing in the State of Qatar. (ZIP) [file pone.0207299.s002.zip › Additional_Dataset_2_reduced/004 D.jpg]

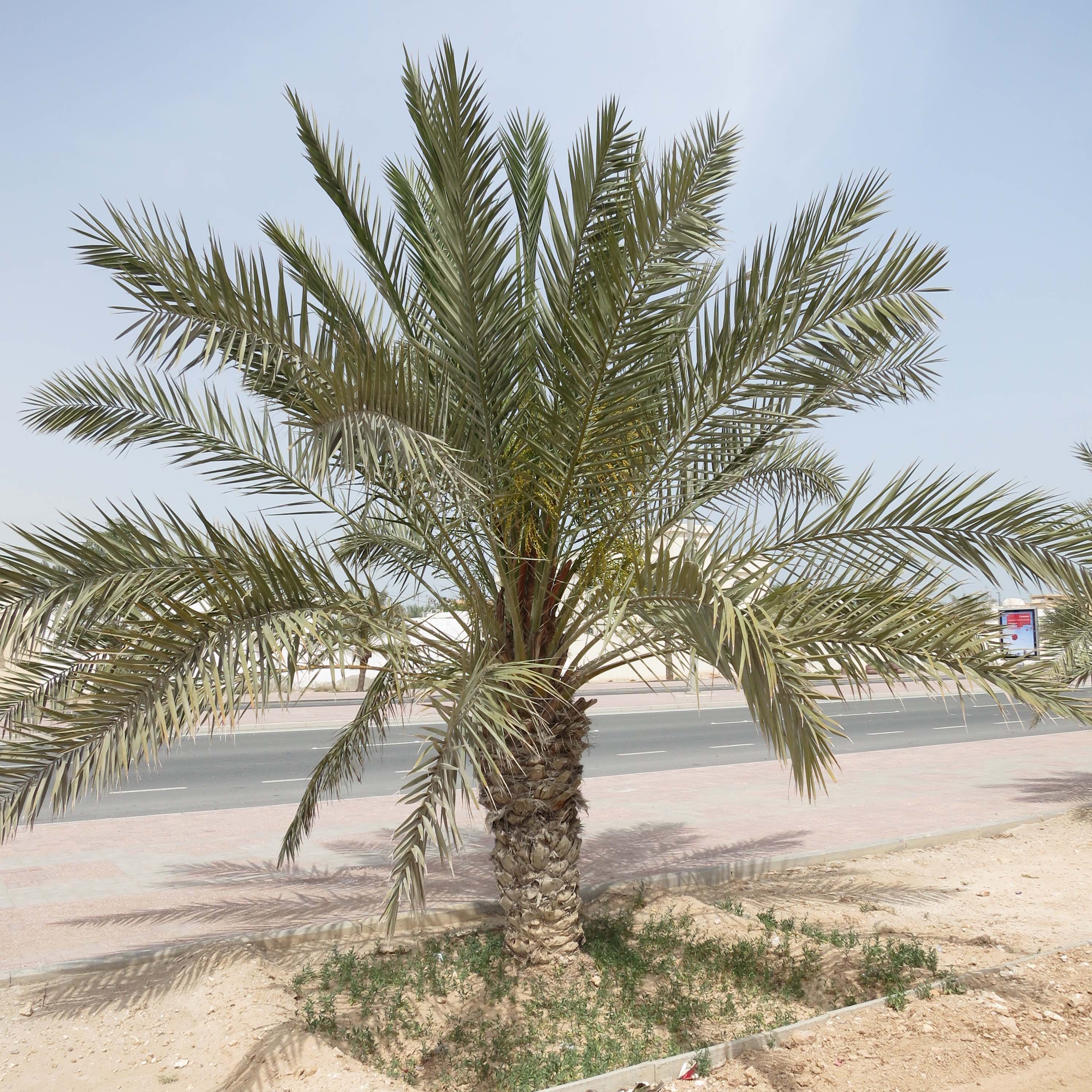

Supplement: S2 File — The images depict morphological characteristics of date palm trees growing in the State of Qatar. (ZIP) [file pone.0207299.s002.zip › Additional_Dataset_2_reduced/006 F.jpg]

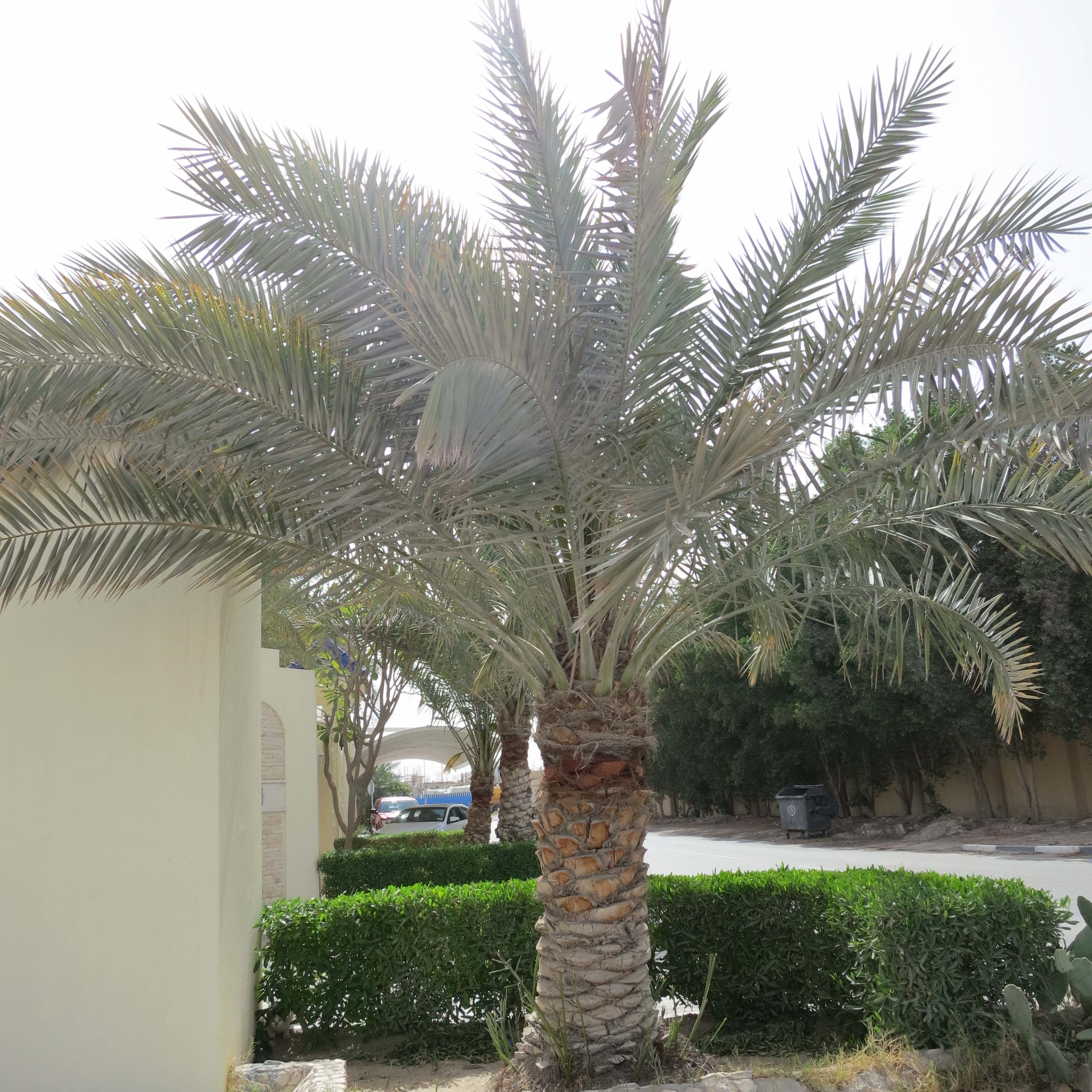

Supplement: S2 File — The images depict morphological characteristics of date palm trees growing in the State of Qatar. (ZIP) [file pone.0207299.s002.zip › Additional_Dataset_2_reduced/004 E.jpg]

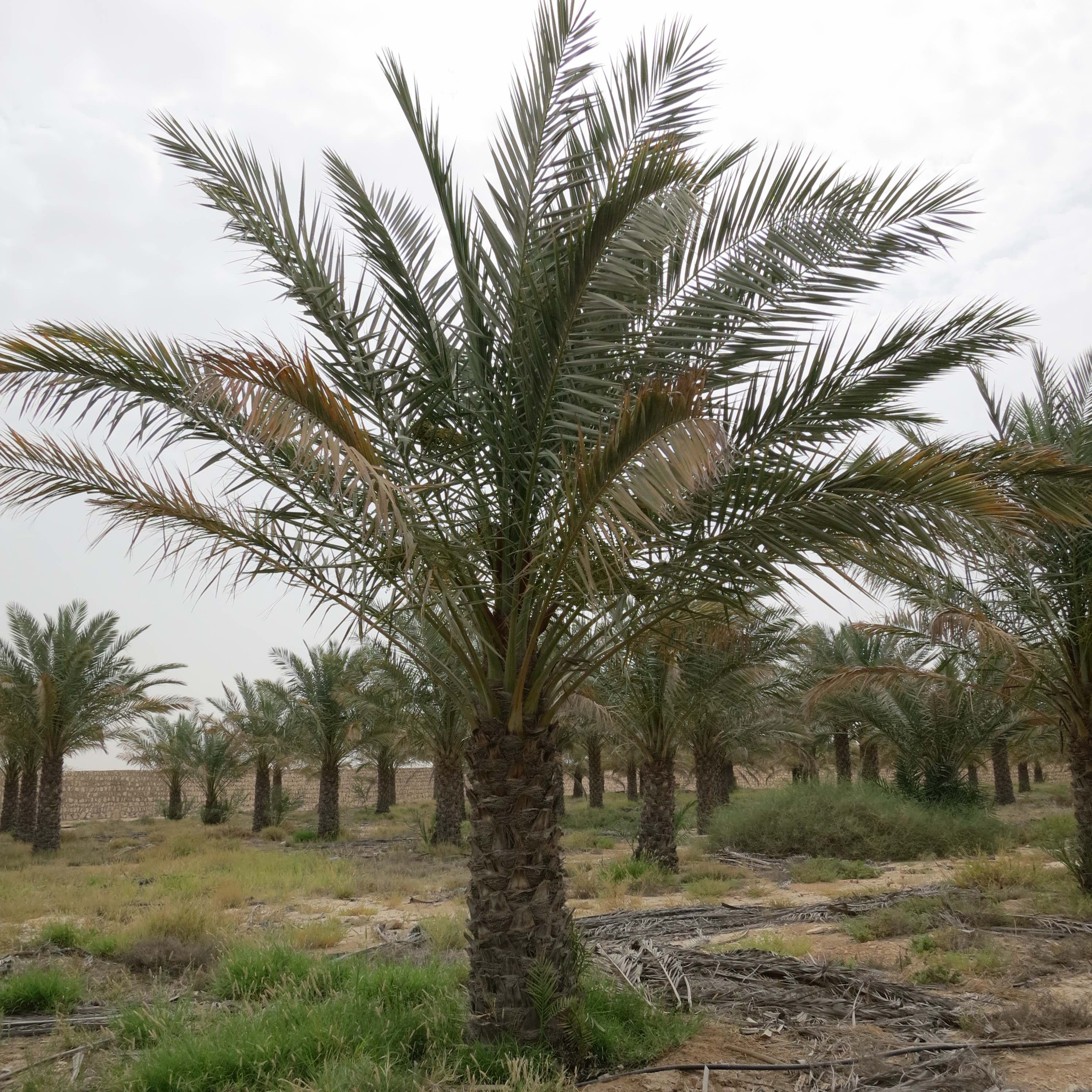

Supplement: S2 File — The images depict morphological characteristics of date palm trees growing in the State of Qatar. (ZIP) [file pone.0207299.s002.zip › Additional_Dataset_2_reduced/025 G.jpg]

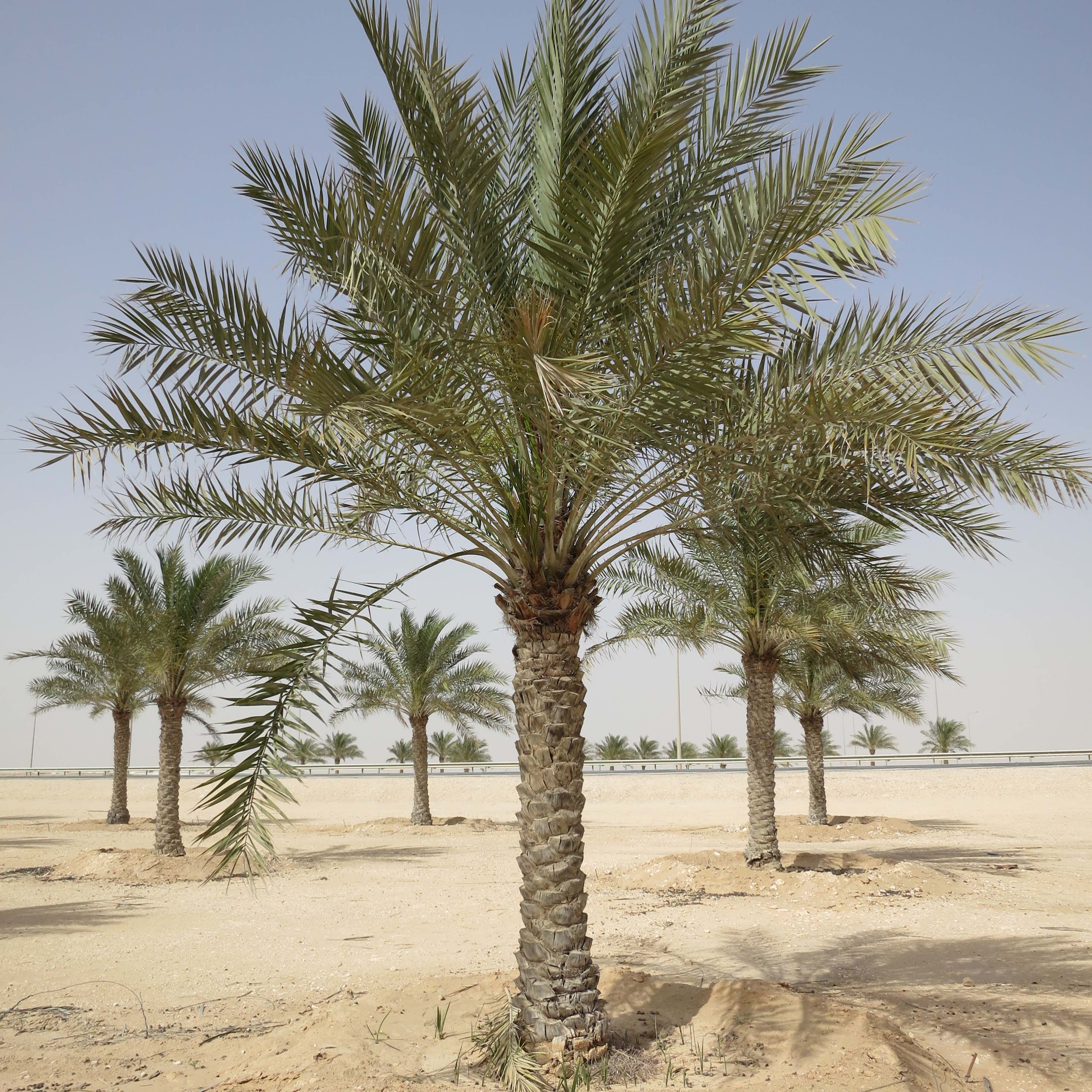

Supplement: S2 File — The images depict morphological characteristics of date palm trees growing in the State of Qatar. (ZIP) [file pone.0207299.s002.zip › Additional_Dataset_2_reduced/003 D.jpg]

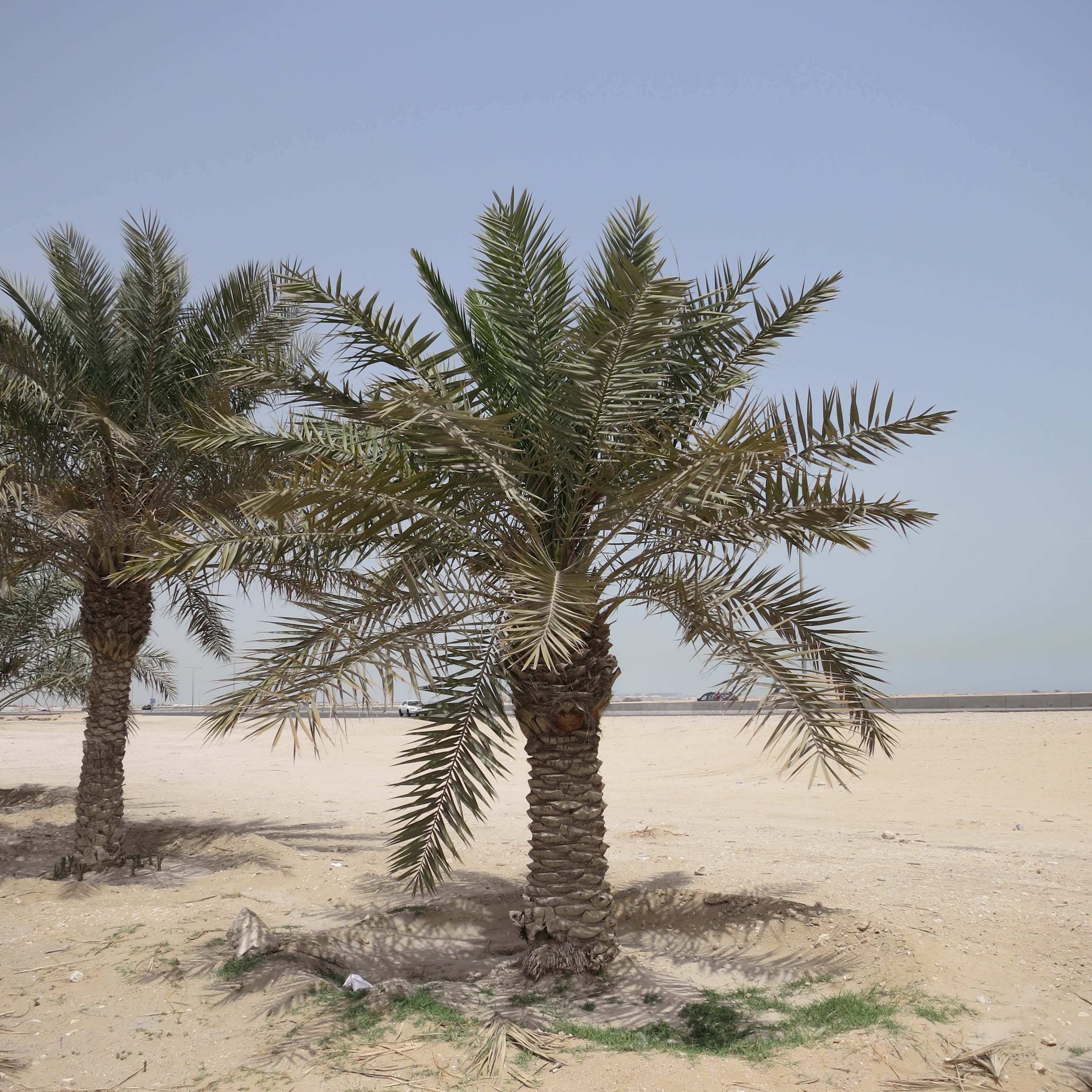

Supplement: S2 File — The images depict morphological characteristics of date palm trees growing in the State of Qatar. (ZIP) [file pone.0207299.s002.zip › Additional_Dataset_2_reduced/001 F.jpg]

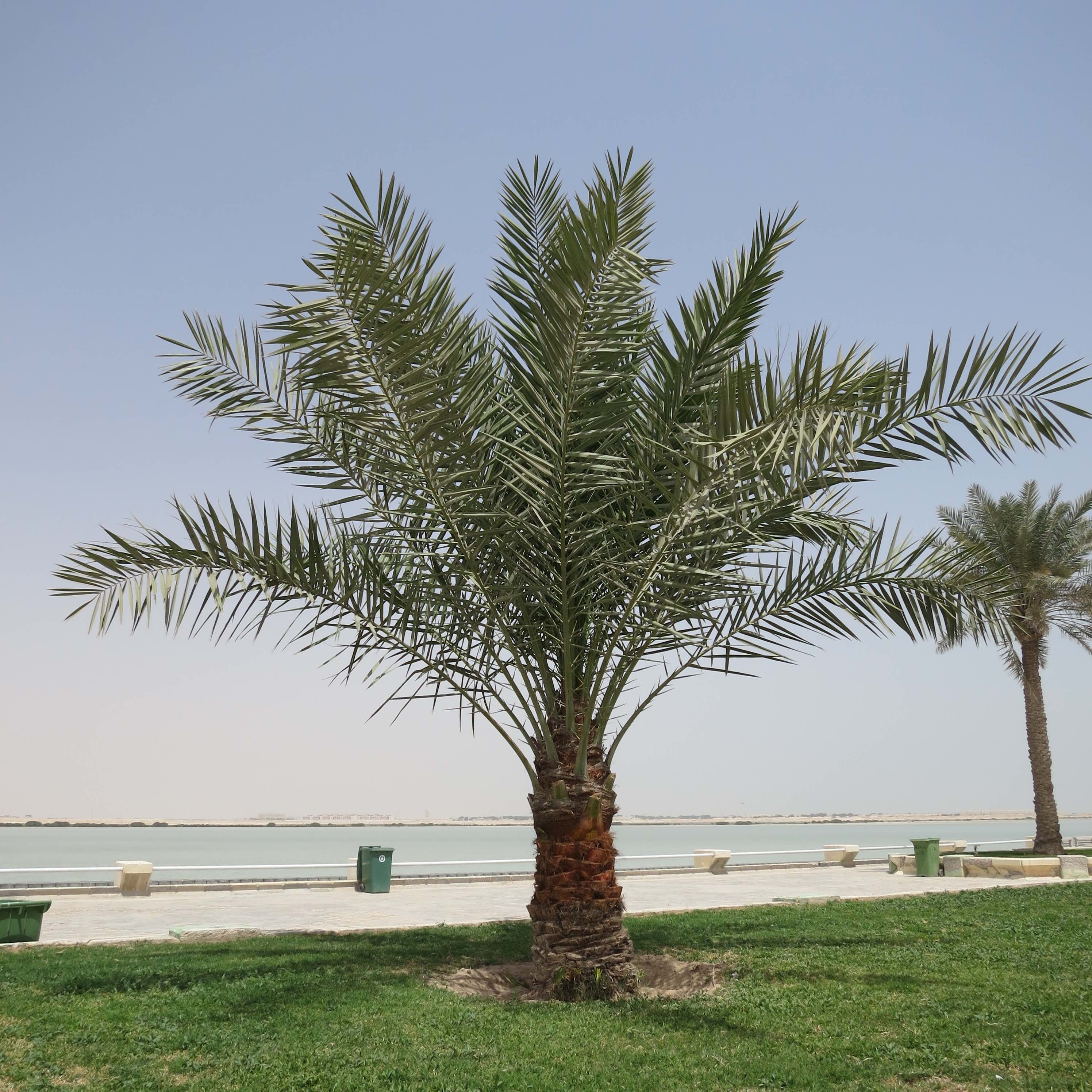

Supplement: S2 File — The images depict morphological characteristics of date palm trees growing in the State of Qatar. (ZIP) [file pone.0207299.s002.zip › Additional_Dataset_2_reduced/005 B.jpg]

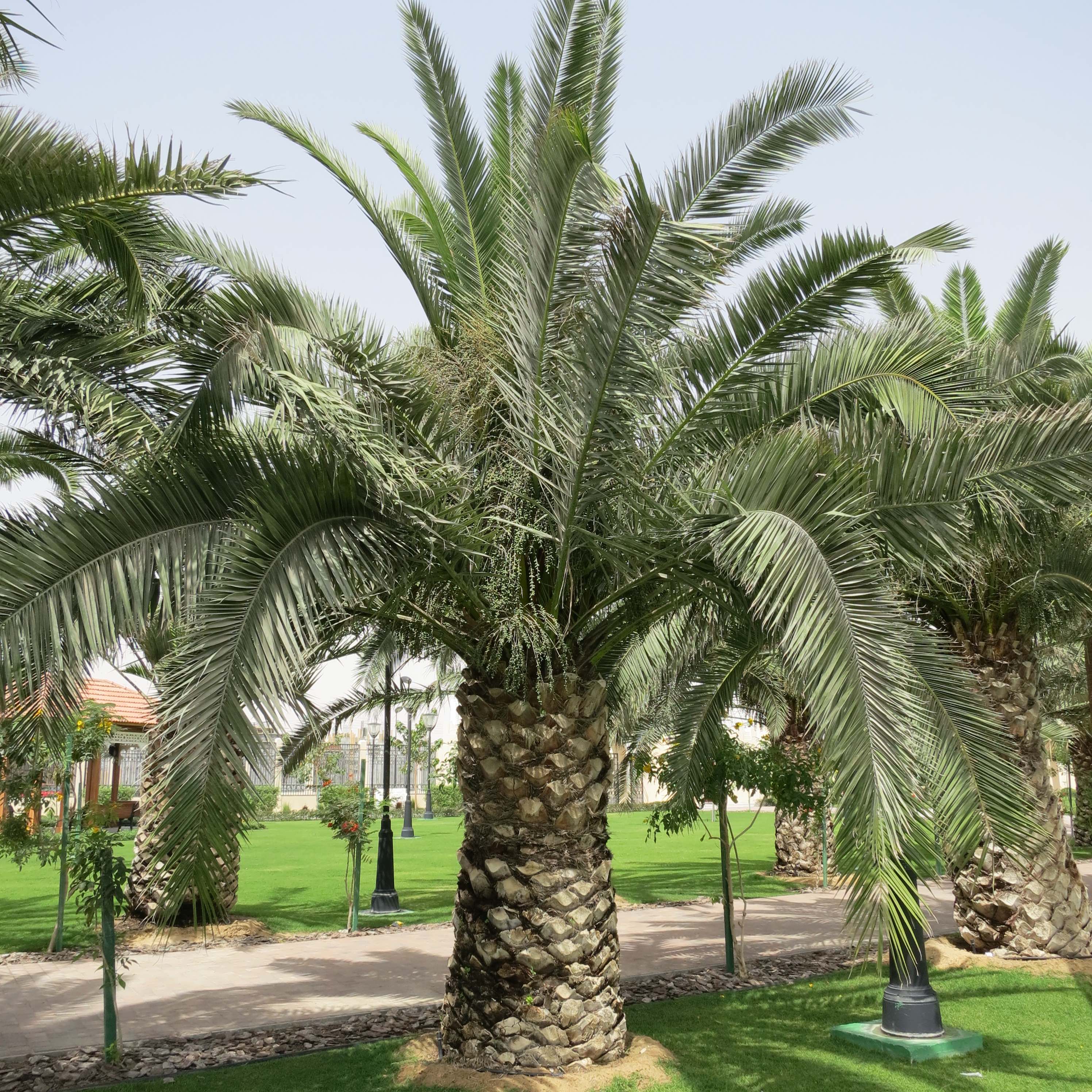

Supplement: S2 File — The images depict morphological characteristics of date palm trees growing in the State of Qatar. (ZIP) [file pone.0207299.s002.zip › Additional_Dataset_2_reduced/005 C.jpg]

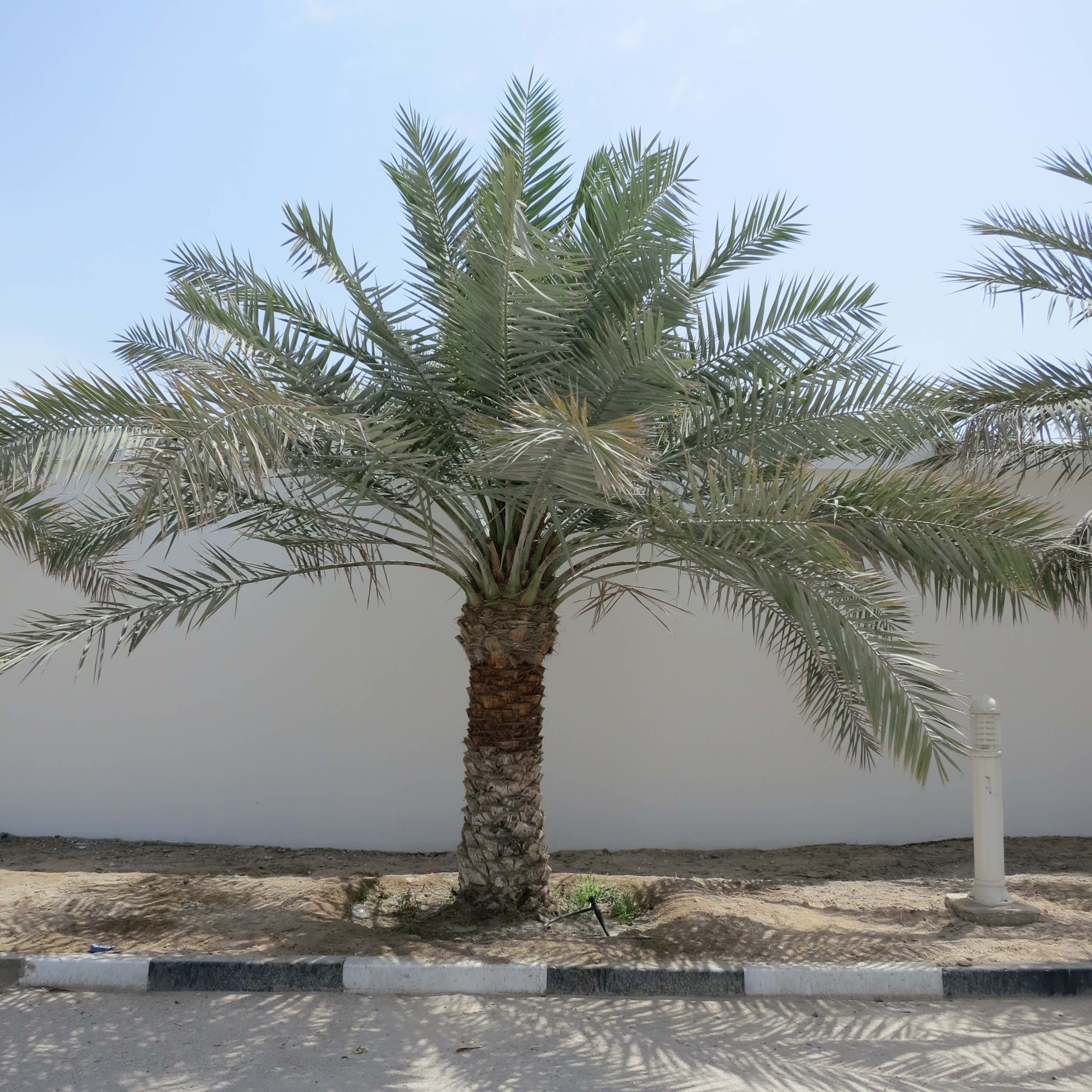

Supplement: S2 File — The images depict morphological characteristics of date palm trees growing in the State of Qatar. (ZIP) [file pone.0207299.s002.zip › Additional_Dataset_2_reduced/001 G.jpg]

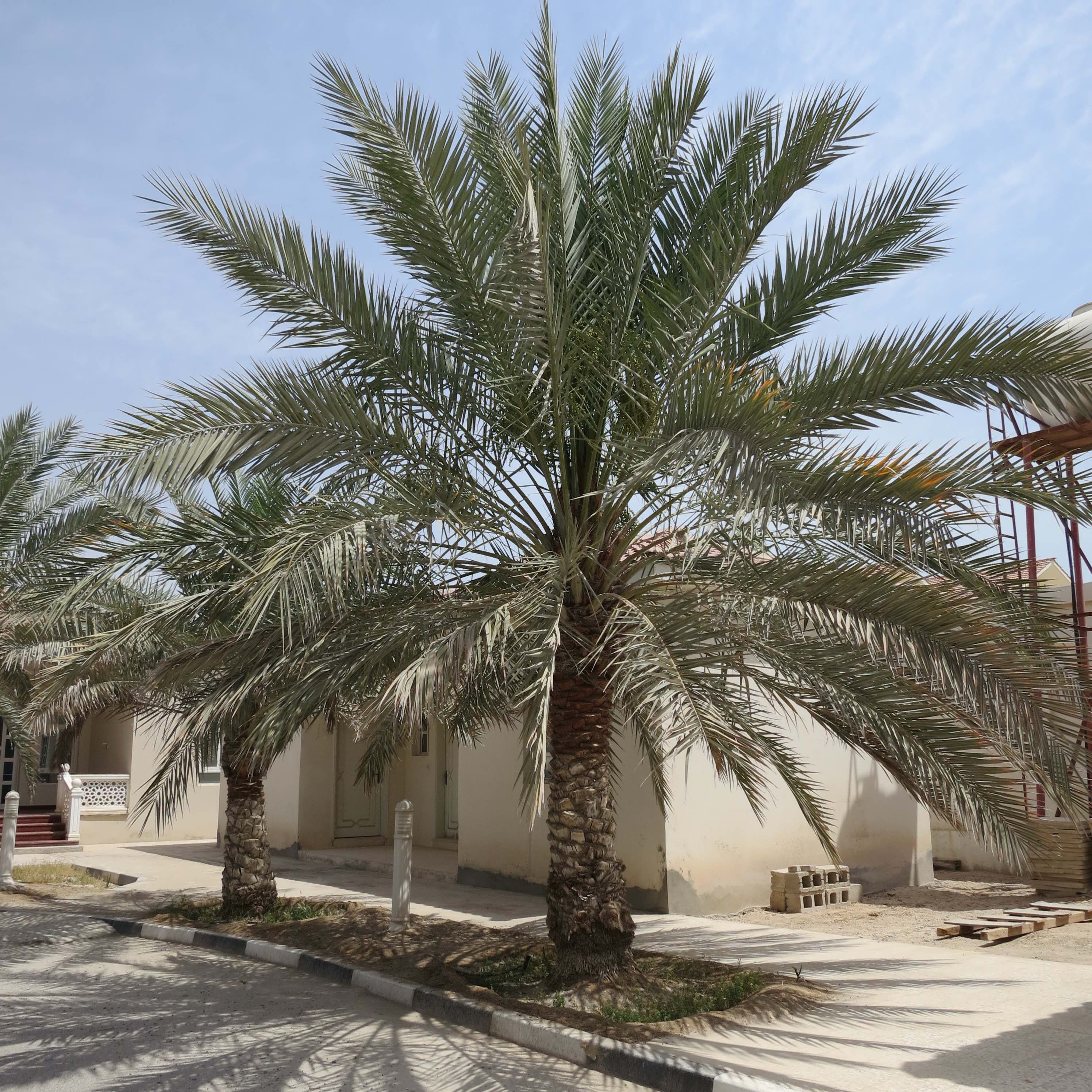

Supplement: S2 File — The images depict morphological characteristics of date palm trees growing in the State of Qatar. (ZIP) [file pone.0207299.s002.zip › Additional_Dataset_2_reduced/003 G.jpg]

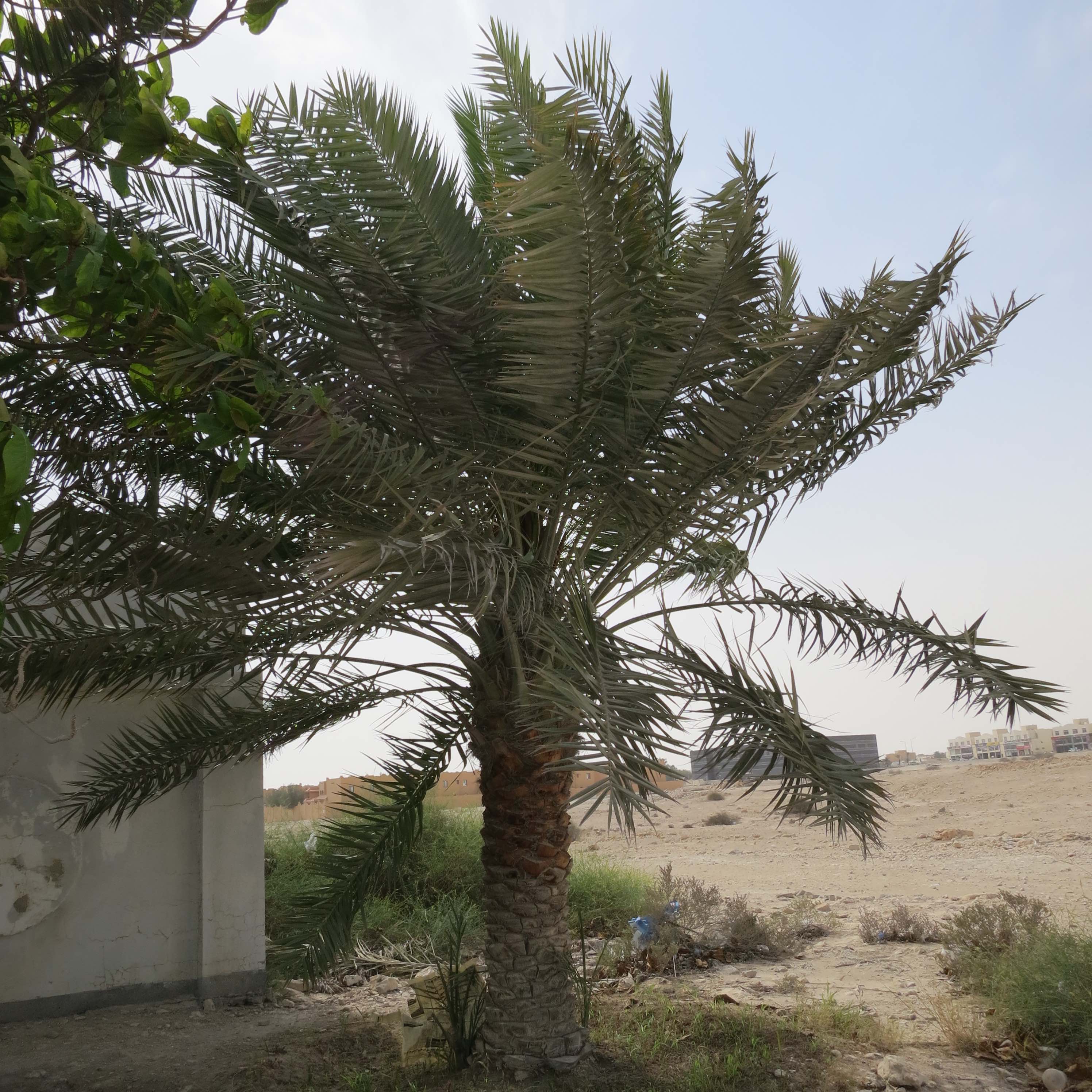

Supplement: S2 File — The images depict morphological characteristics of date palm trees growing in the State of Qatar. (ZIP) [file pone.0207299.s002.zip › Additional_Dataset_2_reduced/001 E.jpg]

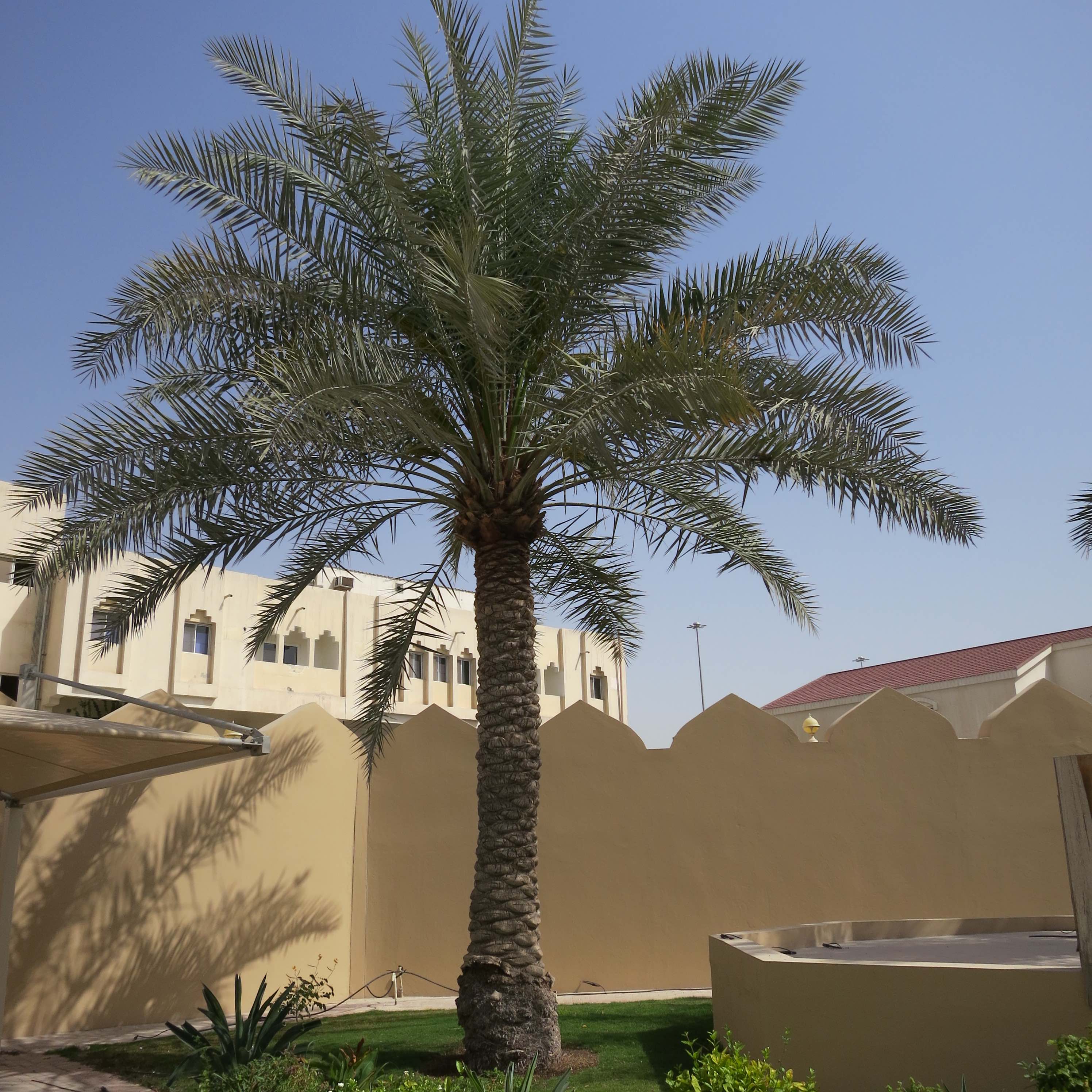

Supplement: S2 File — The images depict morphological characteristics of date palm trees growing in the State of Qatar. (ZIP) [file pone.0207299.s002.zip › Additional_Dataset_2_reduced/005 A.jpg]

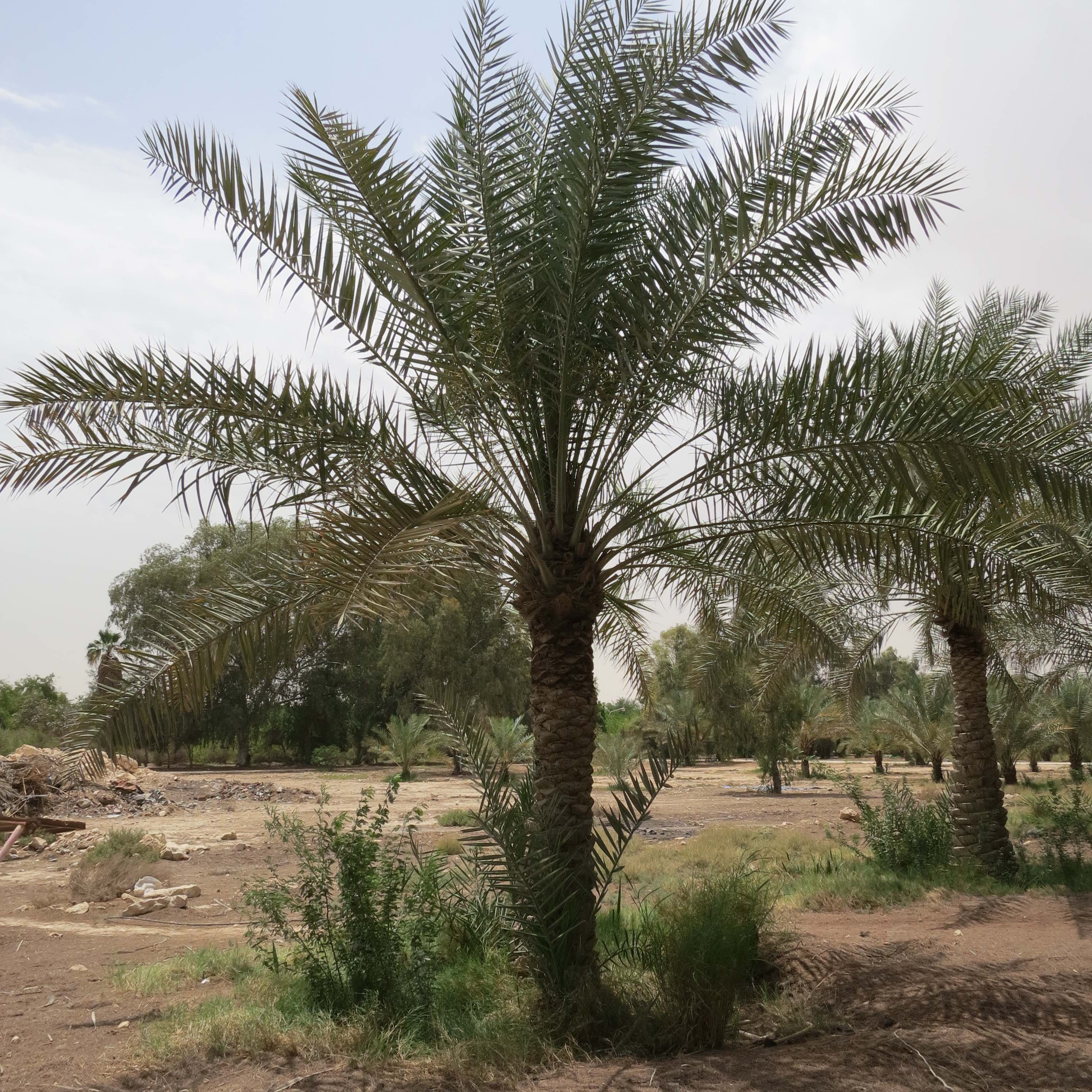

Supplement: S2 File — The images depict morphological characteristics of date palm trees growing in the State of Qatar. (ZIP) [file pone.0207299.s002.zip › Additional_Dataset_2_reduced/007 B.jpg]

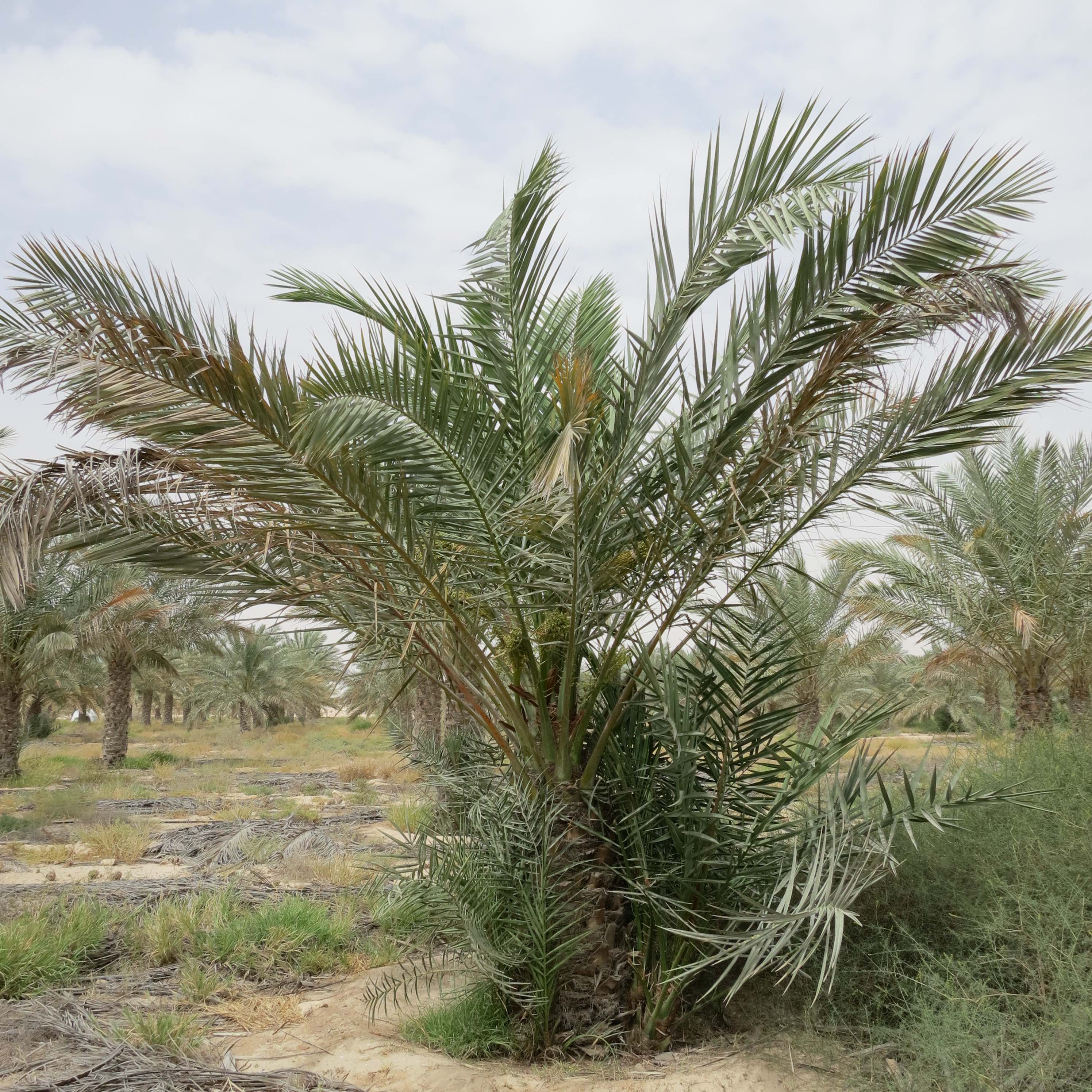

Supplement: S2 File — The images depict morphological characteristics of date palm trees growing in the State of Qatar. (ZIP) [file pone.0207299.s002.zip › Additional_Dataset_2_reduced/027 G.jpg]

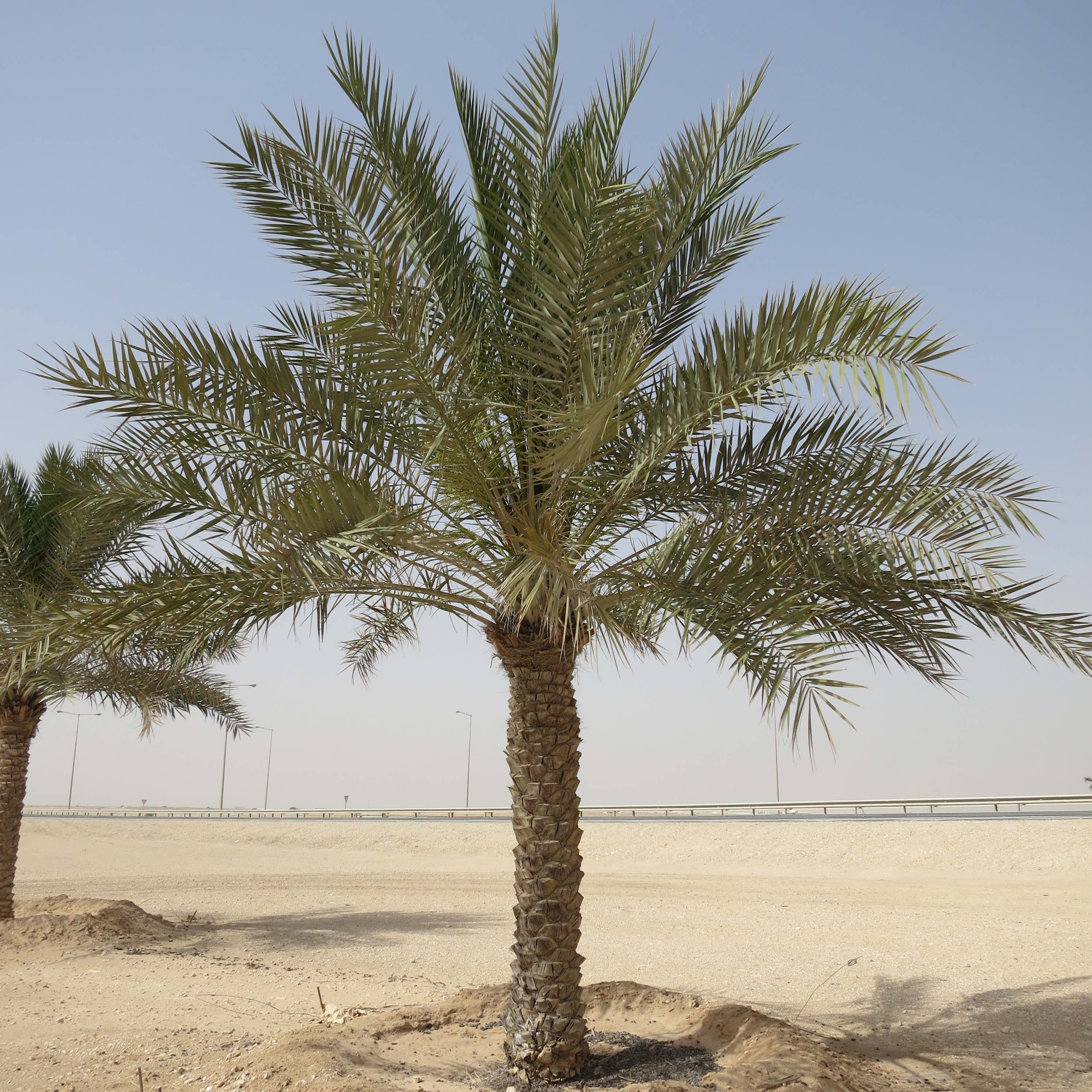

Supplement: S2 File — The images depict morphological characteristics of date palm trees growing in the State of Qatar. (ZIP) [file pone.0207299.s002.zip › Additional_Dataset_2_reduced/001 D.jpg]

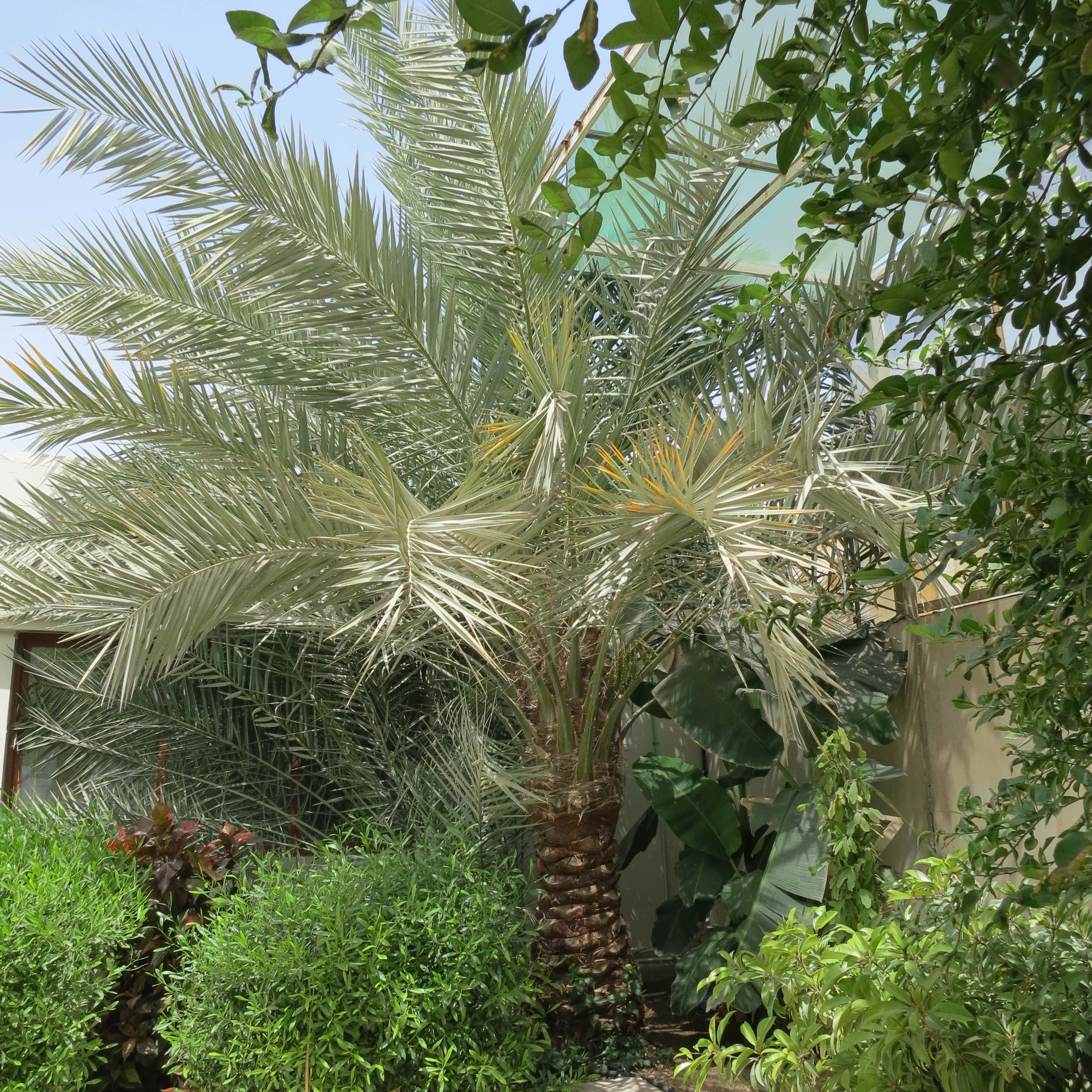

Supplement: S2 File — The images depict morphological characteristics of date palm trees growing in the State of Qatar. (ZIP) [file pone.0207299.s002.zip › Additional_Dataset_2_reduced/001 A.jpg]

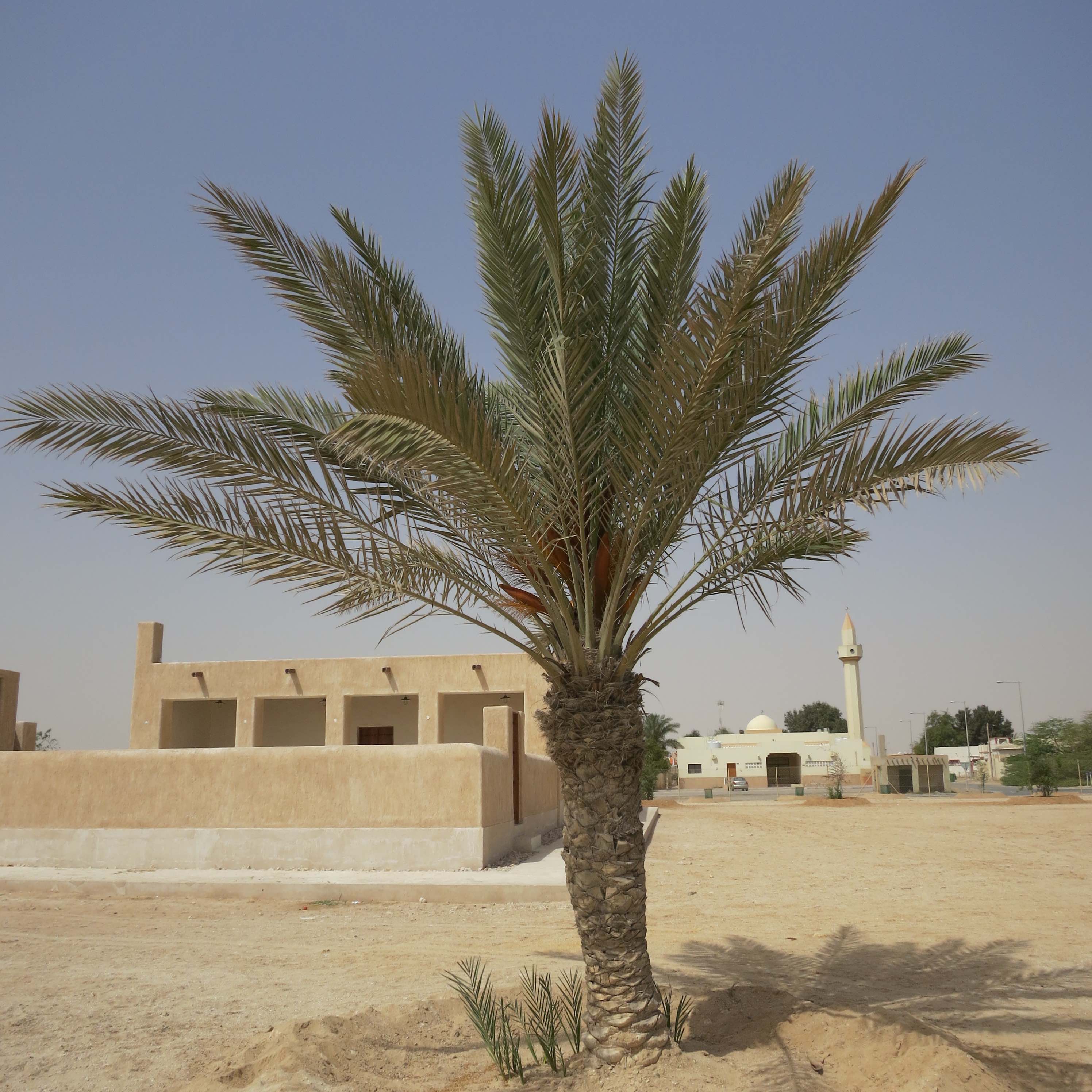

Supplement: S2 File — The images depict morphological characteristics of date palm trees growing in the State of Qatar. (ZIP) [file pone.0207299.s002.zip › Additional_Dataset_2_reduced/003 C.jpg]

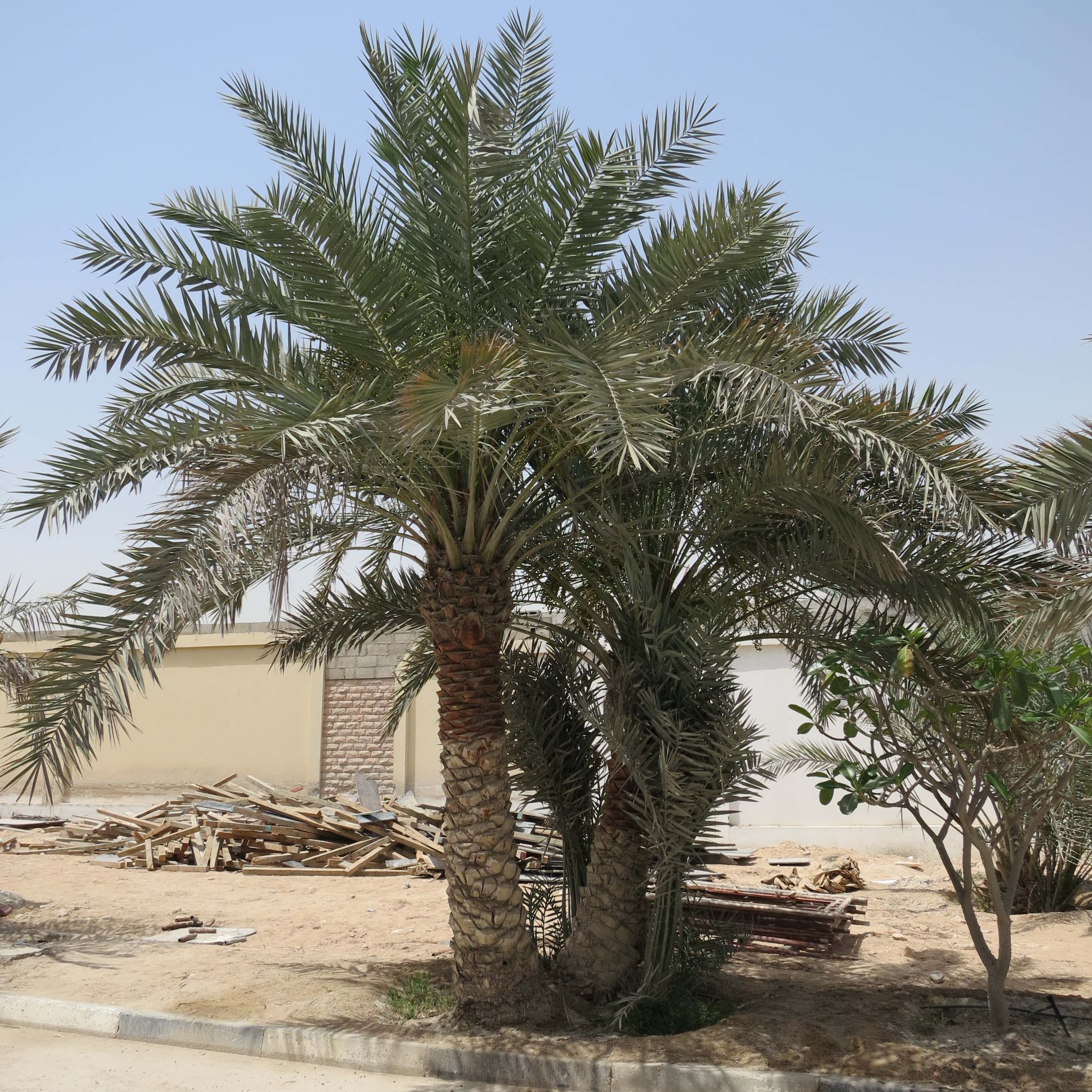

Supplement: S2 File — The images depict morphological characteristics of date palm trees growing in the State of Qatar. (ZIP) [file pone.0207299.s002.zip › Additional_Dataset_2_reduced/005 G.jpg]

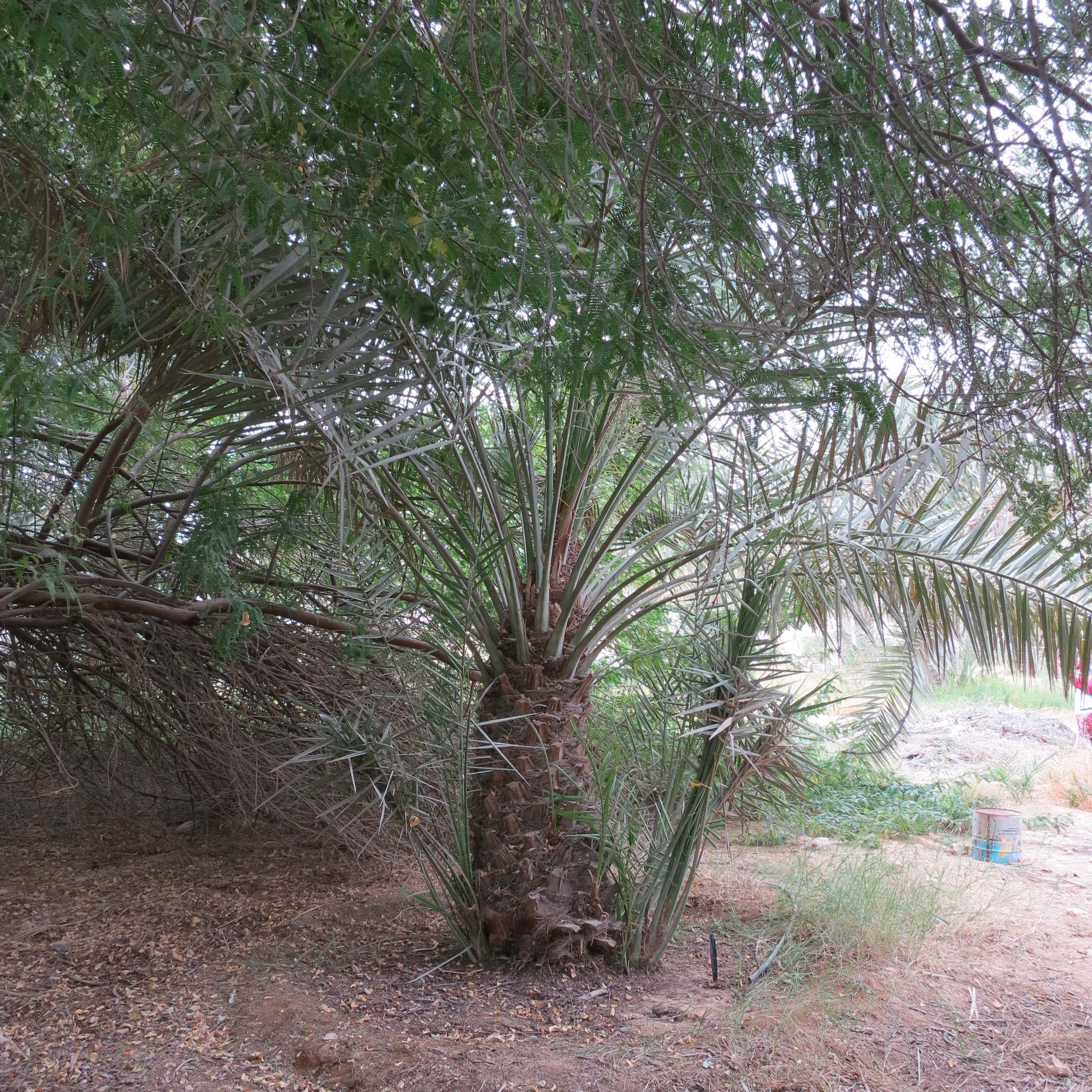

Supplement: S2 File — The images depict morphological characteristics of date palm trees growing in the State of Qatar. (ZIP) [file pone.0207299.s002.zip › Additional_Dataset_2_reduced/021 G.jpg]

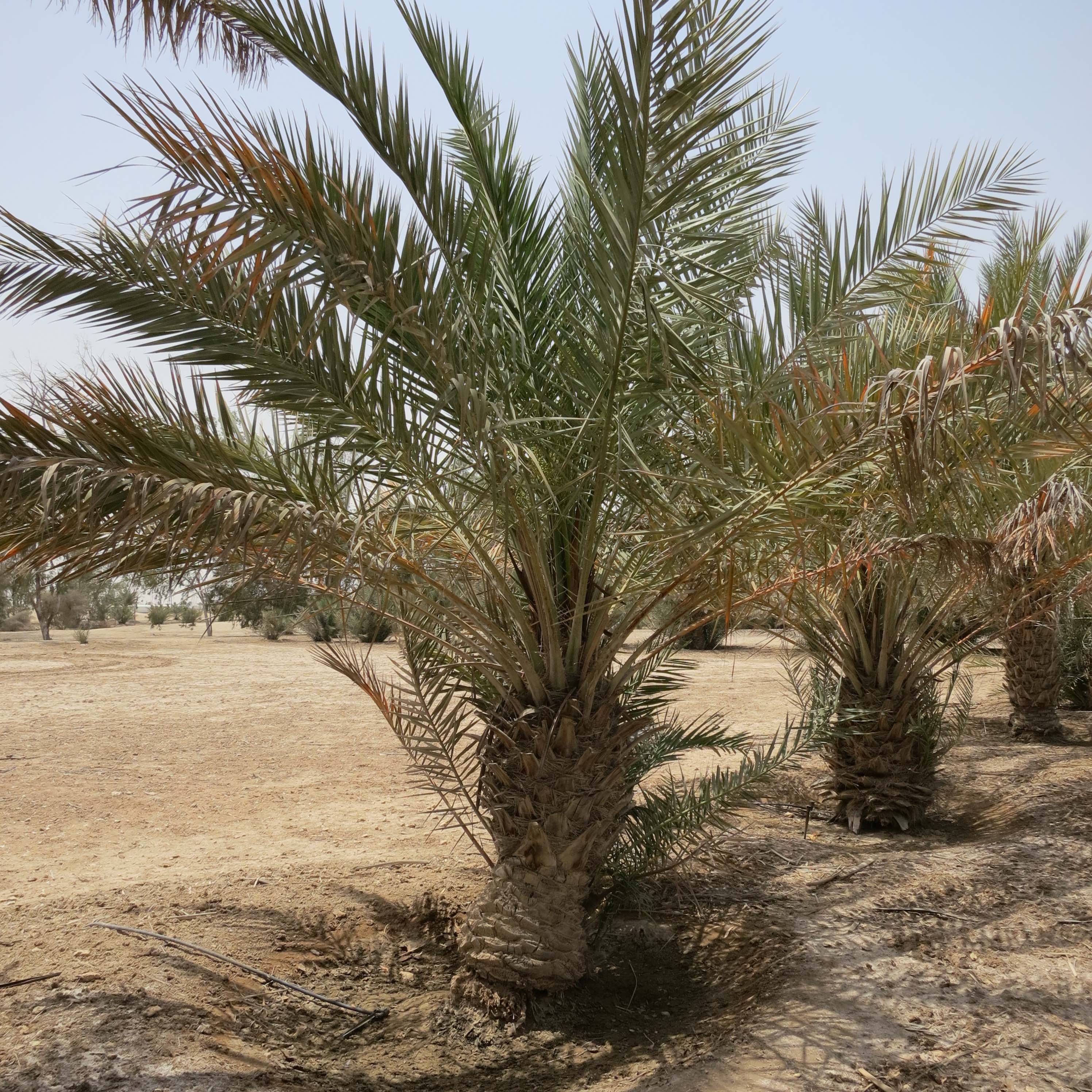

Supplement: S2 File — The images depict morphological characteristics of date palm trees growing in the State of Qatar. (ZIP) [file pone.0207299.s002.zip › Additional_Dataset_2_reduced/010 G.jpg]

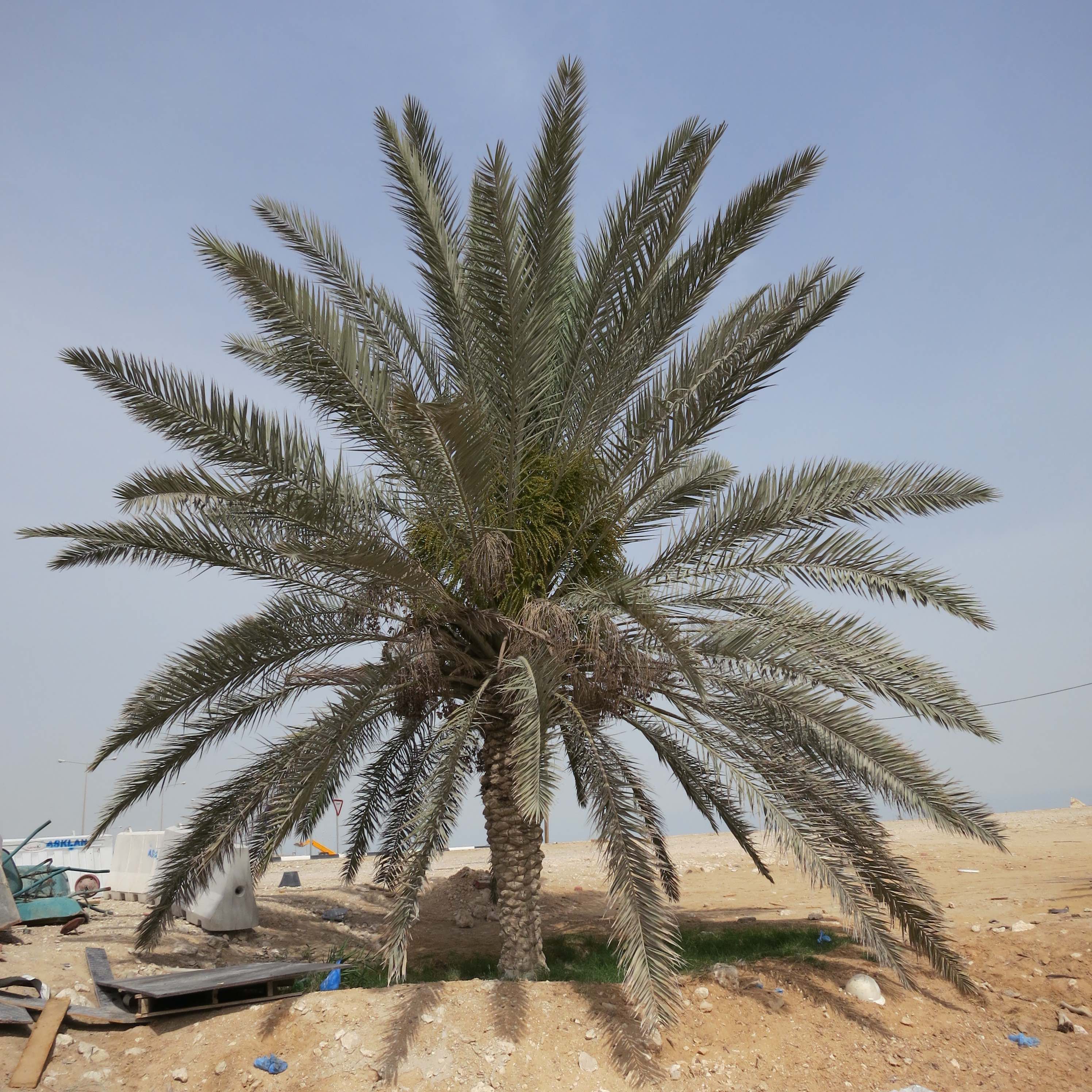

Supplement: S2 File — The images depict morphological characteristics of date palm trees growing in the State of Qatar. (ZIP) [file pone.0207299.s002.zip › Additional_Dataset_2_reduced/009 F.jpg]

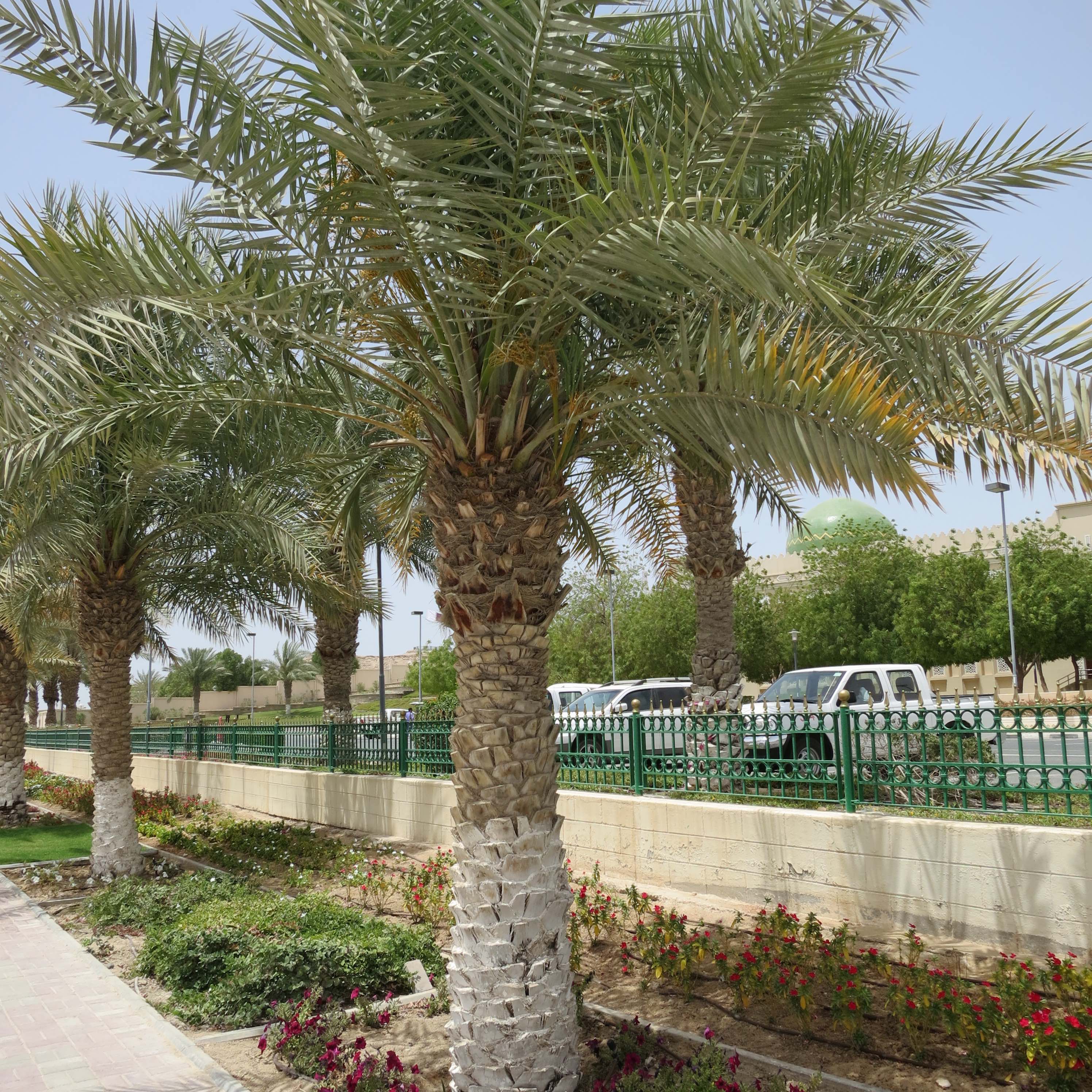

Supplement: S2 File — The images depict morphological characteristics of date palm trees growing in the State of Qatar. (ZIP) [file pone.0207299.s002.zip › Additional_Dataset_2_reduced/034 G.jpg]

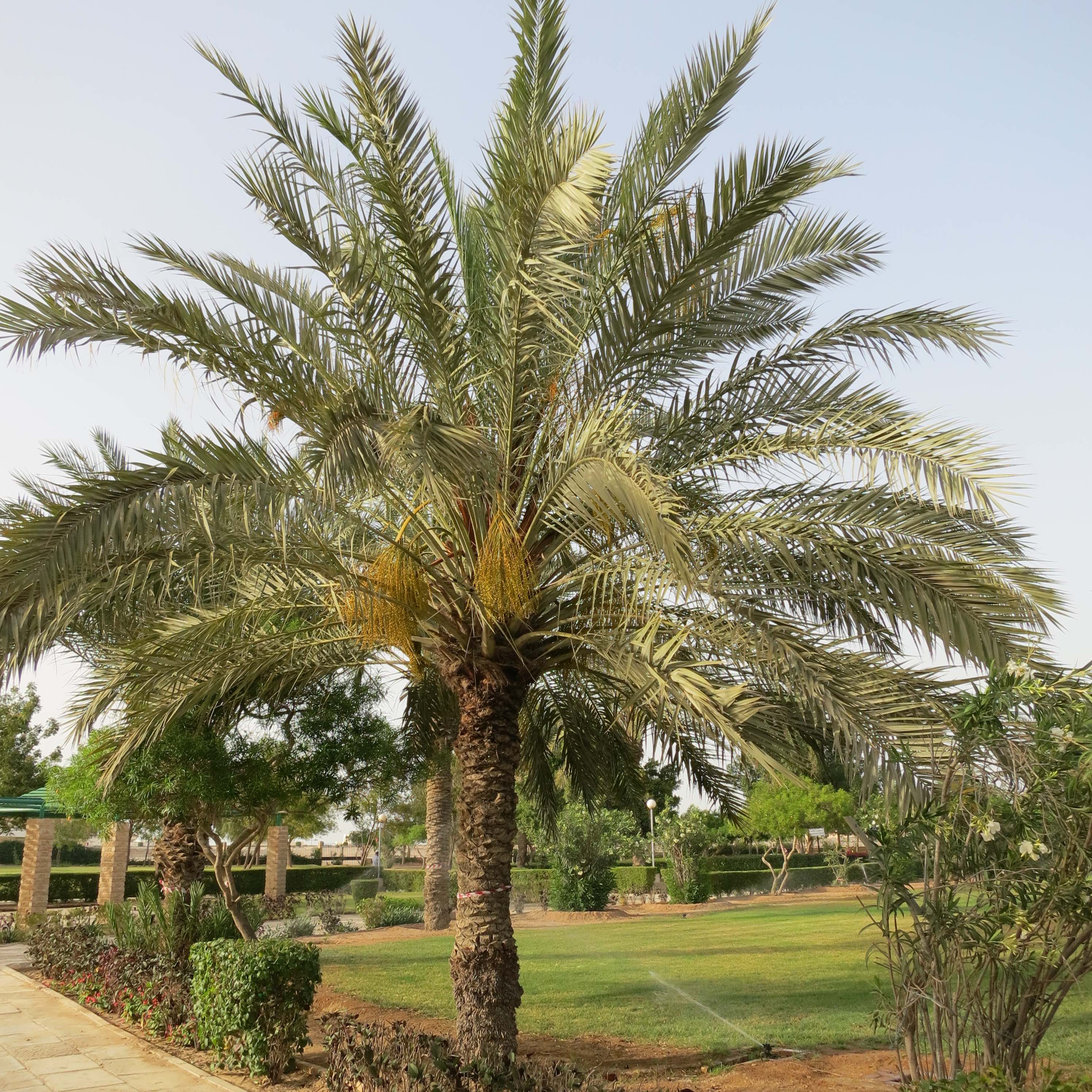

Supplement: S2 File — The images depict morphological characteristics of date palm trees growing in the State of Qatar. (ZIP) [file pone.0207299.s002.zip › Additional_Dataset_2_reduced/010 D.jpg]

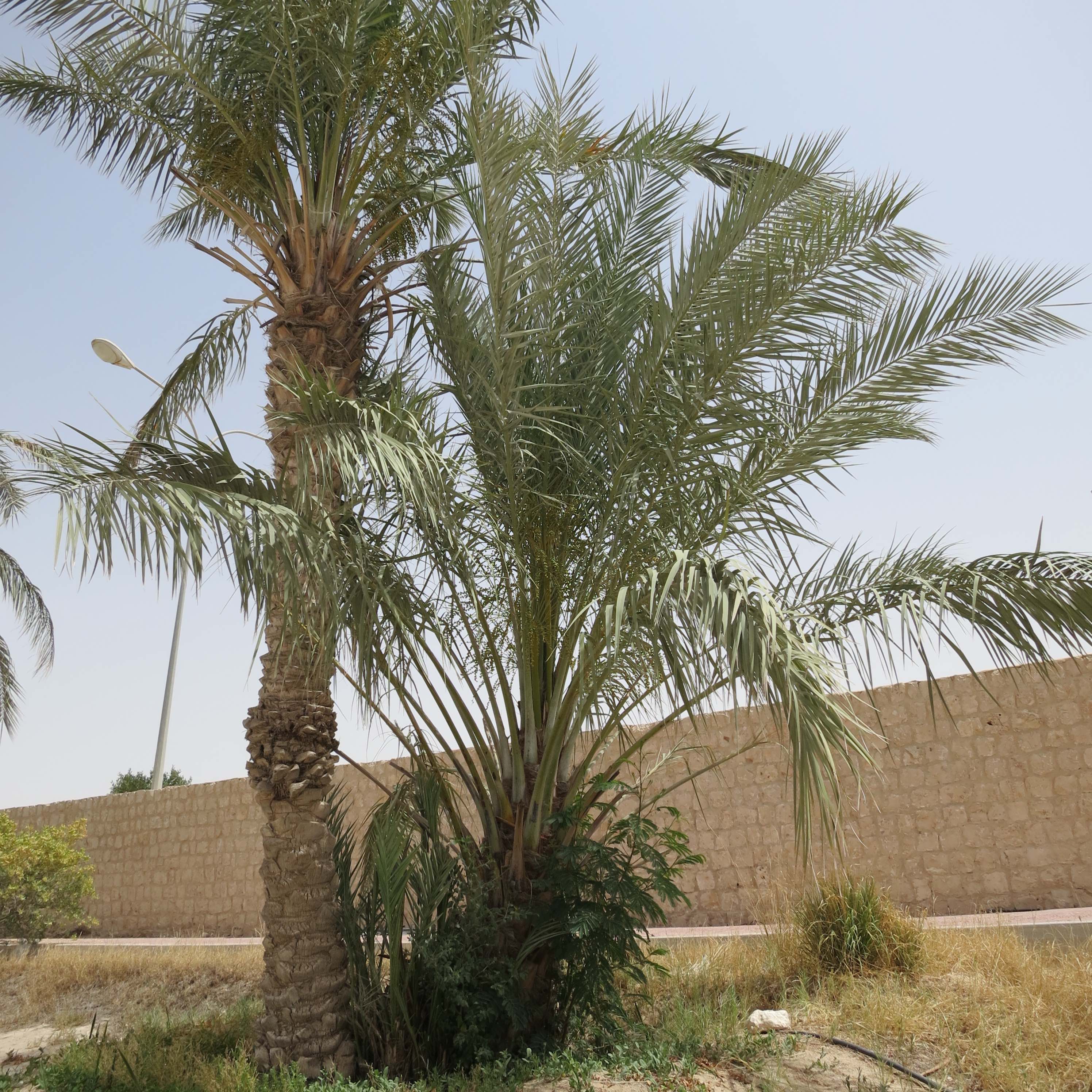

Supplement: S2 File — The images depict morphological characteristics of date palm trees growing in the State of Qatar. (ZIP) [file pone.0207299.s002.zip › Additional_Dataset_2_reduced/036 G.jpg]

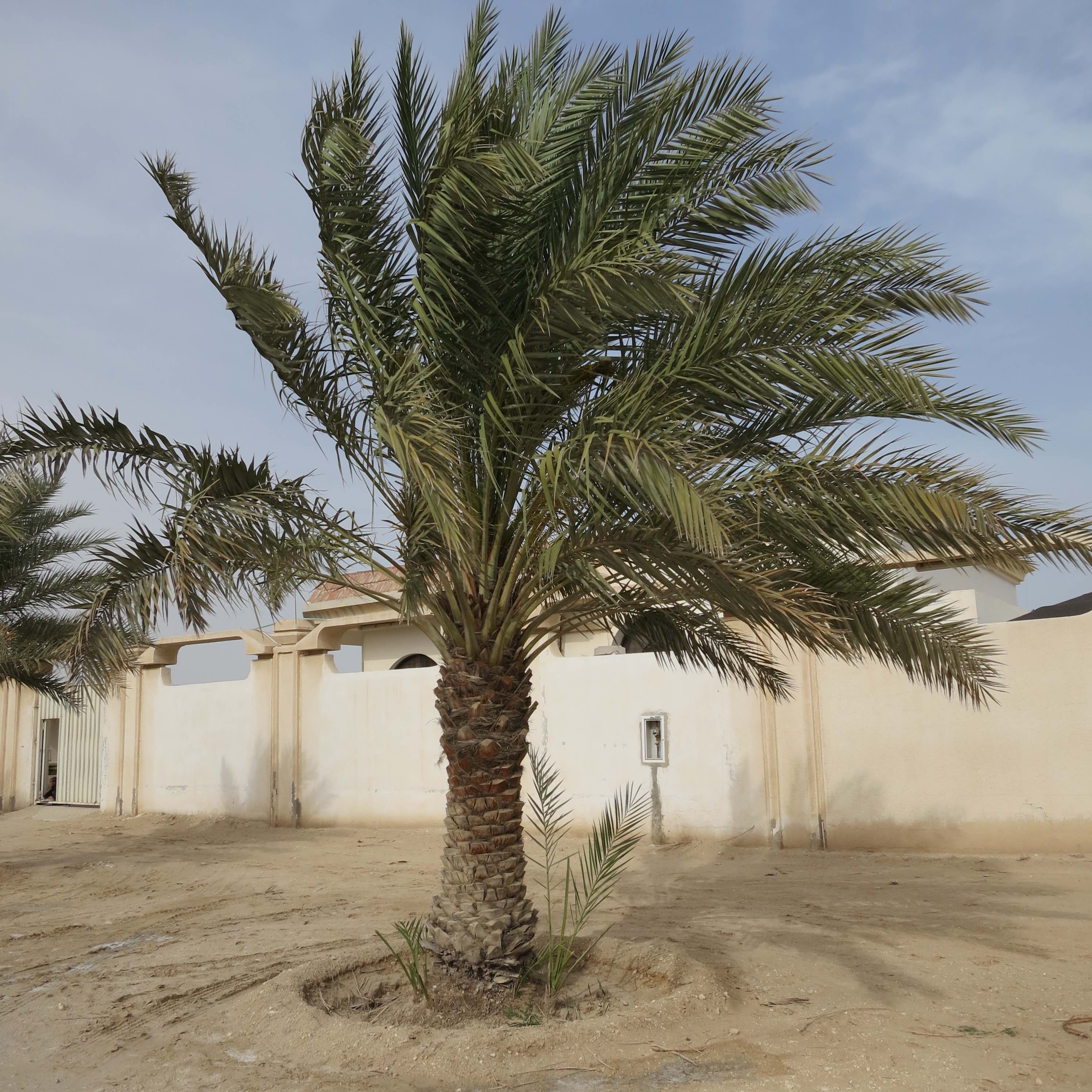

Supplement: S2 File — The images depict morphological characteristics of date palm trees growing in the State of Qatar. (ZIP) [file pone.0207299.s002.zip › Additional_Dataset_2_reduced/009 E.jpg]

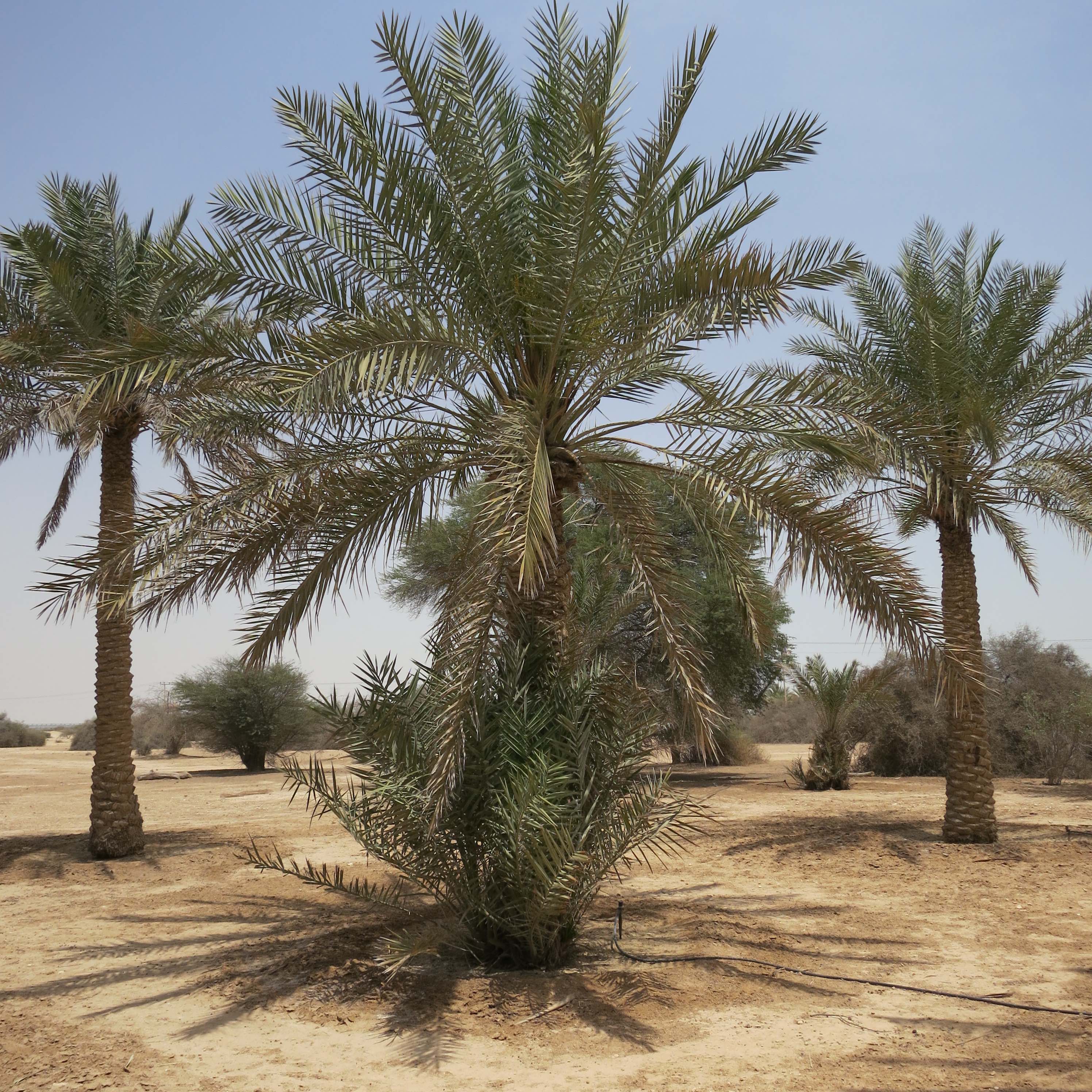

Supplement: S2 File — The images depict morphological characteristics of date palm trees growing in the State of Qatar. (ZIP) [file pone.0207299.s002.zip › Additional_Dataset_2_reduced/012 G.jpg]

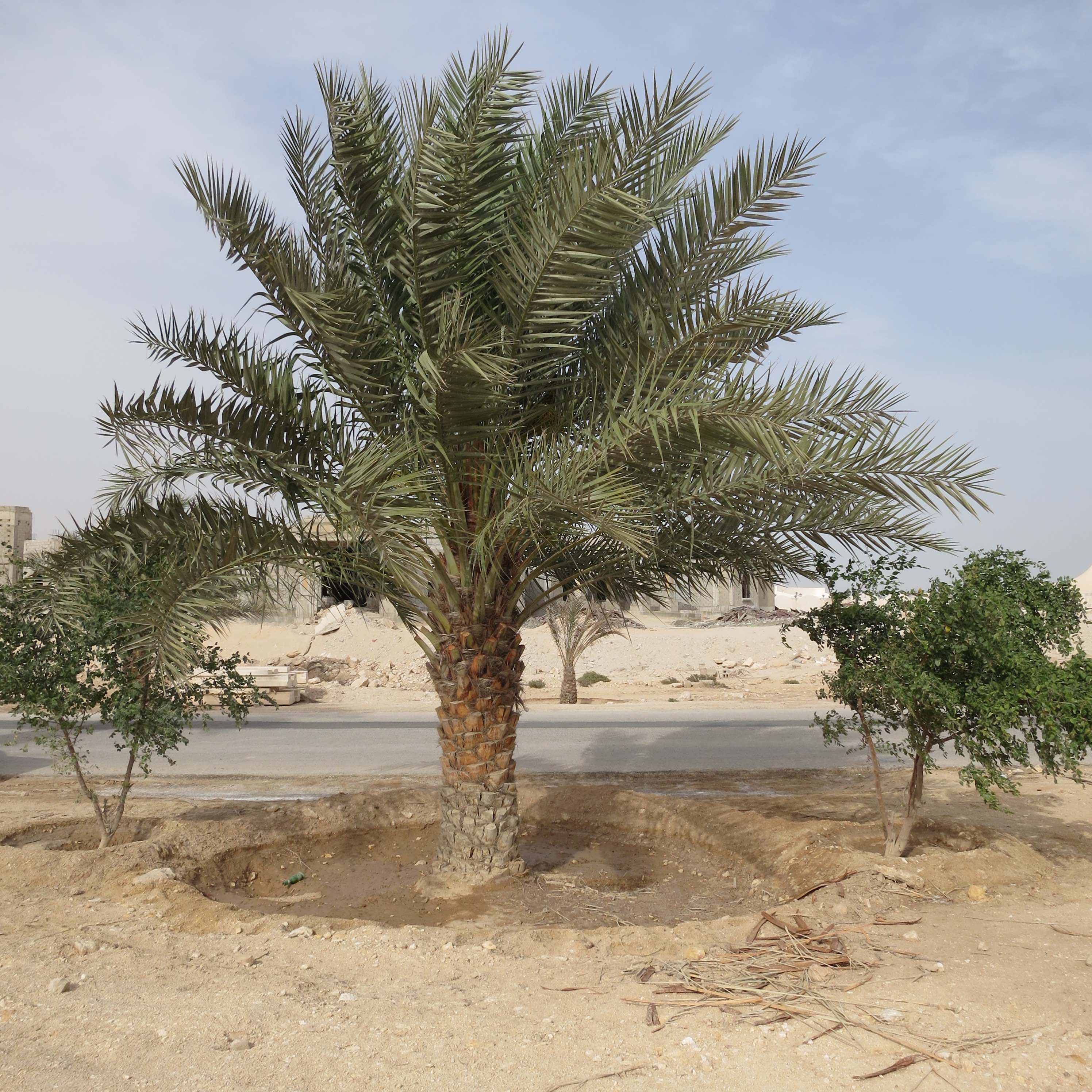

Supplement: S2 File — The images depict morphological characteristics of date palm trees growing in the State of Qatar. (ZIP) [file pone.0207299.s002.zip › Additional_Dataset_2_reduced/010 E.jpg]

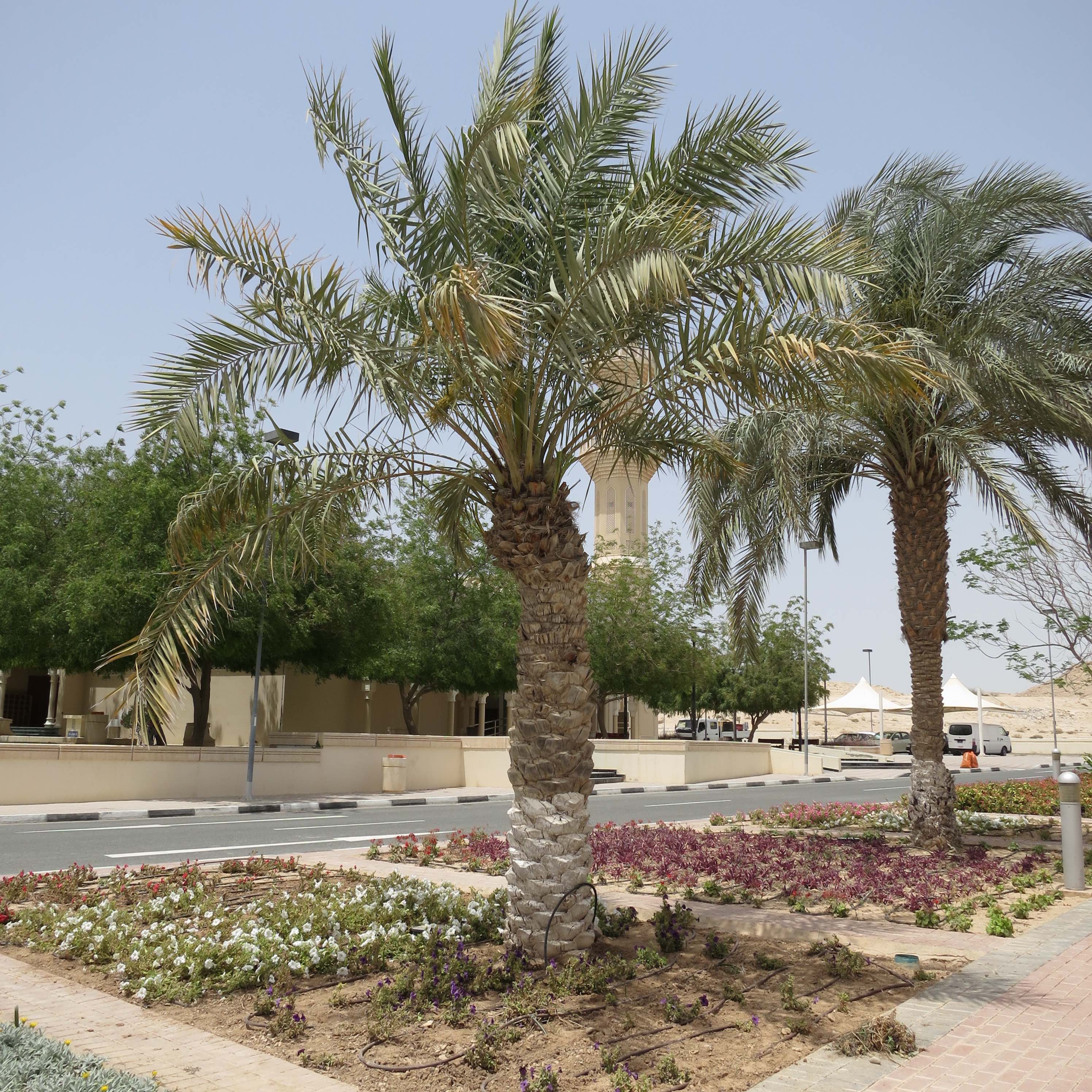

Supplement: S2 File — The images depict morphological characteristics of date palm trees growing in the State of Qatar. (ZIP) [file pone.0207299.s002.zip › Additional_Dataset_2_reduced/032 G.jpg]

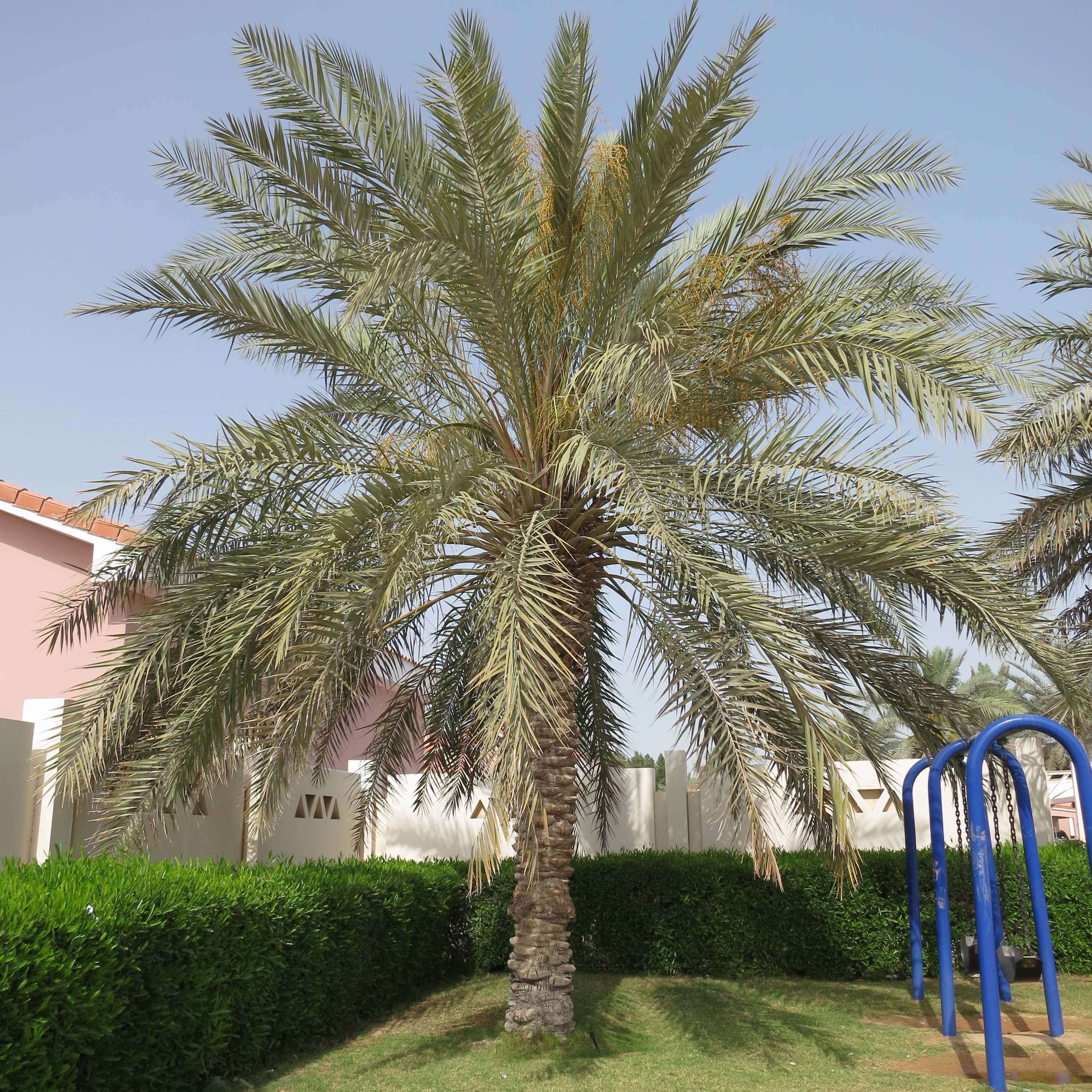

Supplement: S2 File — The images depict morphological characteristics of date palm trees growing in the State of Qatar. (ZIP) [file pone.0207299.s002.zip › Additional_Dataset_2_reduced/009 A.jpg]

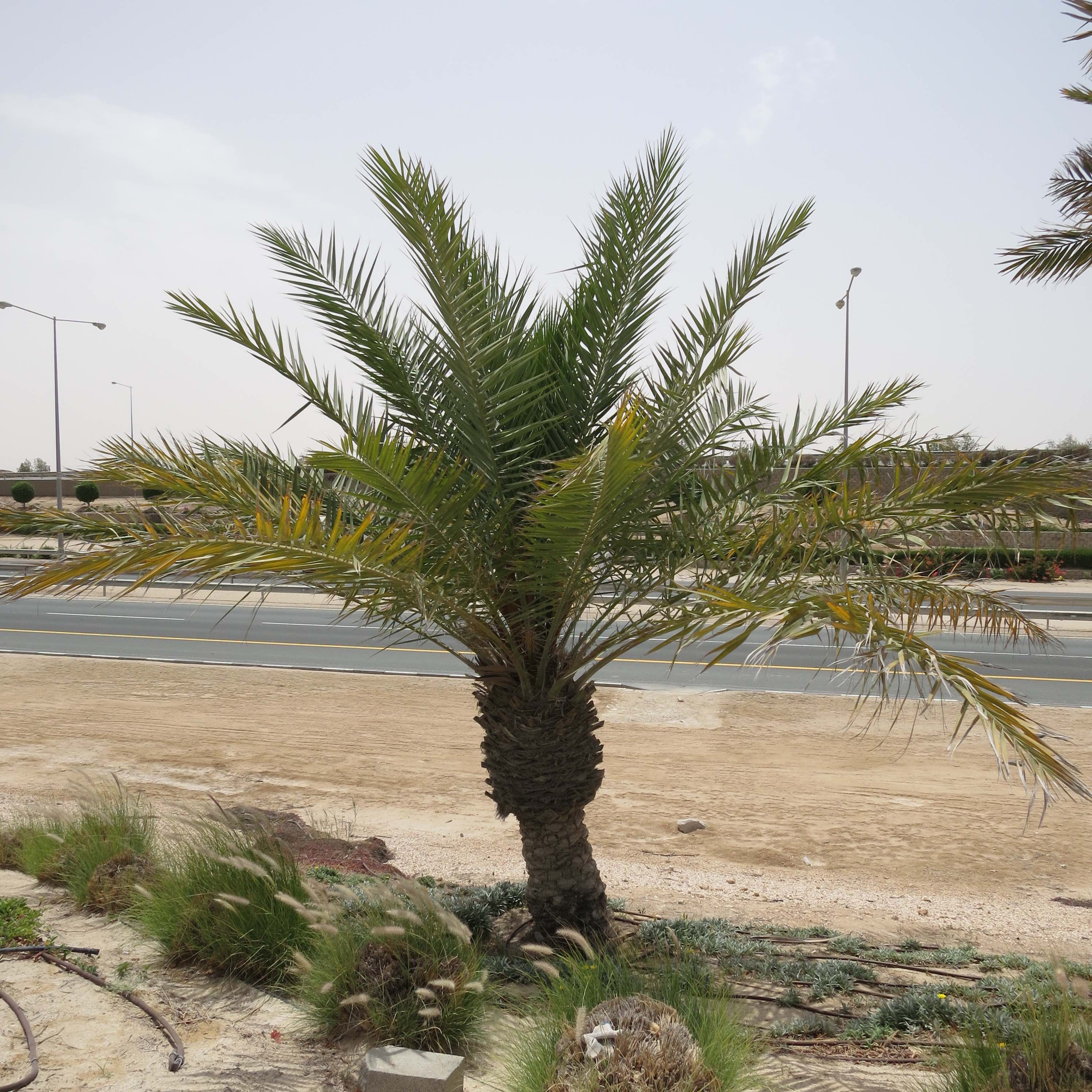

Supplement: S2 File — The images depict morphological characteristics of date palm trees growing in the State of Qatar. (ZIP) [file pone.0207299.s002.zip › Additional_Dataset_2_reduced/030 G.jpg]

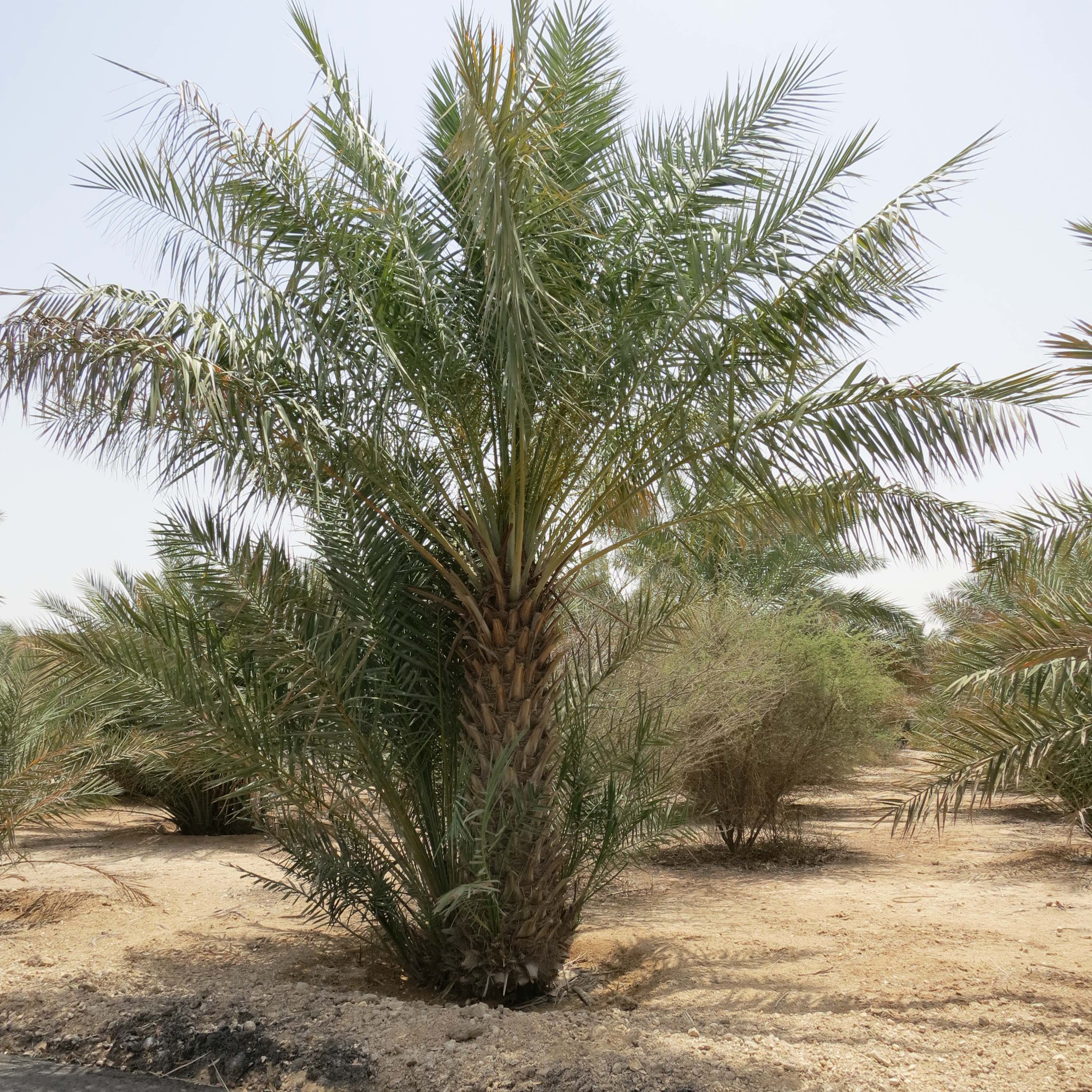

Supplement: S2 File — The images depict morphological characteristics of date palm trees growing in the State of Qatar. (ZIP) [file pone.0207299.s002.zip › Additional_Dataset_2_reduced/014 G.jpg]

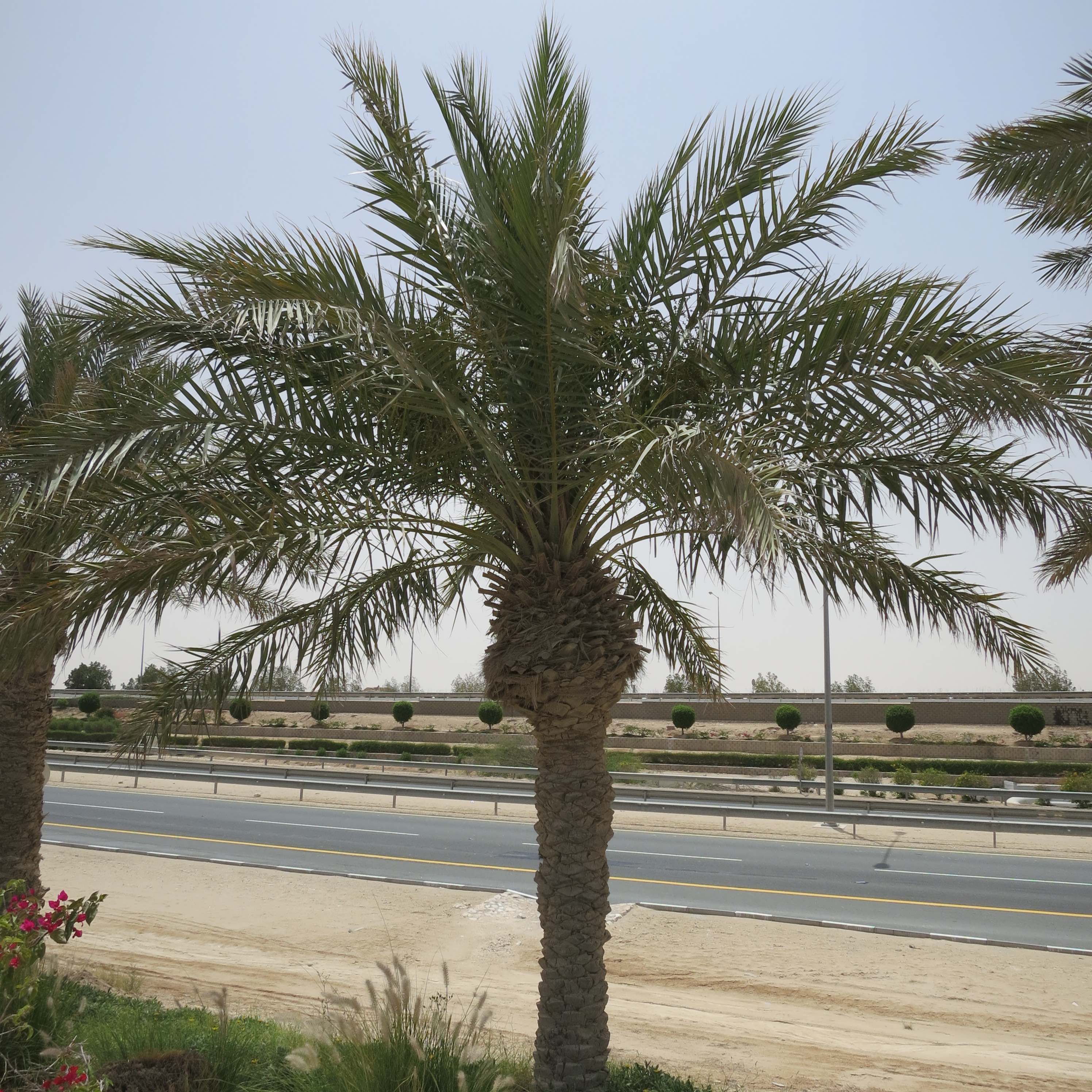

Supplement: S2 File — The images depict morphological characteristics of date palm trees growing in the State of Qatar. (ZIP) [file pone.0207299.s002.zip › Additional_Dataset_2_reduced/029 G.jpg]

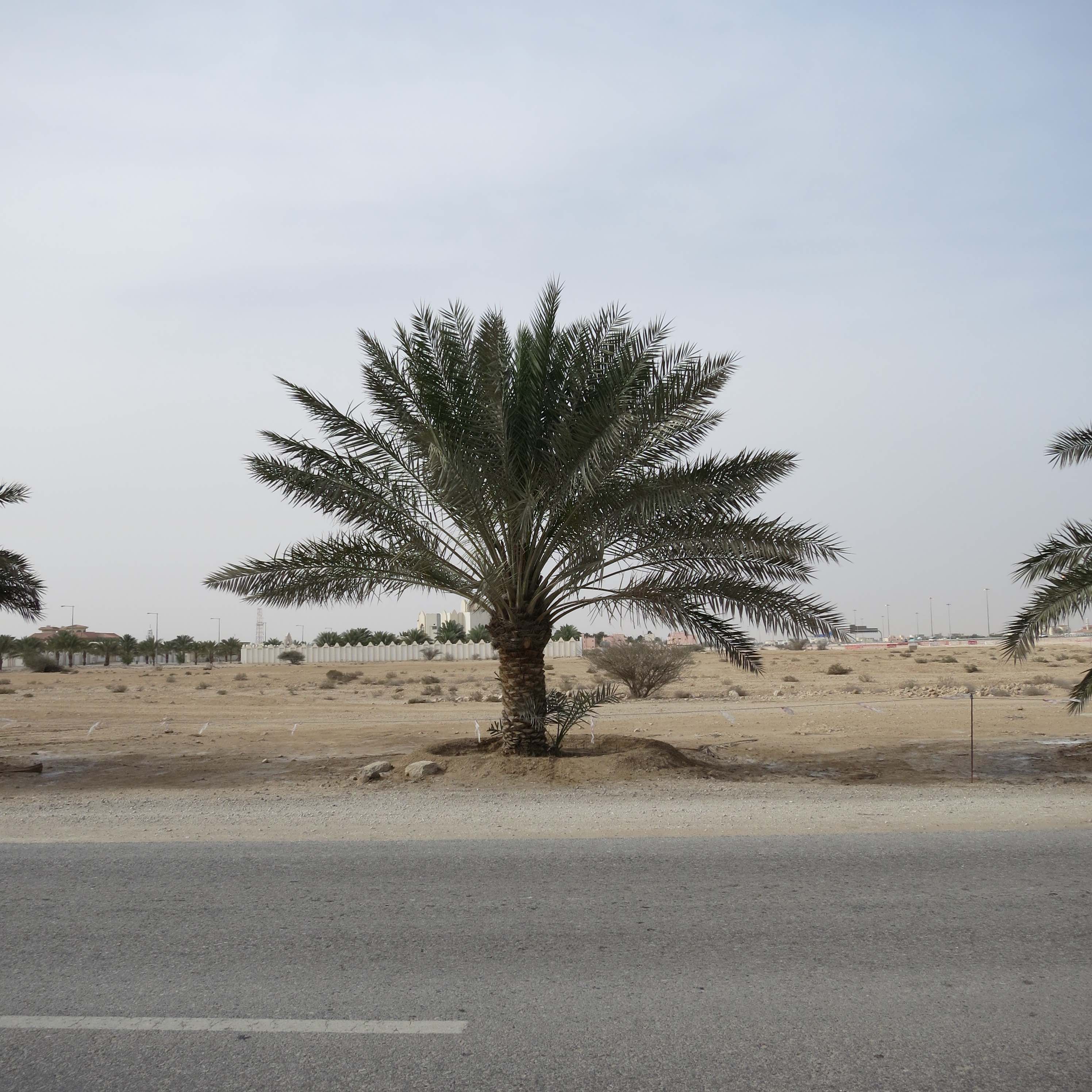

Supplement: S2 File — The images depict morphological characteristics of date palm trees growing in the State of Qatar. (ZIP) [file pone.0207299.s002.zip › Additional_Dataset_2_reduced/008 E.jpg]

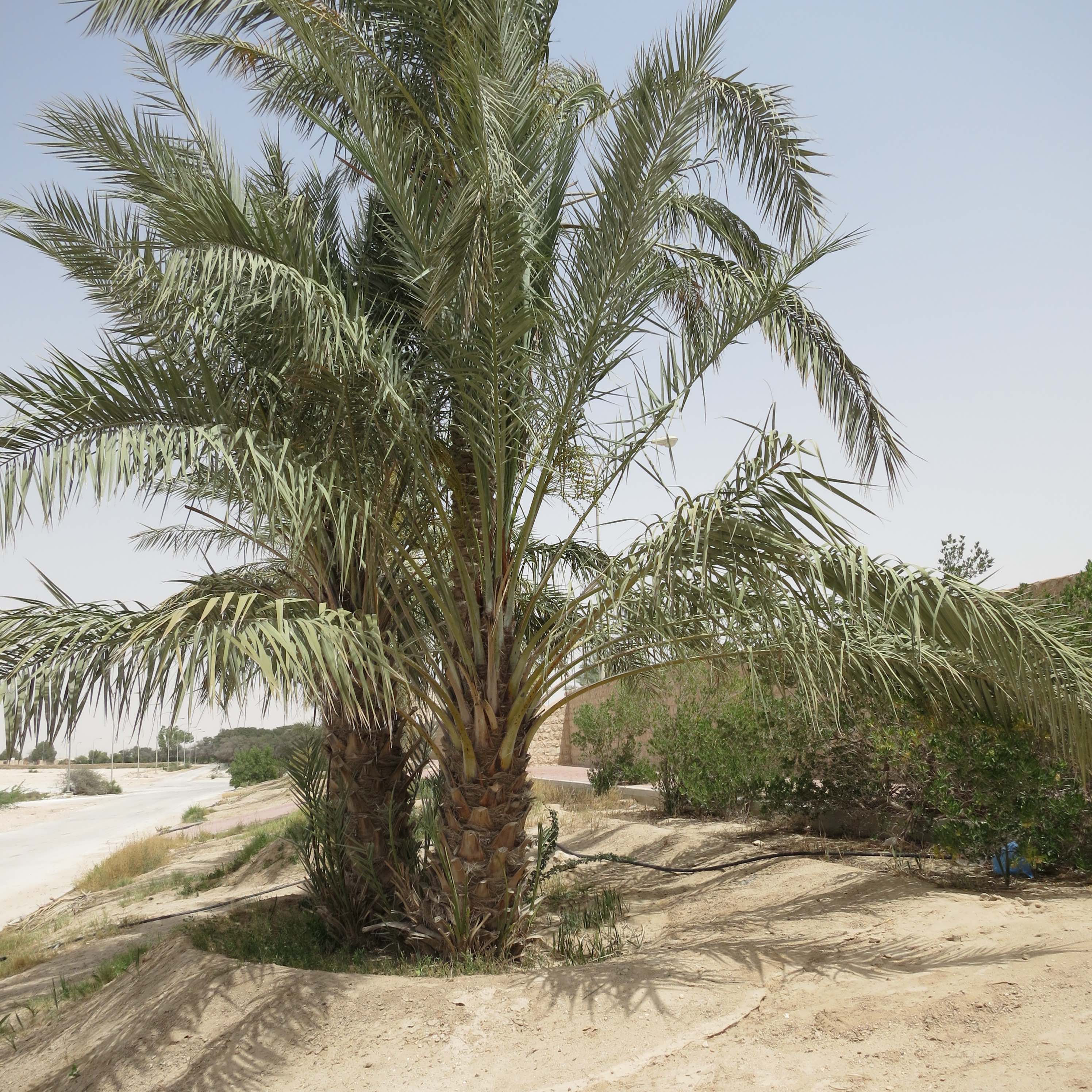

Supplement: S2 File — The images depict morphological characteristics of date palm trees growing in the State of Qatar. (ZIP) [file pone.0207299.s002.zip › Additional_Dataset_2_reduced/037 G.jpg]

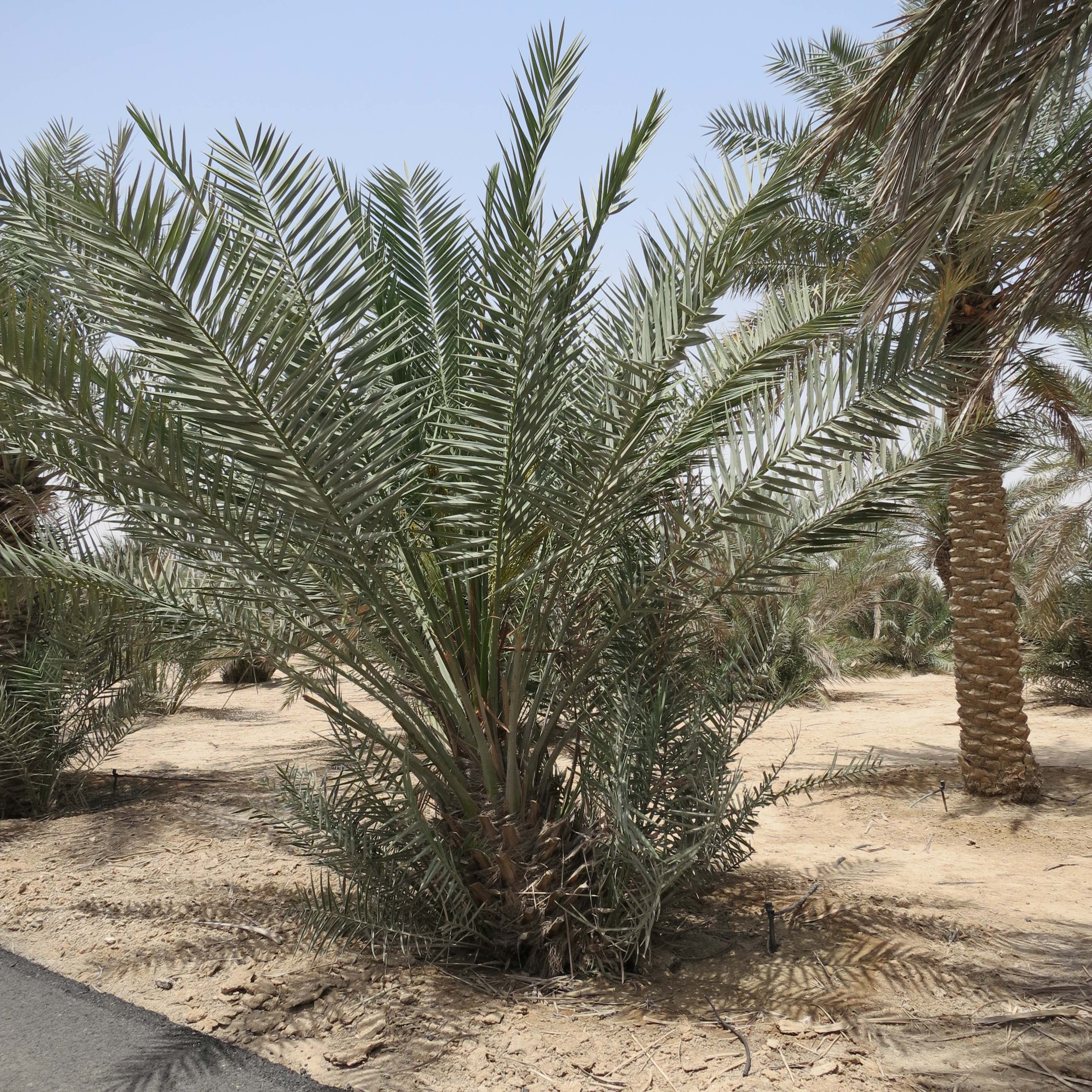

Supplement: S2 File — The images depict morphological characteristics of date palm trees growing in the State of Qatar. (ZIP) [file pone.0207299.s002.zip › Additional_Dataset_2_reduced/013 G.jpg]

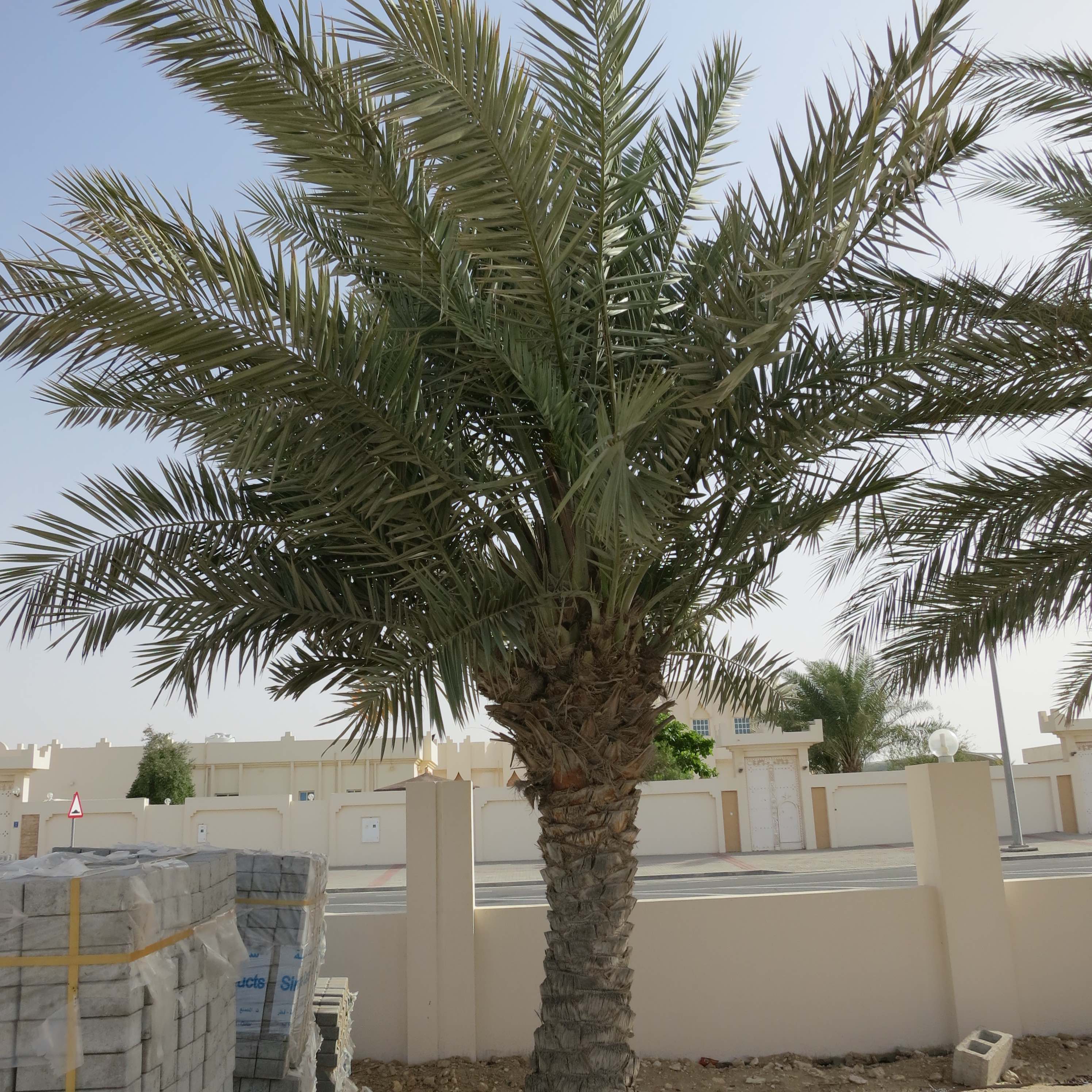

Supplement: S2 File — The images depict morphological characteristics of date palm trees growing in the State of Qatar. (ZIP) [file pone.0207299.s002.zip › Additional_Dataset_2_reduced/008 D.jpg]

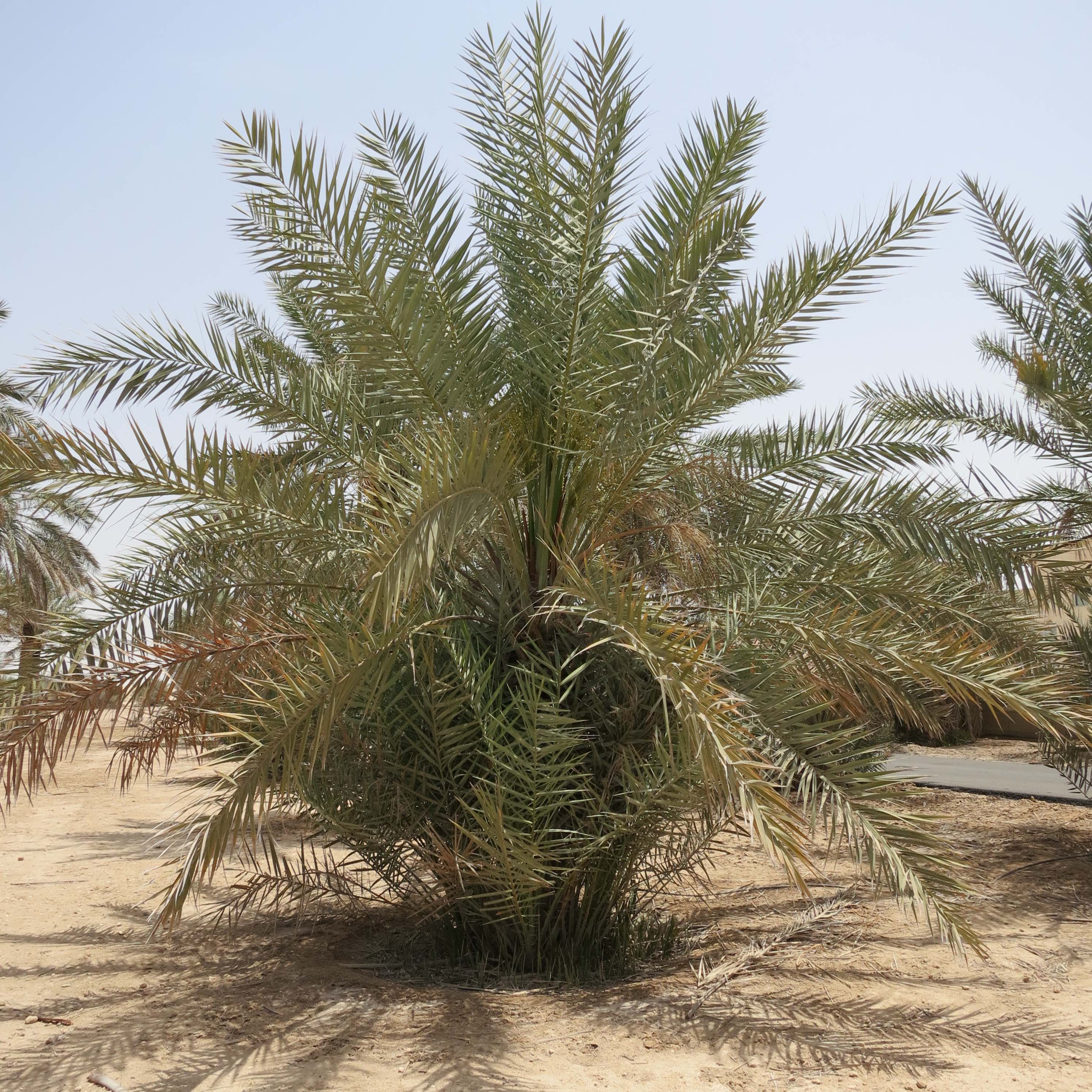

Supplement: S2 File — The images depict morphological characteristics of date palm trees growing in the State of Qatar. (ZIP) [file pone.0207299.s002.zip › Additional_Dataset_2_reduced/011 G.jpg]

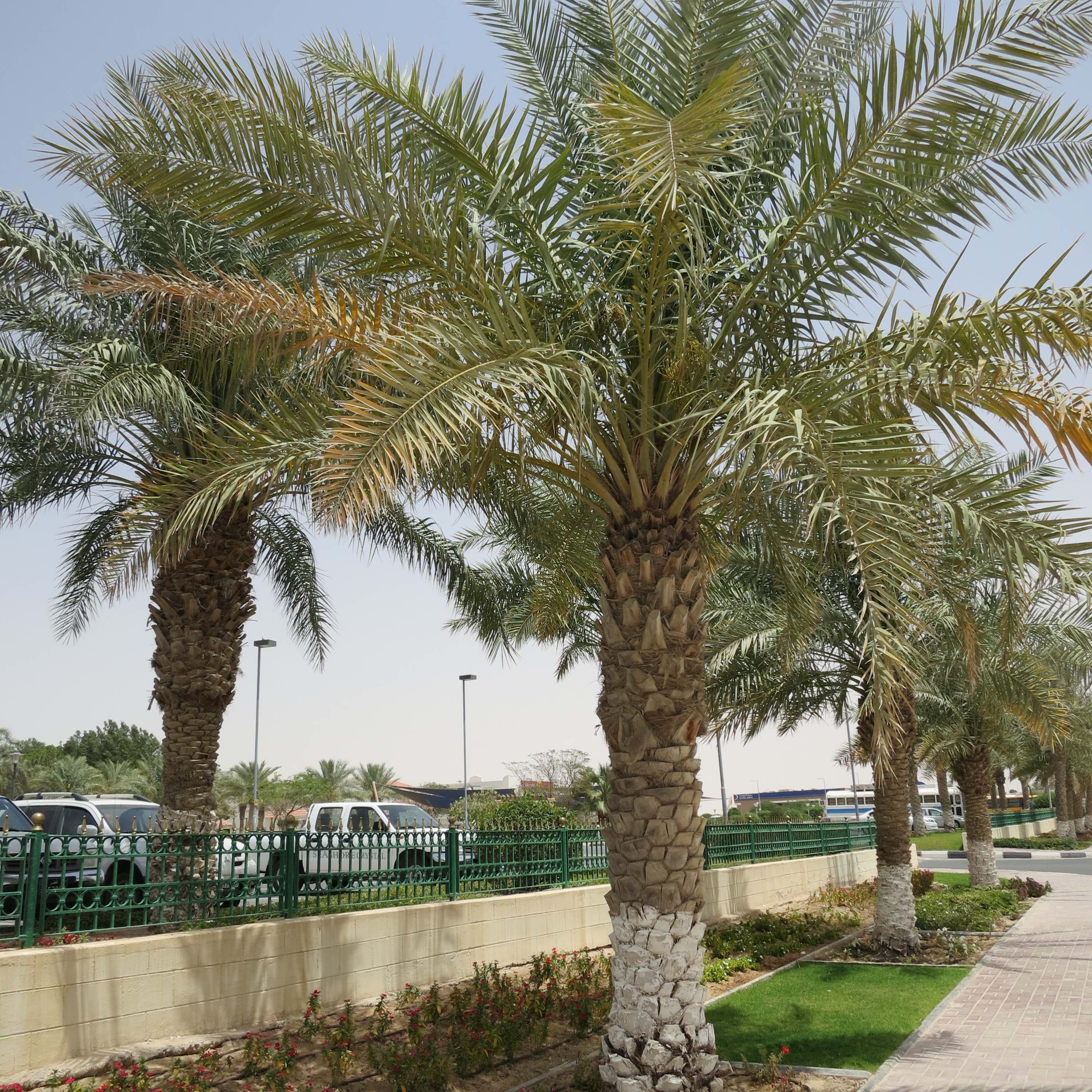

Supplement: S2 File — The images depict morphological characteristics of date palm trees growing in the State of Qatar. (ZIP) [file pone.0207299.s002.zip › Additional_Dataset_2_reduced/035 G.jpg]

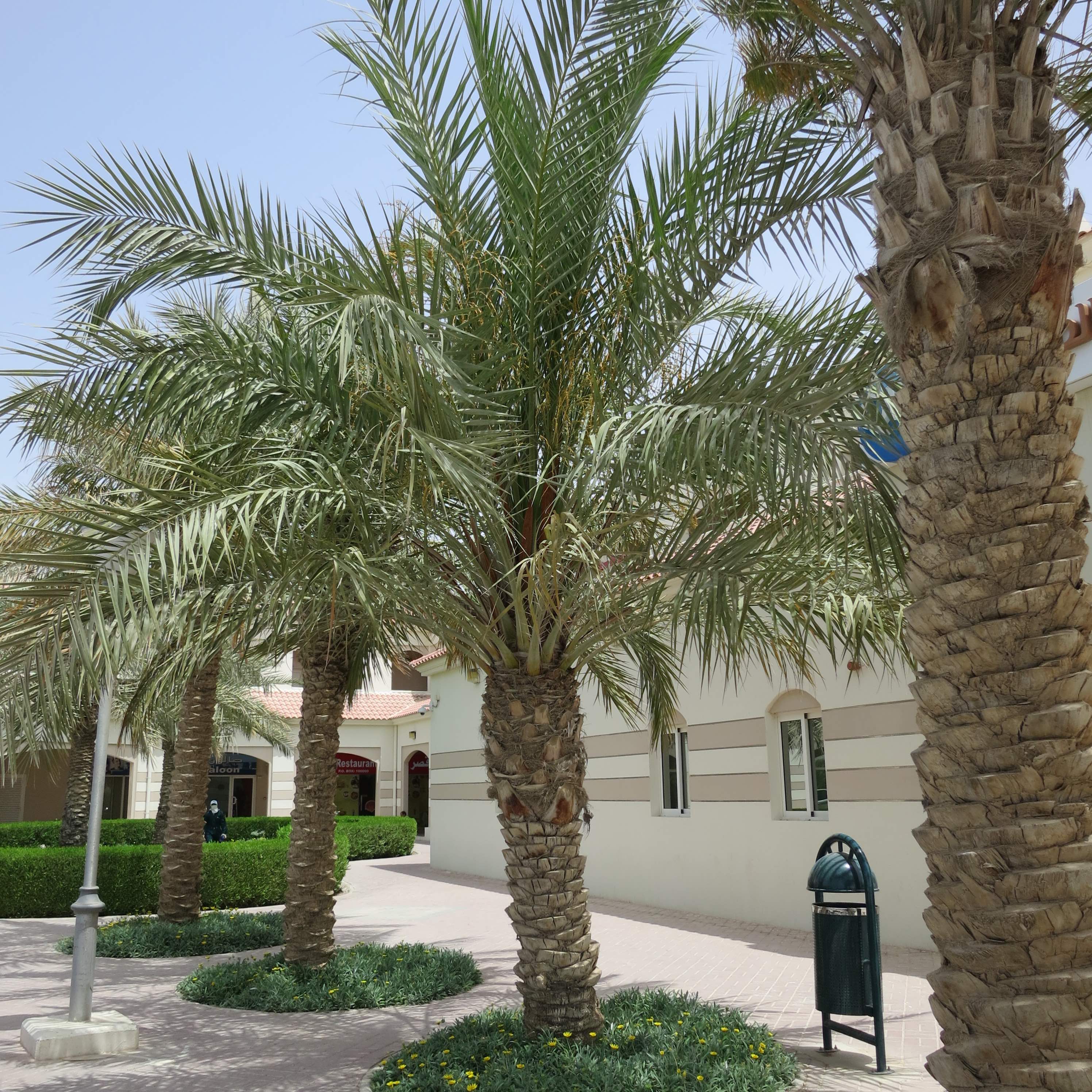

Supplement: S2 File — The images depict morphological characteristics of date palm trees growing in the State of Qatar. (ZIP) [file pone.0207299.s002.zip › Additional_Dataset_2_reduced/031 G.jpg]

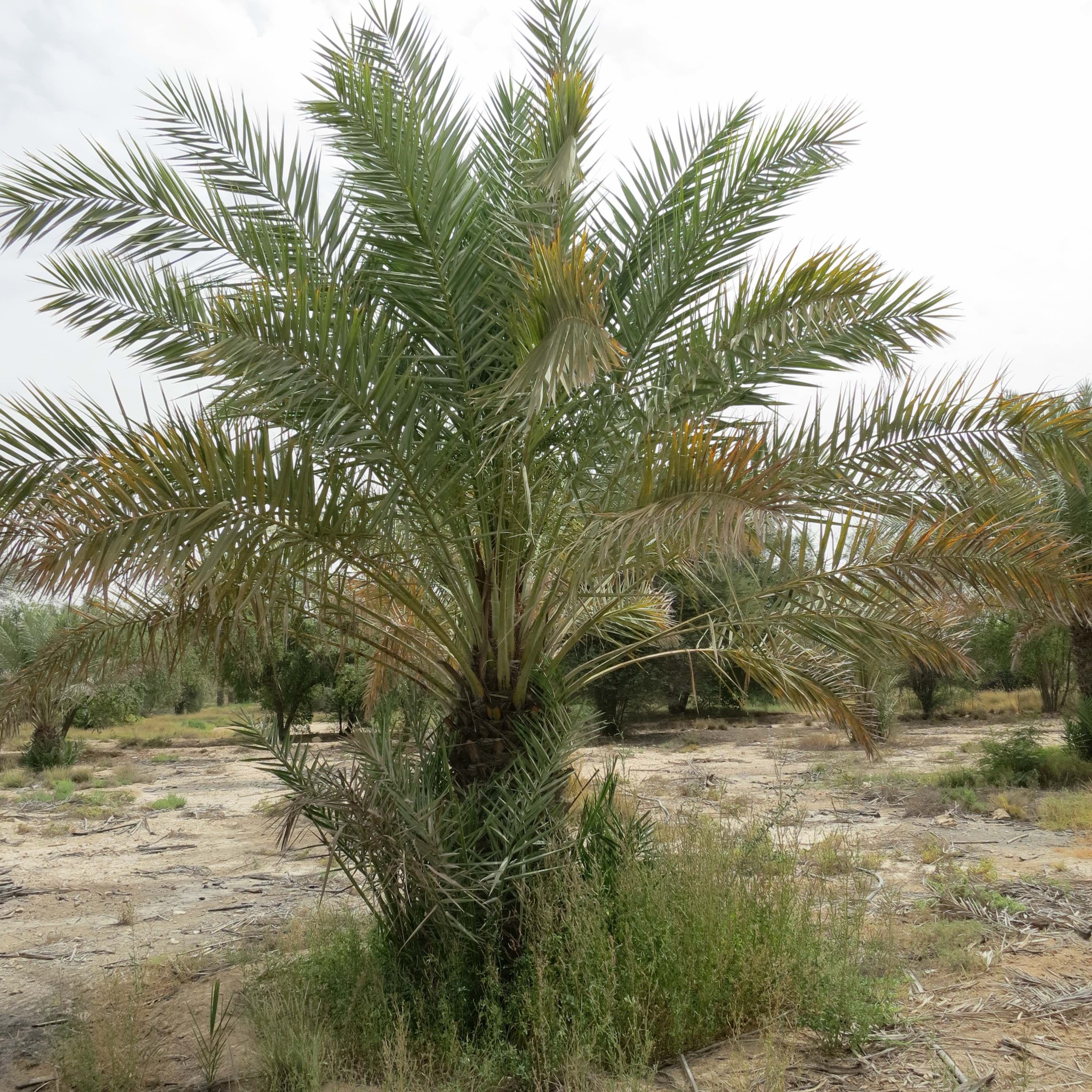

Supplement: S2 File — The images depict morphological characteristics of date palm trees growing in the State of Qatar. (ZIP) [file pone.0207299.s002.zip › Additional_Dataset_2_reduced/028 G.jpg]

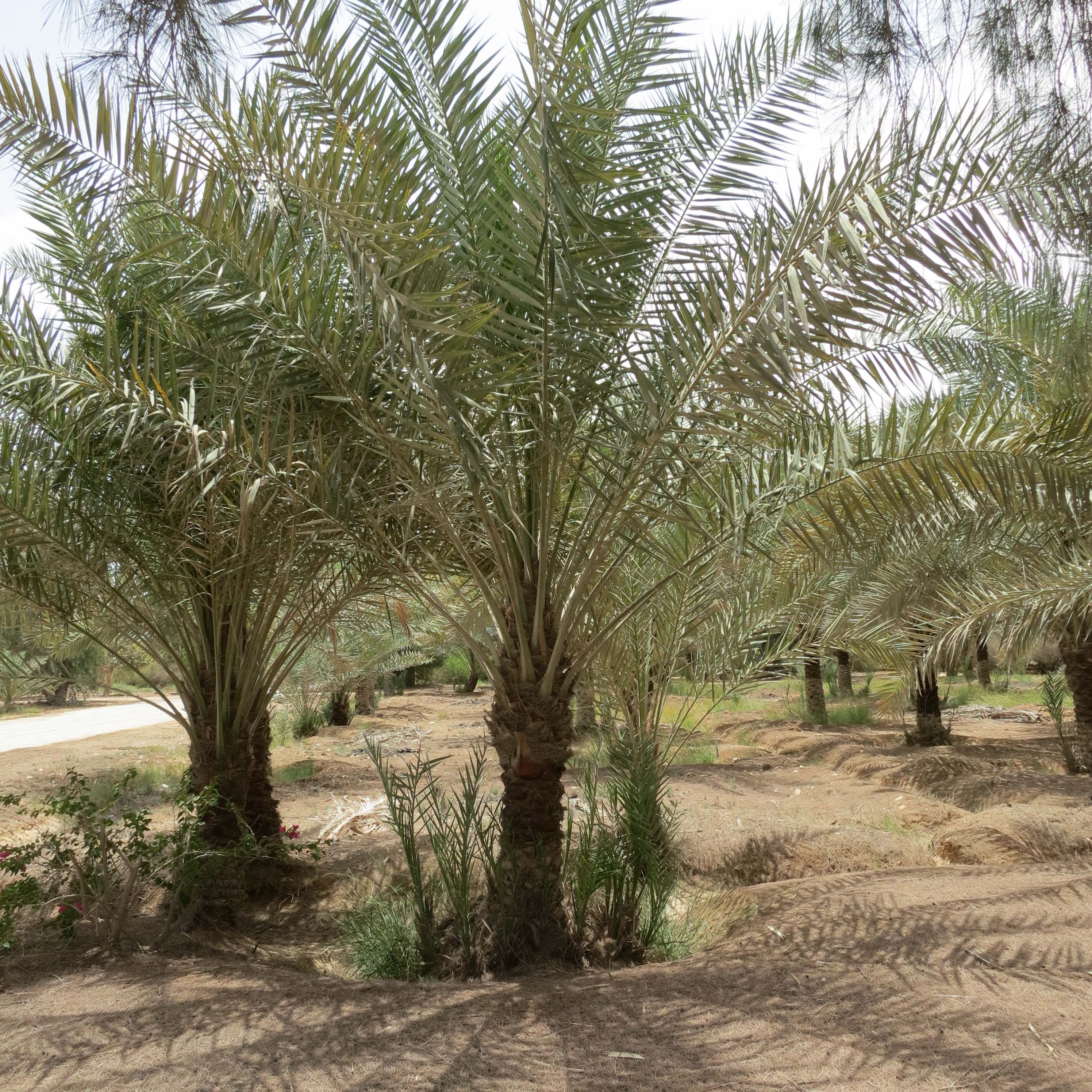

Supplement: S2 File — The images depict morphological characteristics of date palm trees growing in the State of Qatar. (ZIP) [file pone.0207299.s002.zip › Additional_Dataset_2_reduced/008 B.jpg]

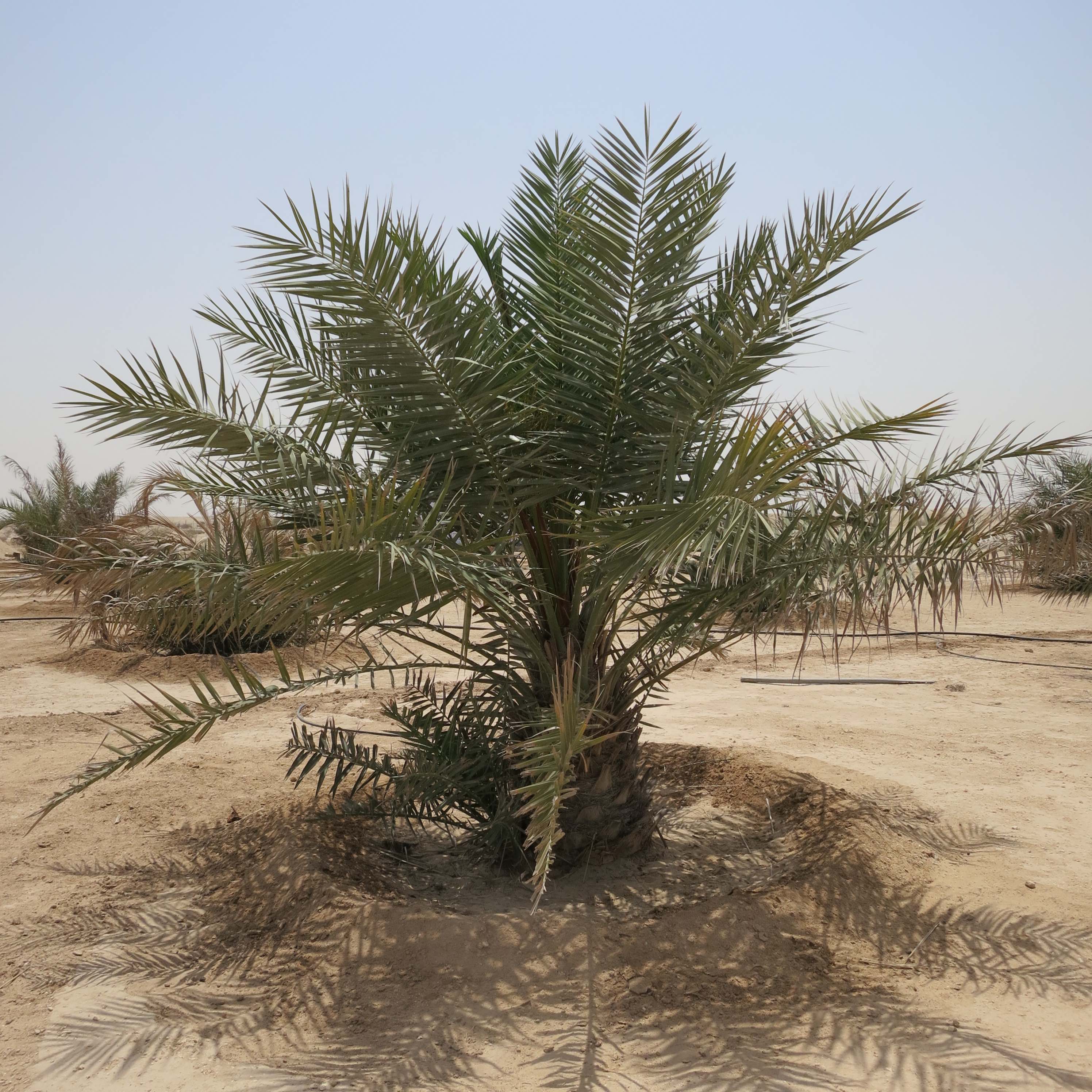

Supplement: S2 File — The images depict morphological characteristics of date palm trees growing in the State of Qatar. (ZIP) [file pone.0207299.s002.zip › Additional_Dataset_2_reduced/017 G.jpg]

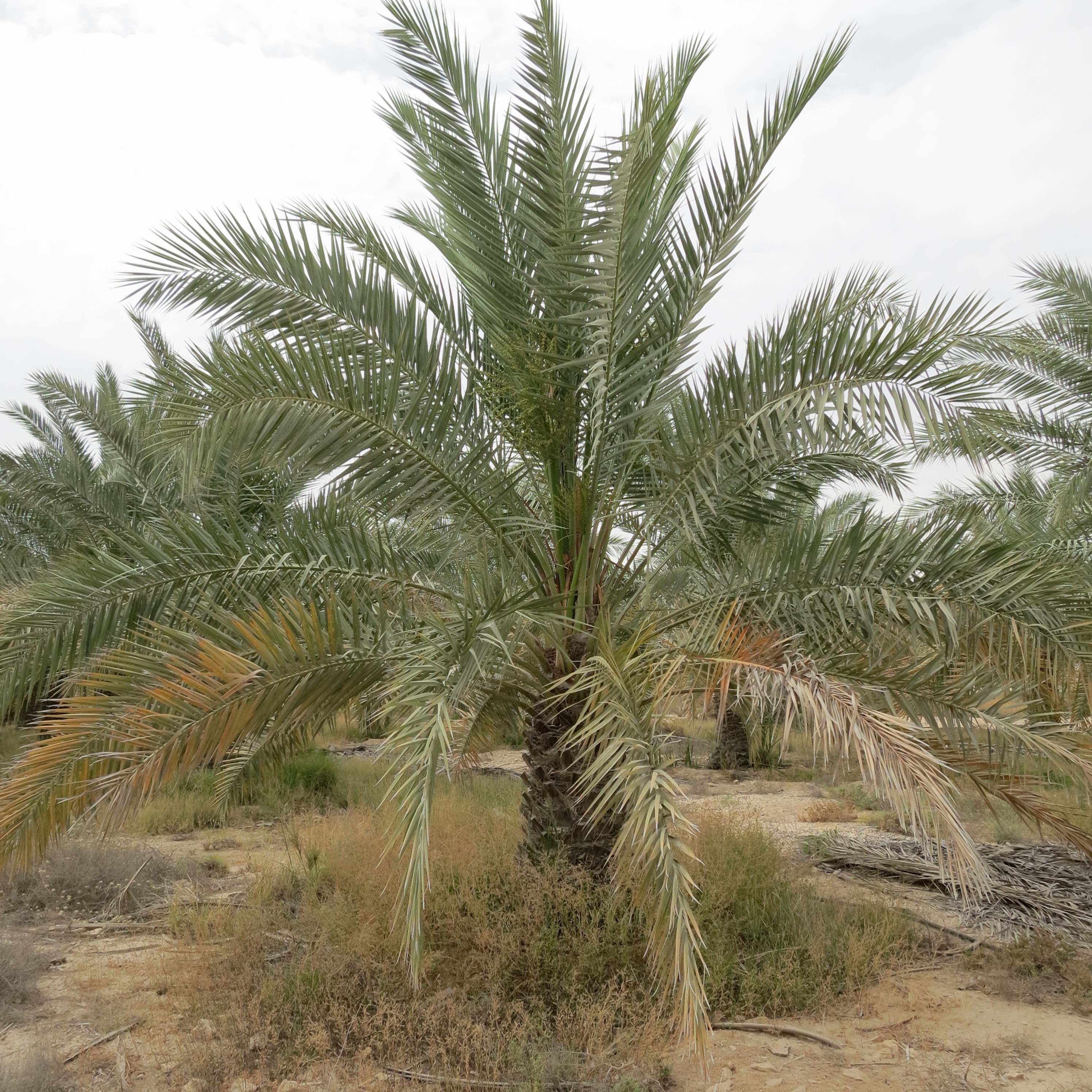

Supplement: S2 File — The images depict morphological characteristics of date palm trees growing in the State of Qatar. (ZIP) [file pone.0207299.s002.zip › Additional_Dataset_2_reduced/033 G.jpg]

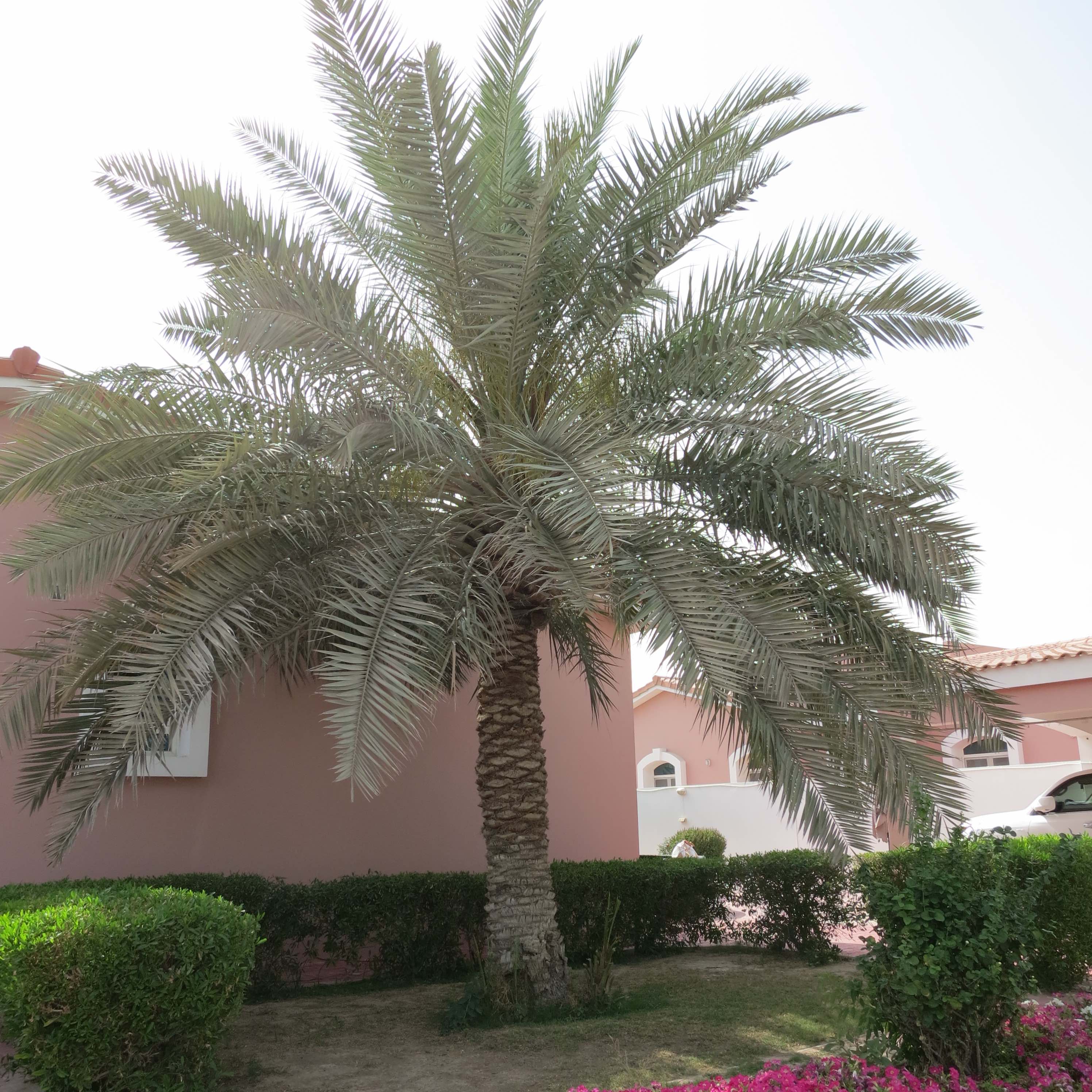

Supplement: S2 File — The images depict morphological characteristics of date palm trees growing in the State of Qatar. (ZIP) [file pone.0207299.s002.zip › Additional_Dataset_2_reduced/008 A.jpg]

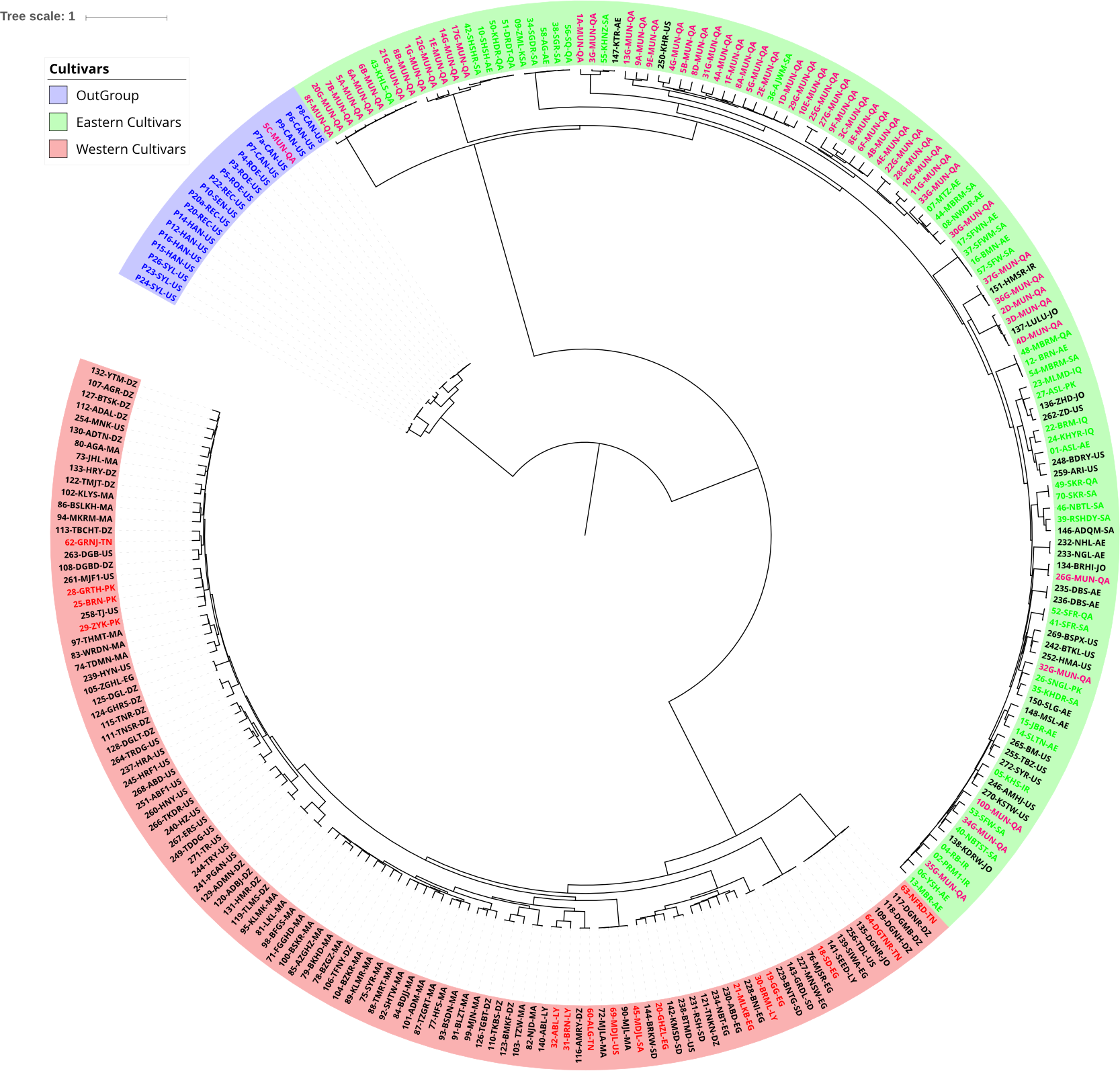

Supplement: S1 Fig — The tree highlights the clustering of date palm trees from the State of Qatar with common date palm cultivars. Date palm trees collected in the State of Qatar are labeled in pink. The edge length represents the Identity-By-State distances. The highlighted clusters in the tree: Outgroup (samples from other Phoenix Species); Eastern cultivars (green) originating in Arabian Gulf and Western cultivars originating in North Africa (blue). The labels are colored based on their annotations from our previous work [22]. Green labels indicate cultivars were annotated as Eastern cultivars; Red labels indicate cultivars were annotated as Western cultivars; and black labels are new cultivars used in this study. (TIFF) [file pone.0207299.s006.tiff]
